# Supplementary material for: Tyramine Acts Downstream of Neuronal XBP-1s to Coordinate Inter-tissue UPRER Activation and Behavior in C. elegans
Source: Dev Cell. 2020 Dec 21;55(6):754–770.e6. doi: 10.1016/j.devcel.2020.10.024 (PMC7758879; doi:10.1016/j.devcel.2020.10.024)
Supplement: Document S1. Figures S1–S7 [file mmc1.pdf]

Developmental Cell, Volume *55*

Supplemental Information

Tyramine Acts Downstream of Neuronal XBP-1s to Coordinate Inter-tissue UPR^{ER} Activation and Behavior in *C. elegans*

Neşem P. Özbey, Soudabeh Imanikia, Christel Krueger, Iris Hardege, Julia Morud, Ming Sheng, William R. Schafer, M. Olivia Casanueva, and Rebecca C. Taylor

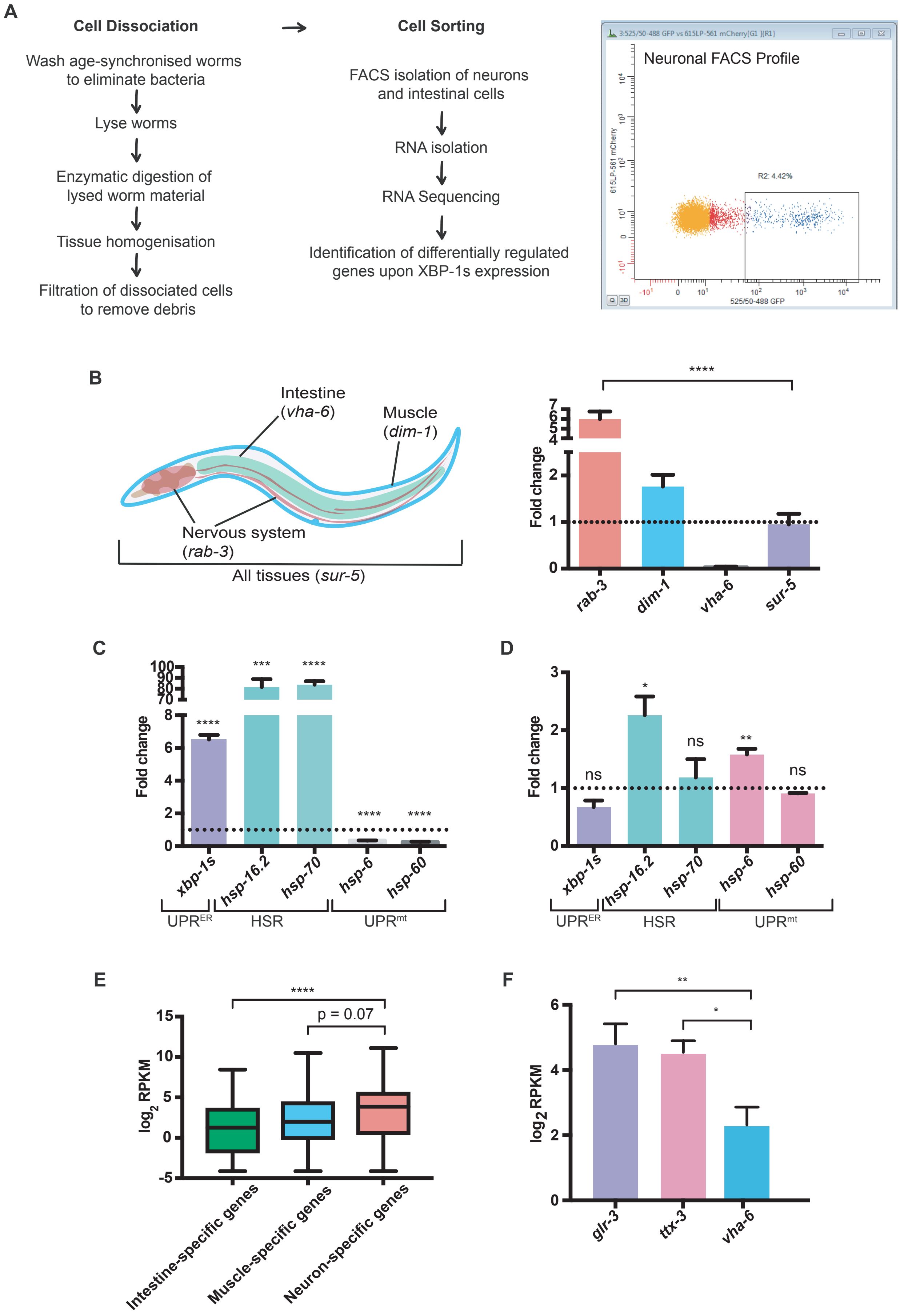
A
Cell Dissociation
Wash age-synchronised worms to eliminate bacteria
Lyse worms
Enzymatic digestion of lysed worm material
Tissue homogenisation
Filtration of dissociated cells to remove debris
Cell Sorting
FACS isolation of neurons and intestinal cells
RNA isolation
RNA Sequencing
Identification of differentially regulated genes upon XBP-1s expression
Neuronal FACS Profile
R2: 4.42%
615LP-561 mCherry
525/50-488 GFP
B
Intestine (vha-6)
Muscle (dim-1)
Nervous system (rab-3)
All tissues (sur-5)
****
Fold change
rab-3
dim-1
vha-6
sur-5
C
Fold change
***
****
xbp-1s
hsp-16.2
hsp-70
hsp-6
hsp-60
UPRER
HSR
UPRmt
D
Fold change
ns
*
**
xbp-1s
hsp-16.2
hsp-70
hsp-6
hsp-60
UPRER
HSR
UPRmt
E
****
p = 0.07
log2 RPKM
Intestine-specific genes
Muscle-specific genes
Neuron-specific genes
F
**
*
log2 RPKM
glr-3
ttx-3
vha-6

Figure S1. Validation of neuron-specific RNA-seq analysis. Related to Figure 1.

(A) Experimental outline of adult cell dissociation for tissue-specific RNA-seq. An example FACS profile for cells prepared for neuronal RNA-seq highlights the GFP population isolated from dissociated *unc-119p::GFP* worms.

(B) Tissue-specific transcript levels determined by RT-qPCR in samples of isolated neuronal cells from *unc-119p::GFP* worms compared to dissociated but unsorted cells of the same strain. Fold change was calculated relative to the all-tissue transcript *sur-5*, and error bars indicate SEM (N=3 biological replicates). Significance was assessed by one-way ANOVA with Dunnett's multiple comparisons test, ****p≤0.0001.

(C) RT-qPCR analysis of *xbp-1s, hsp-16.2, hsp-70, hsp-6* and *hsp-60* transcripts in dissociated and FACS-sorted GFP-positive neuronal cells from *unc-119p::GFP* worms, versus dissociated and FACS-sorted GFP-negative non-neuronal cells. Fold change was calculated for each transcript relative to wild type, and error bars indicate SEM (N=3 biological replicates). Significance of the difference between levels of each transcript in wild type and neuronal *xbp-1s*-expressing animals was assessed by unpaired student's t-test, ***p≤0.001, ****p≤0.0001.

(D) RT-qPCR analysis of *xbp-1s, hsp-16.2, hsp-70, hsp-6* and *hsp-60* transcripts in dissociated and FACS-sorted, recombined cell samples from *unc-119p::GFP* worms, versus cells that were dissociated but not FACS-sorted. Fold change was calculated for each transcript relative to wild type, and error bars indicate SEM (N=3 biological replicates). Significance of the difference between levels of each transcript in wild type and neuronal *xbp-1s*-expressing animals was assessed by unpaired student's t-test, *p≤0.05, **p≤0.01, ns=not significant.

(E) Expression levels of tissue-specific genes (intestine, muscle and nervous system) in the neuronal RNA-seq dataset of *unc-119p::GFP; rab-3p::xbp-1s* animals. Tissue-

specific genes were identified using the Princeton tissue-specific database. RPKM = reads per kilobase per million mapped reads, N=3 biological replicates, and error bars indicate SEM. Significance was calculated relative to neuron-specific genes by one-way ANOVA with Dunnett's multiple comparisons test, ****p≤0.0001.

(F) Levels of 2 transcripts expressed in no more than two pairs of neurons in the neuronal RNA-seq dataset of *unc-119p::GFP; rab-3p::xbp-1s* animals. N=3 biological replicates, error bars indicate SEM. Significance was calculated relative to levels of the intestinal (non-neuronal) transcript *vha-6* by one-way ANOVA with Dunnett's multiple comparisons test, *p≤0.05, **p≤0.01.

XBP-1-dependent INDUCIBLE UPR genes

Gene Function	Up in neuronal *xbp-1s*	Down in neuronal *xbp-1s*	Gene names (differentially regulated by neuronal *xbp-1s*)
**COP1 coated vesicle**	1/1	0/1	Y41C4A.11 (Coatomer β subunit)
**Protein disulphide isomerases**	3/4	0/4	C14B9.2, B0403.4, PDI-2
**Chaperones**	7/10	0/7	DNJ-7, DNJ-28, HSP-3, HSP-4, T14G8.3 (HSP70), T24H7.2 (HSP70), Y57A10A.23 (ERp19)
**Ubiquitination**	2/3	0/3	UBC-13, H40L08.1
**Vesicle docking**	3/6	0/6	F55A4.1 (Sec22b), F18H3.4 (integrin), D1014.3 (Snap-1)
**ARF + interacting proteins**	3/7	0/7	F54C8.7 (Arfaptin), Y54G2A.2 (ARL-6 interacting protein 2), K02B12.7 (ARF1-directed GAP)
**Oxidative stress**	2/5	0/5	K01D12.11 (Glutathione S-transferases), Y76B12C.3 (Selenoprotein)
**Cell growth & cell death**	2/5	0/5	Y54G2A.18 (BAP31), C04F12.1 (Piwi-domain containing protein)
**Quality control and ERAD**	2/9	0/9	NSF-1, ERD-2
**Gene expression**	2/9	0/9	F57B10.1 (CREBh), RRF-2
**Lipid & phospholipid metabolism**	2/12	0/12	B0285.9 (choline kinase), ACL-12
**Signal transduction**	1/6	0/6	DLK-1 (MAPK12)
**Ion channels and transporters**	1/9	0/9	T28F3.3 (zinc transporter)
**Sugar modification**	0/7	0/7	
**Sugar synthesis**	0/7	0/7	
**COPII coated vesicle**	0/6	0/6	
**Signal peptidases**	0/5	0/5	
**Calcium homeostasis**	0/5	0/5	
**Mitochondrial homeostasis**	0/4	0/4	
**Protein interaction**	0/4	0/4	
**Clathrin vesicle coat**	0/3	0/3	
**Cytoskeleton**	0/3	0/3	
**Cuticle synthesis**	0/3	0/3	
**Protein folding – other**	0/2	0/2	
**Host defence**	0/2	0/2	
**Phosphate mobilization**	0/1	0/1	
**Purine metabolism**	0/1	0/1	

XBP-1-dependent CONSTITUTIVE UPR genes

Gene Function	Up in neuronal *xbp-1s*	Down in neuronal *xbp-1s*	Gene names (differentially regulated by neuronal *xbp-1s*)
**Sugar transport**	2/2	0/2	H17B01.1, NLP-14 (Neuropeptide-Like Protein)
**Cell fate – other**	1/1	0/1	ZK1073.1 (Differentiation-related gene 1)
**Intracellular trafficking**	2/4	0/4	T12A2.15b (Ca2+-dependent membrane-targeting module), NCS-2 (neuronal calcium sensor protein)
**DNA topoisomerase**	0/2	1/2	ZK1127.7 (DNA topoisomerase II)
**RNA helicase**	0/2	1/2	CGH-1
**RNA synthesis**	0/3	1/3	GLD-2 (poly(A) polymerase)
**Exocytosis**	1/3	0/3	C39F7.2
**Tumor Suppressor**	1/3		MPZ-1 (Multiple PDZ domain protein)
**G protein related**	2/9	1/9	Up: GPB-1 (G beta subunit), GOA-1 (G protein alpha subunit); Down: T19E10.1b (transforming protein etc2)
**Cytoskeleton**	1/9	1/9	Up: PTL-1 (microtubule-associated protein TAU-1a); Down: C53C9.2 (thin filament-associated protein calponin)
**Carbohydrate metabolism**	1/5	0/5	CAH-3 (carbonic anhydrase)
**Lipid metabolism**	1/5	0/5	AAK-2 (metabolic stress-sensing kinase AMPK α2 chain)
**Hormone signalling**	1/5	0/5	INS-30 (Insulin related)
**Cell adhesion & junction**	1/8	0/8	UNC-9 (innexin)
**Ser/Thr kinase**	1/10	0/10	EGL-4 (cyclic-GMP-dependent protein kinase)
**Protein synthesis**	0/5	0/5	
**Ub ligase complex**	0/4	0/4	
**Ub conjugating complex**	0/3	0/3	
**Proteasome regulatory particle**	0/2	0/2	
**Proteases**	0/2	0/2	
**ER & Golgi translocation**	0/8	0/8	
**Vacuole targeting**	0/2	0/2	
**Endocytosis**	0/2	0/2	
**Mitochondrial import**	0/1	0/1	
**Nucleo-cytoplasmic transport**	0/7	0/7	
**Energy production**	0/4	0/4	
**Purine biosynthesis**	0/1	0/1	
**UDP-glucuronosyl transferase**	0/2	0/2	
**Homo-cystinuria**	0/2	0/2	
**Transcription factor**	0/13	0/13	
**Translation initiation**	0/3	0/3	
**mRNA splicing**	0/4	0/4	
**RNA processing and modification**	0/4	0/4	
**mRNA catabolism**	0/4	0/4	
**Gene expression - other**	0/2	0/2	
**Nucleogenesis**	0/1	0/1	
**Histone**	0/1	0/1	
**Amino acid transport**	0/1	0/1	
**Lipid + fatty acid transport**	0/4	0/4	
**Transporters – other**	0/2	0/2	
**Calcium transport**	0/3	0/3	
**Ion transport**	0/5	0/5	
**Tyr kinase**	0/1	0/1	
**Ser/Thr phosphatase**	0/7	0/7	
**Signal transduction – other**	0/2	0/2	
**Axon guidance**	0/1	0/1	
**Cell division & cycle**	0/6	0/6	
**Meiosis**	0/1	0/1	
**Apoptosis**	0/1	0/1	
**Mitochondrial function**	0/2	0/2	

Figure S2. Comparison between genes differentially regulated upon neuronal *xbp-1s* expression and XBP-1 targets previously identified by microarray. Related to Figure 1.

A comparison of genes found to be differentially regulated in an XBP-1-dependent manner upon UPR^{ER} induction by tunicamycin (INDUCIBLE) or constitutively (CONSTITUTIVE) using microarray analysis (Shen et al., 2005), with genes differentially regulated in neurons upon neuronal *xbp-1s* expression in our RNA-seq analysis.

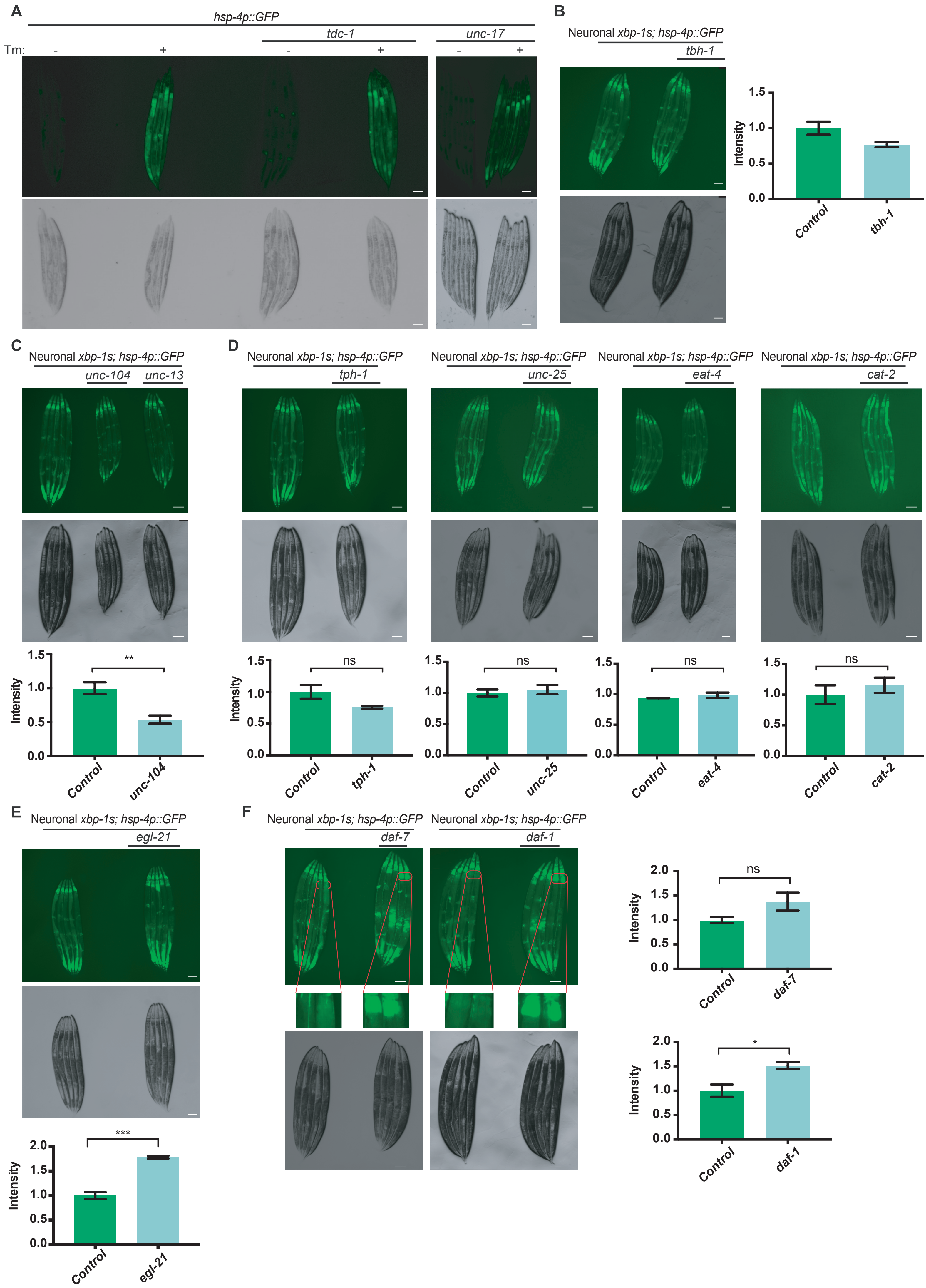

A
hsp-4p::GFP
tdc-1
unc-17
Tm:
-
+
B
Neuronal xbp-1s; hsp-4p::GFP
tbh-1
Intensity
Control
tbh-1
C
Neuronal xbp-1s; hsp-4p::GFP
unc-104
unc-13
**
Control
unc-104
D
Neuronal xbp-1s; hsp-4p::GFP
tph-1
unc-25
eat-4
cat-2
ns
E
Neuronal xbp-1s; hsp-4p::GFP
egl-21
***
F
Neuronal xbp-1s; hsp-4p::GFP
daf-7
daf-1
*

Figure S3. The effects of mutations in neurotransmitter-associated genes on UPR^{ER} activation. Related to Figure 2.

(A) Fluorescence microscopy of *rab-3p::xbp-1s; hsp-4p::GFP*, *unc-17(e245); rab-3p::xbp-1s; hsp-4p::GFP*, and *tdc-1(n3419); rab-3p::xbp-1s; hsp-4p::GFP* animals treated with 2% DMSO or 25 mM tunicamycin for 4 hours. N=3 biological replicates. Scalebars = 100 μm.

(B) Fluorescence microscopy of an additional CRISPR-derived *tbh-1* mutant allele, *tbh-1(rms8)*, in the *rab-3p::xbp-1s; hsp-4p::GFP* genetic background. GFP levels were quantified using ImageJ and normalized to control *rab-3p::xbp-1s; hsp-4p::GFP* animals. N=3 biological replicates each containing ≥5 animals, error bars indicate SEM. Scalebars = 100 μm.

(C) Fluorescence microscopy of *unc-104(e1265)* and *unc-13(e450)* mutant alleles in the *rab-3p::xbp-1s; hsp-4p::GFP* genetic background. GFP levels were quantified using ImageJ and normalized to control *rab-3p::xbp-1s; hsp-4p::GFP* animals, and significance assessed relative to control worms by unpaired student's t-test, **p≤0.01. N≥3 biological replicates each containing ≥5 animals, error bars indicate SEM. Scalebars = 100 μm.

(D) Fluorescence microscopy of *tph-1(mg280), unc-25(e156), eat-4(rms5)* and *cat-2(e1112)* mutant alleles in the *rab-3p::xbp-1s; hsp-4p::GFP* genetic background. GFP levels were quantified using ImageJ and normalized to control *rab-3p::xbp-1s; hsp-4p::GFP* animals, and significance assessed relative to control worms by unpaired student's t-test, ns=not significant. N≥3 biological replicates each containing ≥5 animals, error bars indicate SEM. Scalebars = 100 μm.

(E) Fluorescence microscopy of *egl-21(n611)* mutant allele in the *rab-3p::xbp-1s; hsp-4p::GFP* genetic background. GFP levels were quantified using ImageJ and

normalized to control *rab-3p::xbp-1s; hsp-4p::GFP* animals, and significance assessed relative to control worms by unpaired student's t-test, ***p≤0.001. N≥3 biological replicates each containing ≥5 animals, error bars indicate SEM. Scalebars = 100 μm.

(F) Fluorescence microscopy of *daf-7(e1372)* and *daf-1(m40)* mutant alleles in the *rab-3p::xbp-1s; hsp-4p::GFP* genetic background. GFP levels were quantified using ImageJ and normalized to control *rab-3p::xbp-1s; hsp-4p::GFP* animals, and significance assessed relative to control worms by unpaired student's t-test, *p≤0.05, ns=not significant. N≥3 biological replicates each containing ≥5 animals, error bars indicate SEM. Scalebars = 100 μm.

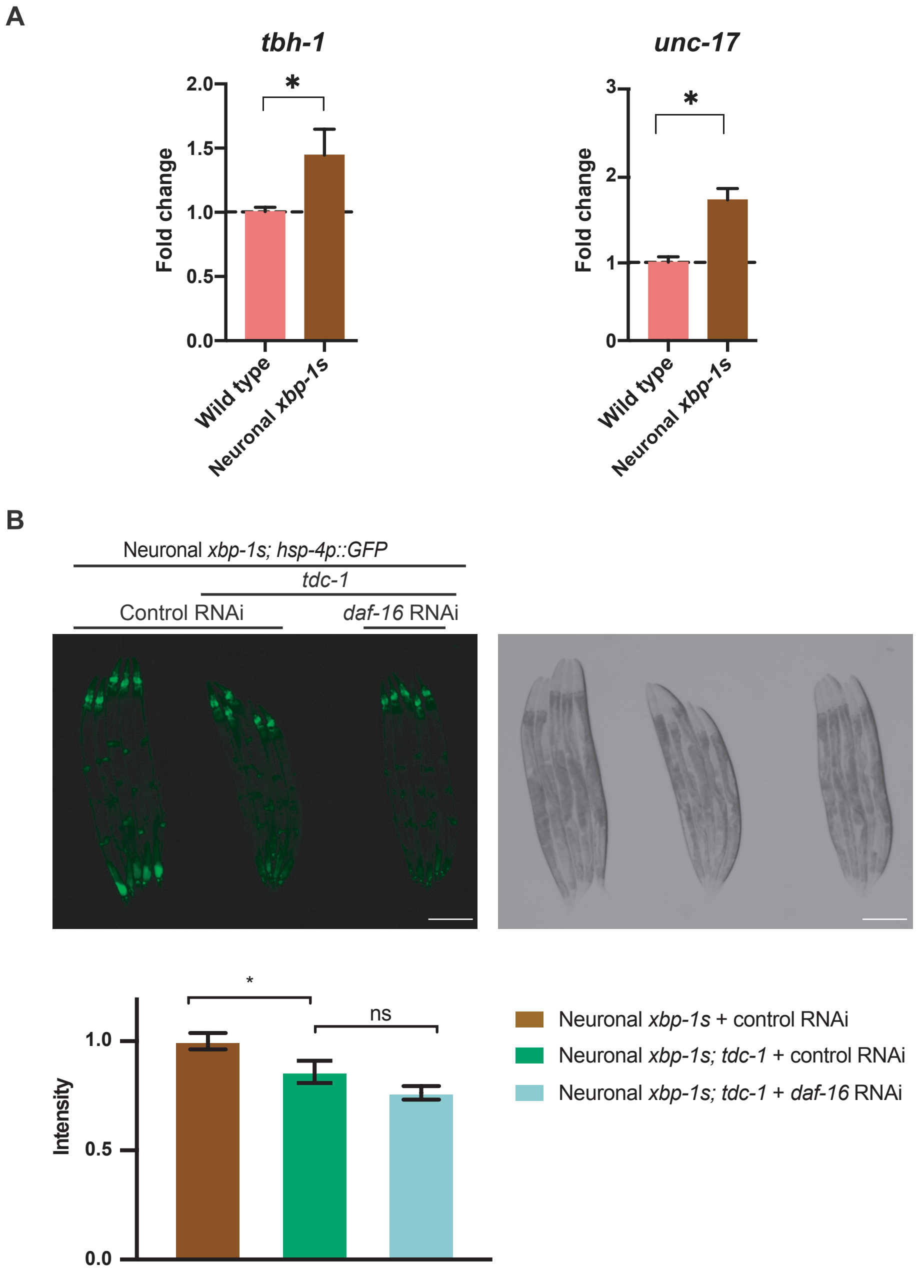

A
tbh-1
unc-17
Fold change
2.0
1.5
1.0
0.5
0.0
3
2
1
0
*
Wild type
Neuronal xbp-1s
B
Neuronal xbp-1s; hsp-4p::GFP
tdc-1
Control RNAi
daf-16 RNAi
Intensity
ns
Neuronal xbp-1s + control RNAi
Neuronal xbp-1s; tdc-1 + control RNAi
Neuronal xbp-1s; tdc-1 + daf-16 RNAi

Figure S4. Neuronal *xbp-1s* upregulates *tbh-1* and *unc-17*, and its effects on intestinal UPR^{ER} activation do not depend on *daf-16*. Related to Figure 2.

(A) RT-qPCR analysis of *tbh-1* and *unc-17* expression in whole worm samples from wild type or *rab-3p::xbp-1s* animals. Fold change was calculated for each transcript relative to wild type, and error bars indicate SEM (N=4 biological replicates). Significance of the difference between levels of each transcript in wild type and neuronal *xbp-1s*-expressing animals was assessed by one-way ANOVA with Tukey's multiple comparisons test, $*p \leq 0.05$.

(B) Fluorescence microscopy of *rab-3p::xbp-1s; hsp-4p::GFP* and *rab-3p::xbp-1s; hsp-4p::GFP; tdc-1(n3419)* animals treated with control or *daf-16* RNAi from hatch. GFP levels were quantified using ImageJ and normalized to control *rab-3p::xbp-1s; hsp-4p::GFP* animals, and significance assessed relative to control worms by unpaired student's t-test, $*p \leq 0.05$, ns=not significant. N=2 biological replicates each containing ≥5 animals, error bars indicate SEM. Scalebars = 250 μm.

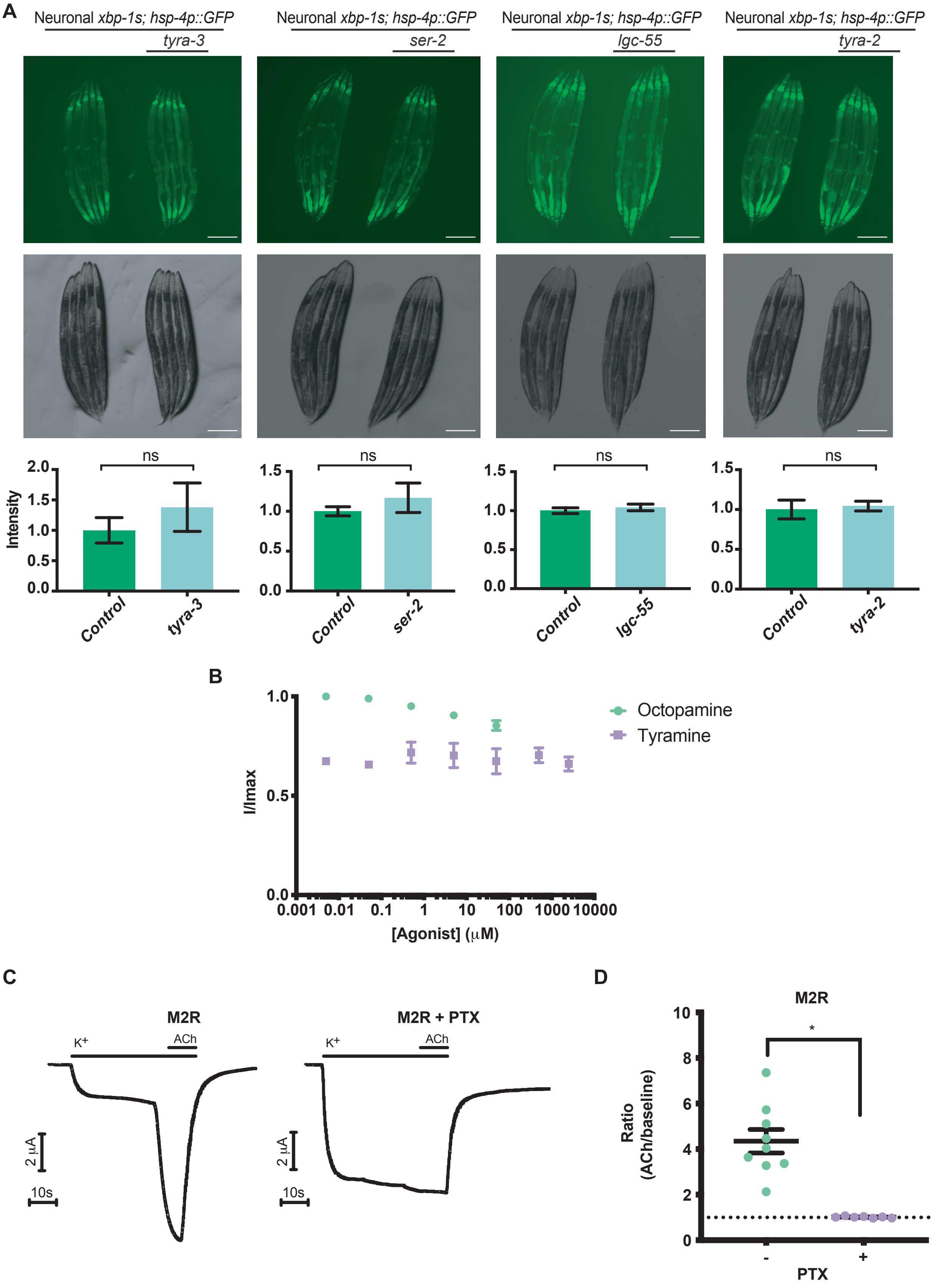

A
Neuronal xbp-1s; hsp-4p::GFP
tyra-3
ser-2
lgc-55
tyra-2
ns
Intensity
Control
B
Octopamine
Tyramine
I/Imax
[Agonist] (μM)
C
M2R
M2R + PTX
K+
ACh
2 μA
10s
D
M2R
Ratio (ACh/baseline)
*
PTX

Figure S5. Analysis of candidate tyramine and octopamine receptors in *rab-3p::xbp-1s; hsp-4p::GFP* animals. Related to Figure 3.

(A) Fluorescence microscopy of *tyra-3(rms1), ser-2(rms3), lgc-55(rms7)* and *tyra-2(rms6)* mutant alleles in the *rab-3p::xbp-1s; hsp-4p::GFP* genetic background. GFP levels were quantified using ImageJ and normalized to control *rab-3p::xbp-1s; hsp-4p::GFP* animals, and significance assessed relative to control worms by unpaired student's t-test, ns=not significant. N≥3 biological replicates each containing ≥5 animals, error bars indicate SEM. Scalebars = 250 μm.

(B) Octopamine and tyramine failed to induce a response in *Xenopus laevis* oocytes expressing only mGIRK1 and mGIRK2. Error bars represent SEM of 3 oocytes. EC_{50} was calculated by fitting data to the Hill equation using a four-parameter variable slope.

(C) Continuous two-electrode voltage clamp (TEVC) recordings from untreated or PTX-injected *Xenopus laevis* oocytes expressing M2R, mGIRK1 and mGIRK2 and treated with 100nM acetylcholine (Ach).

(D) Current ratios of acetylcholine-evoked vs basal high K^+ current for at least 7 oocytes expressing M2R, mGIRK1 and mGIRK2 injected with or without PTX. Error bars represent mean ± SEM. Significance was assessed by unpaired student's t-test, *p≤0.05.

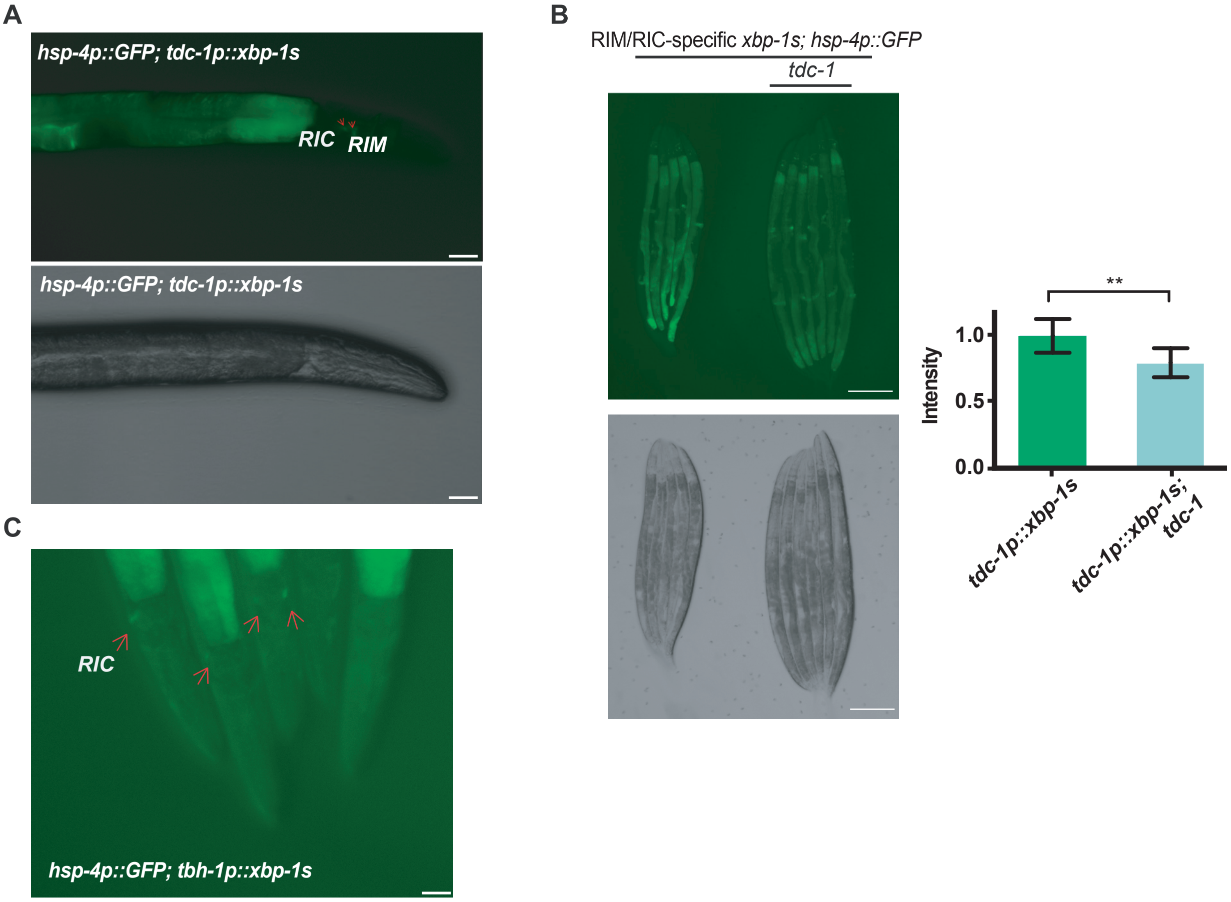

A
hsp-4p::GFP; tdc-1p::xbp-1s
RIC
RIM
hsp-4p::GFP; tdc-1p::xbp-1s
B
RIM/RIC-specific xbp-1s; hsp-4p::GFP
tdc-1
**
Intensity
1.0
0.5
0.0
tdc-1p::xbp-1s
tdc-1p::xbp-1s; tdc-1
C
RIC
hsp-4p::GFP; tbh-1p::xbp-1s

Figure S6. Validation of the functional expression of *xbp-1s* in RIC and RIM interneurons. Related to Figure 4.

(A) Fluorescence microscopy of a *tdc-1p::xbp-1s; hsp-4p::GFP* animal showing GFP fluorescence in the RIC and RIM interneurons. Arrows indicate the RIC and RIM neurons. Scalebars = 20 μm.

(B) Fluorescence microscopy of *tdc-1p::xbp-1s; hsp-4p::GFP* animals with and without a *tdc-1(n3419)* mutation. GFP levels were quantified using ImageJ and normalized to *tdc-1p::xbp-1s; hsp-4p::GFP* animals, and significance assessed relative to control worms by unpaired student's t-test, $^{**}p \leq 0.01$. N=3 biological replicates each containing ≥5 animals, error bars indicate SEM. Scalebars = 250 μm.

(C) Fluorescence microscopy of a *tbh-1p::xbp-1s; hsp-4p::GFP* animal showing GFP fluorescence in the RIC interneurons. Arrows indicate the RIC neurons. Scalebars = 20 μm.

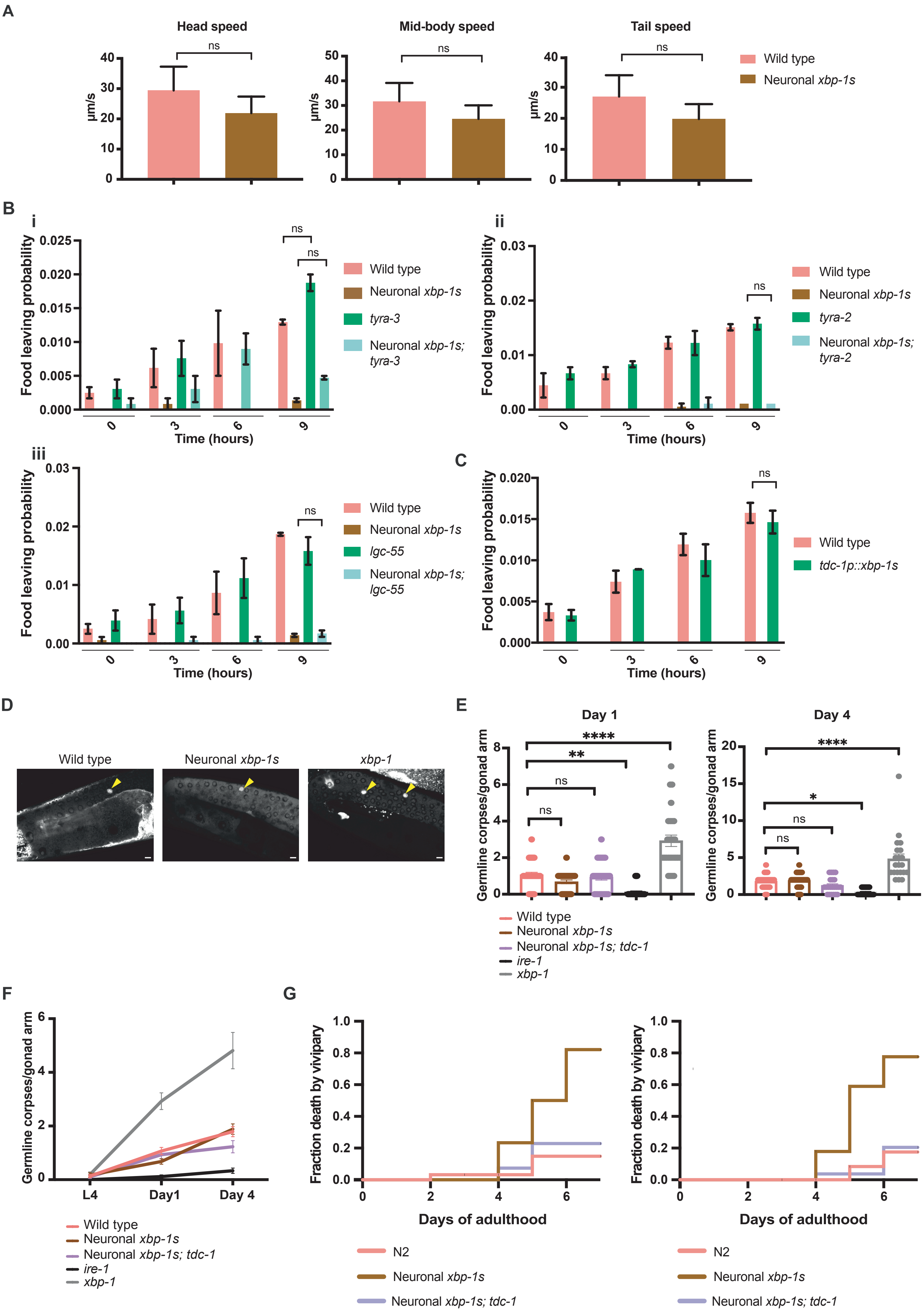

A
Head speed
Mid-body speed
Tail speed
ns
µm/s
Wild type
Neuronal xbp-1s
B
i
ii
iii
Food leaving probability
Time (hours)
Wild type
Neuronal xbp-1s
tyra-3
Neuronal xbp-1s; tyra-3
tyra-2
Neuronal xbp-1s; tyra-2
lgc-55
Neuronal xbp-1s; lgc-55
C
tdc-1p::xbp-1s
D
Wild type
Neuronal xbp-1s
xbp-1
E
Day 1
Day 4
Germline corpses/gonad arm
****
**
*
Neuronal xbp-1s; tdc-1
ire-1
F
L4
Day1
Day 4
G
Fraction death by vivipary
Days of adulthood
N2

Figure S7. *rab-3p::xbp-1s* worms are normally motile, mutations in candidate tyramine and octopamine receptors do not affect their food leaving propensity, and these animals do not display increased levels of germline apoptosis. Related to Figures 5 and 6.

(A) Head speed, mid-body speed and tail speed of *rab-3p::xbp-1s* compared to N2 worms was measured using a WormTracker. Graphs represent average speed in ~20 worms, and error bars indicate SEM. Significance was assessed by unpaired student's t-test, ns=not significant.

(B) Food leaving probability was calculated in (i) *tyra-3(rms1),* (ii) *tyra-2(rms6)* and (iii) *lgc-55(rms7)* animals with and without *rab-3p::xbp-1s* expression, alongside N2 and *rab-3p::xbp-1s* animals. 20 adult worms per strain were placed on 35 mm plates seeded with a drop of OP50 and video-recorded for 15 minutes at t=0, 3, 6, and 9 hours to assess probability of food leaving. Mean probability was then plotted, with error bars representing SEM (N≥3). Significance was assessed as indicated at the final timepoint by two-way ANOVA with Tukey's multiple comparison test, ns=not significant.

(C) Food leaving probability was calculated and plotted as described above in *tdc-1p::xbp-1s* and N2 animals. Error bars represent SEM (N≥3), and significance was assessed between the two strains at the final timepoint by two-way ANOVA with Sidak's multiple comparison test, ns=not significant.

(D) Apoptotic cell corpses in the germlines of N2, *rab-3p::xbp-1s* and *xbp-1(tm2457)* animals were stained with SYTO12 and imaged by confocal microscopy. Images show animals at day 1 of adulthood, arrows indicate apoptotic corpses. Scalebars = 5 μm.

(E) Apoptotic corpses in the germline of N2, *rab-3p::xbp-1s*, *rab-3p::xbp-1s;tdc-1(n3419)*, *ire-1(v33)* and *xbp-1(tm2457)* animals stained with SYTO12 and imaged as above were quantified and the average number of corpses per gonadal arm at day 1 and day 4 of adulthood plotted (N=3, 6-10 worms per strain in each replicate). Significance was calculated by one-way ANOVA with Tukey's multiple comparison test, *p<0.05, **p<0.01, ****p<0.0001, ns=not significant.

(F) Apoptotic corpses in the germline of N2, *rab-3p::xbp-1s*, *rab-3p::xbp-1s;tdc-1(n3419)*, *ire-1(v33)* and *xbp-1(tm2457)* animals stained with SYTO12 and imaged as above were quantified and the average number of corpses per gonadal arm plotted against age, from the L4 larval stage to day 4 of adulthood. Error bars indicate SEM (N=3, 6-10 worms per strain in each replicate).

(G) The number of N2, *rab-3p::xbp-1s*, *tdc-1(n3419)*; *rab-3p::xbp-1s* and *tbh-1(rms2); rab-3p::xbp-1s* animals in which internal hatching was observed was counted for the first 6 days of adulthood and the fraction undergoing internal hatching plotted, in 2 additional biological replicates. Significance was calculated by Mantel-Cox log-rank test; p<0.0001 between *rab-3p::xbp-1s* and N2, and between *rab-3p::xbp-1s* and *rab-3p::xbp-1s*, *tdc-1(n3419)* in each case.
