# Supplementary material for: Tyramine Acts Downstream of Neuronal XBP-1s to Coordinate Inter-tissue UPRER Activation and Behavior in C. elegans
Source: Dev Cell. 2020 Dec 21;55(6):754–770.e6. doi: 10.1016/j.devcel.2020.10.024 (PMC7758879; doi:10.1016/j.devcel.2020.10.024)
Supplement: Document S2. Article plus Supplemental Information [file mmc6.pdf]

Developmental Cell

Article

Tyramine Acts Downstream of Neuronal XBP-1s to Coordinate Inter-tissue UPR^{ER} Activation and Behavior in *C. elegans*

Graphical Abstract

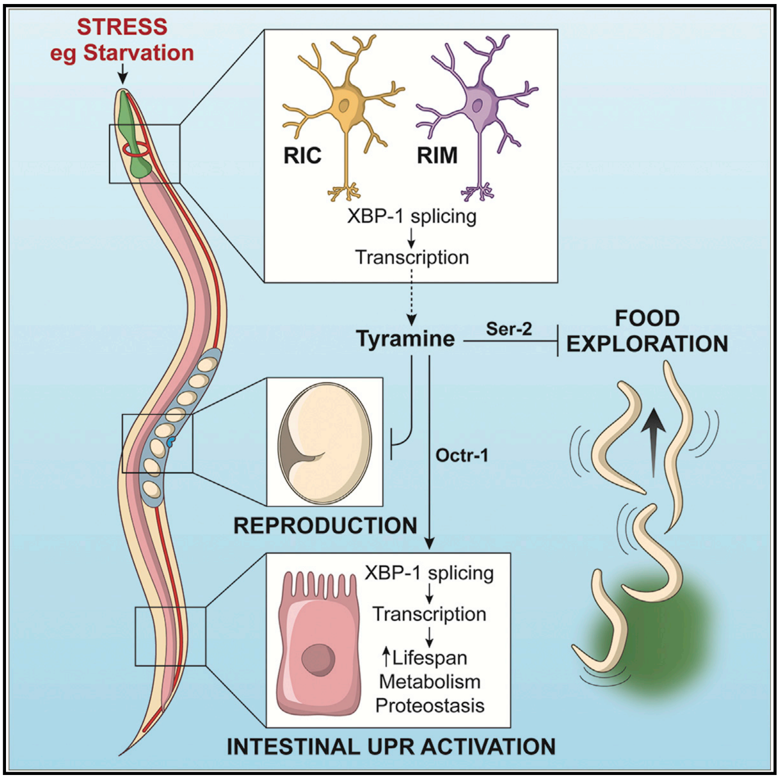

Authors

Neşem P. Özbey, Soudabeh Imanikia, Christel Krueger, ..., William R. Schafer, M. Olivia Casanueva, Rebecca C. Taylor

Correspondence

rtaylor@mrc-lmb.cam.ac.uk

In Brief

Neuronal XBP-1s expression drives intestinal UPRER activation in *C. elegans*. Özbey et al. define the transcriptional targets of neuronal XBP-1s and find that tyramine and the RIM/RIC interneurons are required for distal UPR activation. Neuronal XBP-1s also modulates behavior and reproduction in a tyramine-dependent manner, globally coordinating organismal responses to stress.

Highlights

- XBP-1s drives a distinct transcriptional program in *C. elegans* neurons
- Neuronal XBP-1s requires tyramine synthesis to induce intestinal UPR activation
- XBP-1s expression in RIM and RIC neurons drives cell non-autonomous UPR activation
- Neuronal XBP-1s changes behavior and reproduction, dependent on tyramine synthesis

Özbey et al., 2020, Developmental Cell *55*, 754–770
December 21, 2020 © 2020 MRC Laboratory of Molecular Biology.
Published by Elsevier Inc.
https://doi.org/10.1016/j.devcel.2020.10.024

CellPress
OPEN ACCESS

Developmental Cell

Article

Tyramine Acts Downstream of Neuronal XBP-1s to Coordinate Inter-tissue UPR^{ER} Activation and Behavior in *C. elegans*

Neşem P. Özbey,[1] Soudabeh Imanikia,[1] Christel Krueger,[2] Iris Hardege,[1] Julia Morud,[1] Ming Sheng,[1] William R. Schafer,[1] M. Olivia Casanueva,[2] and Rebecca C. Taylor[1,3,*]
[1]Neurobiology Division, MRC Laboratory of Molecular Biology, Cambridge CB2 0QH, UK
[2]Epigenetics Programme, The Babraham Institute, Babraham CB22 3AT, UK
[3]Lead Contact
*Correspondence: rtaylor@mrc-lmb.cam.ac.uk
https://doi.org/10.1016/j.devcel.2020.10.024

SUMMARY

In *C. elegans*, expression of the UPR^{ER} transcription factor *xbp-1s* in neurons cell non-autonomously activates the UPR^{ER} in the intestine, leading to enhanced proteostasis and lifespan. To better understand this signaling pathway, we isolated neurons from animals expressing neuronal *xbp-1s* for transcriptomic analysis, revealing a striking remodeling of transcripts involved in neuronal signaling. We then identified signaling molecules required for cell non-autonomous intestinal UPR^{ER} activation, including the biogenic amine tyramine. Expression of *xbp-1s* in just two pairs of neurons that synthesize tyramine, the RIM and RIC interneurons, induced intestinal UPR^{ER} activation and extended longevity, and exposure to stress led to splicing and activation of *xbp-1* in these neurons. In addition, we found that neuronal *xbp-1s* modulates feeding behavior and reproduction, dependent upon tyramine synthesis. XBP-1s therefore remodels neuronal signaling to coordinately modulate intestinal physiology and stress-responsive behavior, functioning as a global regulator of organismal responses to stress.

INTRODUCTION

The ability to respond appropriately to stressful environments is crucial to the survival of organisms in the face of changing conditions. In cells, the ability to adapt molecular functions in response to the damage caused by stress-inducing agents, such as heat, pathogens, or xenobiotics, is mediated by cellular stress responses. These signaling pathways link the presence of damage or disequilibrium within different cellular compartments to the activation of molecular mechanisms that restore homeostasis. One example is the unfolded protein response of the endoplasmic reticulum (UPR^{ER}), which monitors homeostasis within the secretory pathway through three upstream stress-detecting molecules positioned at the ER membrane—IRE1, PERK, and ATF6. These sensors are activated by homeostatic imbalance in the ER and regulate downstream signaling pathways with both transcriptional and translational outputs to re-establish equilibrium (Walter and Ron, 2011).

The most evolutionarily ancient of these pathways is regulated by IRE1, a conserved kinase/endoribonuclease that functions in part through the regulated splicing of a downstream leucine zipper transcription factor, XBP1 (Calfon et al., 2002; Cox and Walter, 1996). Spliced and active XBP1 then transcriptionally regulates downstream target genes that contribute to the restoration of homeostasis in stressed cells. As well as mediating responses to environmental stress, XBP1 also plays important roles in cellular differentiation, development, and metabolism and has a broad range of transcriptional target genes (Acosta-Alvear et al., 2007; Shen et al., 2005). In *C. elegans*, in addition to its roles in stress resistance at the cellular level, XBP-1 also orchestrates inter-tissue coordination of the ER stress response (Taylor and Dillin, 2013). This involves a neuronal signaling mechanism, activated when spliced XBP-1s is expressed in neurons, that leads to downstream UPR^{ER} activation in intestinal cells, and striking increases in organismal stress resistance and longevity. This signaling mechanism involves the release of synaptic vesicles, dependent upon the neuronal exocytosis regulator UNC-13. However, the identity of the signaling molecule(s) mediating this communication of UPR^{ER} activation between neurons and the intestine has been elusive.

These findings suggest that XBP-1s may have distinct tissue-specific functions in the worm, including the ability to trigger neuronal signaling events when expressed in neurons and to extend longevity when activated in the intestine. One explanation is that XBP-1s may regulate tissue-specific sets of target genes. However, the ability to analyze the transcriptome of specific tissues in adult worms has only recently been achieved, and we therefore have a limited understanding of the degree to which transcription factors regulate different genes in different cell types of this organism (Imanikia et al., 2019a; Kaletsky et al.,

 © 2020 MRC Laboratory of Molecular Biology. Published by Elsevier Inc.
This is an open access article under the CC BY license (http://creativecommons.org/licenses/by/4.0/).

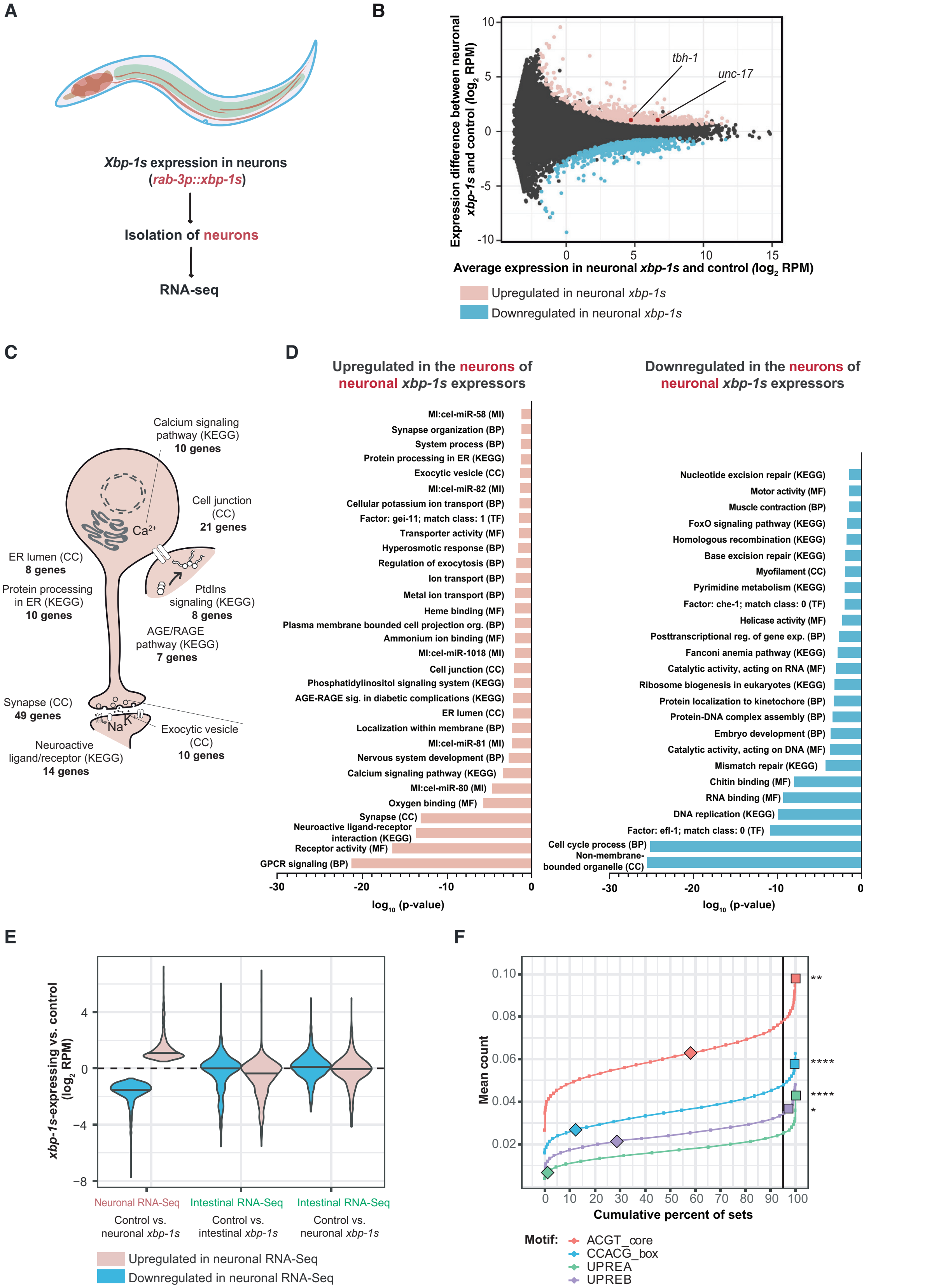

(legend on next page)

2016, 2018). We therefore decided to analyze the neuronal transcriptome of adult *C. elegans* expressing *xbp-1s* in the nervous system to determine whether this transcription factor regulates neuron-specific gene expression that might shed light on the signaling mechanisms leading to inter-tissue UPR^{ER} activation.

We find that *xbp-1s* expression in neurons drives a distinct transcriptional program, with altered expression of many genes involved in neuronal signaling. We then screened candidate signaling pathways for involvement in the transmission of UPR^{ER} activation to the intestine and found that genes mediating acetylcholine signaling and tyramine synthesis are required for inter-tissue UPR^{ER} activation. To our surprise, expression of *xbp-1s* in only the two pairs of neurons that synthesize tyramine, the RIM and RIC interneurons, is sufficient to activate the UPR^{ER} in the intestine and increase longevity, and *xbp-1* is spliced in these neurons upon stress, including nutrient deprivation. We then asked whether this altered neuronal signaling landscape gives rise to differential regulation of behavior or reproduction in animals expressing neuronal *xbp-1s*. We find that, indeed, both feeding behavior and brood size are altered in neuronal *xbp-1s*-expressing animals, dependent upon synthesis of tyramine. Interestingly, these behavioral and reproductive alterations resemble those that occur in response to starvation. *C. elegans* may therefore utilize stress-induced neuronal UPR^{ER} activation to trigger parallel tyramine-dependent adaptive responses: feeding and reproductive strategy are altered, to increase survival of individuals and their offspring; and the UPR^{ER} is activated in the intestine, enhancing organismal stress resistance, proteostasis, metabolism, and longevity.

RESULTS

To examine the neuronal transcriptome of animals expressing pan-neuronal *xbp-1s*, we dissociated cells from *C. elegans* expressing pan-neuronal GFP (*unc-119p::GFP*) and *xbp-1s* (*rab-3p::xbp-1s*) (Figure 1A). This enabled the isolation of fluorescent neurons by FACS using an approach similar to that described previously (Figure S1A) (Imanikia et al., 2019a; Kaletsky et al., 2016, 2018). Following RNA extraction, we used RT-qPCR to confirm enrichment of neuronal cell populations (Figure S1B). Interestingly, qPCR analysis also revealed elevated levels of UPR^{ER}—and HSR—, but not UPR^{mt}-associated transcripts in neuronal versus non-neuronal cells of wild-type animals (Figure S1C). We ensured that the sorting process was not itself activating stress responses, as cells that had been sorted and then recombined did not consistently exhibit higher levels of stress response transcripts than unsorted cells (Figure S1D). The transcriptome of neurons from neuronal *xbp-1s* expressing compared with control animals was then analyzed by RNA-seq. We again confirmed enrichment of neuronal transcripts in our samples (Figure S1E) and identified rare transcripts expressed only in highly restricted sets of neurons, demonstrating high sensitivity and neuronal coverage (Figure S1F).

Gene Regulation by XBP-1s Is Highly Tissue Specific

Our analysis revealed that expression of *xbp-1s* in neurons leads to differential regulation of a substantial number of genes in neuronal cells (Figure 1B; Data S1). GO analysis of the transcripts altered in neurons by neuronal *xbp-1s* showed upregulation of known UPR^{ER} targets, defined by GO terms "protein processing in ER" and "ER lumen" (Figures 1C–1D). However, the majority of differentially regulated GO terms represented neuron-specific functions and processes—for example, the function and organization of the synapse, GPCR signaling, and nervous system development. While some GO terms overlap with those we previously found to be differentially regulated by *xbp-1s* activation in the intestine, the majority differed; for example, the upregulation of genes involved in lysosome function that we observed in intestinal cells was not seen in the neuronal *xbp-1s* transcriptome (Imanikia et al., 2019a). Overall, the genes that were differentially regulated by *xbp-1s* in neurons were not significantly altered in the intestinal transcriptome of *xbp-1s*-expressing animals, relative to wild type (Figure 1E). This suggests that *xbp-1s* regulates mostly distinct, tissue-specific target gene sets in different cell types, facilitating the regulation of tissue-specific functions.

We also compared the genes that were differentially regulated in neurons by neuronal *xbp-1s* expression with both tunicamycin-inducible and constitutive UPR^{ER} genes found to depend on the presence of XBP-1 in whole animal microarray analysis (Shen et al., 2005). We found that, of 139 genes of known function shown to depend on XBP-1 for induction after tunicamycin treatment, 31 were upregulated in our analysis, while none were downregulated (Figure S2). In contrast, among 184 genes shown to represent the constitutive XBP-1-dependent UPR^{ER}, 15 were upregulated in our analysis, and 5 were downregulated. The overlap between our findings and the inducible UPR^{ER} gene set suggests that our system may be more representative of a stress scenario, in which the UPR^{ER} is acutely activated, than an increased capacity of the constitutive UPR^{ER}. The transcripts that appear in both the inducible UPR^{ER} and neuronal *xbp-1s*

Figure 1. XBP-1s Differentially Regulates Neuron-Specific Genes and Processes in the *C. elegans* Nervous System

(A) Schematic showing the experimental setup for neuronal transcriptomics.

(B) MA plot showing transcripts in isolated neuronal cells with and without neuronal *xbp-1s* expression. Differentially regulated transcripts are highlighted in pink (upregulated) or blue (downregulated); the upregulated genes *tbh-1* and *unc-17* are annotated. RPM = reads per million mapped reads.

(C) Upregulated GO terms in neurons from *rab-3p::xbp-1s* animals.

(D) GO terms differentially regulated in neurons from *rab-3p::xbp-1s* versus wild-type animals, ranked by statistical significance. BP, biological process; CC, cellular component; MF, molecular function; Mi, microRNA; KEGG, Kyoto Encyclopedia of Genes and Genomes.

(E) Violin plot showing expression of genes up- or downregulated in neurons of neuronal *xbp-1s*-expressing worms, in neuronal and intestinal RNA-seq datasets. The two intestinal RNA-seq datasets compare the intestinal transcriptome of neuronal and intestinal *xbp-1s*-expressing animals to controls.

(F) Monte Carlo analysis of XBP-1s-binding motifs in promoters of differentially expressed genes. Cumulative percentage of random gene sets is plotted against mean number of motif occurrences per promoter. Mean occurrence of XBP-1s-binding motifs in up- and downregulated gene sets (filled squares and diamonds, respectively) is superimposed onto the distributions. p values denote the proportion of random sets with a higher mean count than the up- or downregulated genes. p = 0.05 significance threshold is indicated, *$p \leq 0.05$ (UPREB), **$p \leq 0.01$ (ACGT_core), ****$p \leq 0.0001$ (CCACG_box, UPREA). See also Figures S1 and S2; Data S1.

CellPress
OPEN ACCESS

lists include many involved in protein folding and membrane/vesicle trafficking, suggesting that these genes may represent a core, all-tissue set of XBP-1s targets.

Finally, to determine whether the genes altered by *xbp-1s* expression in neurons were likely to be direct XBP-1s targets, we searched for the presence of 4 known XBP-1s-binding motifs around the transcriptional start site (−100bp–+100bp) of each gene set compared with a large number of random subsets, using Monte Carlo analysis (Figure 1F). We found that the ACGT core, CCACG box, and UPR elements A and B (Acosta-Alvear et al., 2007; Clauss et al., 1996) were highly significantly overrepresented among the upregulated genes, suggesting that these genes are likely to include direct targets of XBP-1s.

Acetylcholine and Biogenic Amine Signaling Are Required for Cell Non-autonomous UPR^{ER} Activation

Given the profoundly altered neuronal signaling landscape upon *xbp-1s* expression in neurons, we hypothesized that altered neurotransmitter signaling pathways downstream of *xbp-1s* expression might lead to the release of signaling molecules that activate the UPR^{ER} in intestinal cells (Figure 2A). To determine which neurotransmitters are involved, we introduced loss-of-function mutations in a range of molecules involved in synthesis and release of specific neurotransmitters into animals expressing neuronal *xbp-1s* and a marker for UPR^{ER} activation (*hsp-4p::GFP*) (Figure 2B). We found that mutation of *unc-17*, involved in the packaging of acetylcholine into vesicles, and a deletion mutation in *tdc-1*, which encodes the tyrosine decarboxylase that synthesizes tyramine, caused statistically significant suppression of intestinal UPR^{ER} activation (Figures 2B–2C). In contrast, neither *tdc-1* nor *unc-17* mutations inhibited the ability of animals to activate the UPR^{ER} in the intestine upon treatment with the ER stress-inducing drug tunicamycin (Figure S3A).

We also examined a mutation in *tbh-1*, the enzyme responsible for the synthesis of the biogenic amine octopamine from tyramine. As we were unable to cross existing *tbh-1* mutations into *rab-3p::xbp-1s* animals, we created a deletion allele, *tbh-1(rms2)*, using CRISPR. This 1090-bp deletion encompasses the deletion in the *tbh-1(n3247)* loss-of-function allele, spanning exons 6, 7, and 8, and introduces a premature stop codon that truncates the protein. This *tbh-1* allele also somewhat reduced intestinal UPR^{ER} activation, but the difference was not statistically significant (Figures 2B–2C and S3B). In addition, mutation of *unc-104*, encoding a synaptic motor protein, suppressed intestinal UPR^{ER} activation, confirming that synaptic vesicle release is necessary for this process (Figures 2B and S3C). In contrast, mutations in genes involved in dopamine, serotonin, GABA, and glutamate synthesis had no effect on intestinal UPR^{ER} activation (Figures 2B and S3D), while mutations in *egl-21*, a carboxypeptidase involved in secretion, and in the TGFβ ligand and receptor homologs *daf-7* and *daf-1* increased intestinal UPR^{ER} activation (Figures 2B, S3E, and S3F).

Interestingly, levels of *unc-17* and *tbh-1* transcripts were increased in our transcriptomic analysis of neuronal *xbp-1s* animals, suggesting that regulation of acetylcholine and tyramine/octopamine signaling pathways might be altered upon *xbp-1s* expression (Figures 1B, 2D, and S4A). We hypothesized that release of acetylcholine and synthesis of tyramine might play roles in the transmission of UPR^{ER} activation between neurons and the intestine. Confirming this, mutations in *unc-17* and *tdc-1* significantly suppressed the lifespan extension associated with neuronal *xbp-1s* expression, which depends upon the downstream activation of the UPR^{ER} in intestinal cells (Figure 2E; Table S1) (Taylor and Dillin, 2013). We were particularly intrigued by the role that *tdc-1* might play in this process because biogenic amines have previously been implicated in inter-tissue signaling and stress response regulation (Burkewitz et al., 2015; De Rosa et al., 2019; Tao et al., 2016). We therefore asked whether loss of tyramine altered other downstream effects of neuronal *xbp-1s* expression. Indeed, we found that mutation of *tdc-1* abolished the ability of neuronal *xbp-1s* to upregulate the transcription of lysosomal genes previously shown to be induced in the intestine following neuronal UPR^{ER} activation, although another proposed target gene, *fat-6*, was still upregulated by neuronal *xbp-1s* in *tdc-1* mutants (Figure 3A) (Imanikia et al., 2019a, 2019b). In addition, when combined with expression of intestinal polyglutamine expansions, neuronal *xbp-1s* leads to lower levels of toxic protein species; in the presence of a *tdc-1* mutation, levels of polyQ:YFP are partially restored (Figure 3B) (Imanikia et al., 2019a). This demonstrates that inhibiting synthesis of tyramine suppresses multiple downstream effects of cell non-autonomous UPR^{ER} activation, including extension of longevity, regulation of lysosomal genes, and enhanced proteostasis.

As tyramine has been linked to reduced insulin/IGF1-like signaling (IIS) in *C. elegans* and reduced IIS lowers *hsp-4p::GFP* activity, we asked whether knockdown of the IIS transcription factor *daf-16* suppressed loss of UPR activation in *tdc-1* mutants; however, this was not the case (Figure S4B) (De Rosa et al., 2019; Henis-Korenblit et al., 2010). In addition, deletion mutations in four proposed tyramine receptors—*tyra-3, ser-2*, *lgc-55*, and *tyra-2*—also failed to suppress intestinal UPR^{ER} activation (Figure S5A). However, when we examined the transcriptome of intestinal cells from animals expressing *xbp-1s* in neurons or the intestine to determine whether expression levels of any candidate receptors in the intestine were altered by UPR^{ER} activation, we found that the expression of one known biogenic amine receptor, *octr-1*, was reduced upon *xbp-1s* expression (Figure 3C) (Imanikia et al., 2019a). This interested us, as reduced levels of *octr-1* have been implicated in activation of the UPR^{ER} in *C. elegans*, although the receptor was previously thought to be expressed only in neurons (Sun et al., 2012, 2011).

Intrigued by the possibility that intestinal OCTR-1 might be involved in the regulation of UPR^{ER} activation in this tissue, we asked whether reduction of *octr-1* levels by intestine-specific RNAi in animals expressing the *hsp-4p::GFP* UPR^{ER} reporter was sufficient to increase intestinal UPR^{ER} activation. We found that intestinal *octr-1* knockdown induced a small but significant activation of *hsp-4p::GFP* in the intestine, suggesting that OCTR-1 can function in this tissue to regulate UPR^{ER} activity (Figure 3D). As OCTR-1 is thought to be an octopamine-specific receptor, we then asked whether OCTR-1 can also bind other neurotransmitters, using a *Xenopus laevis* oocyte expression system coupled with two-electrode voltage clamp (TEVC) recording. We found that OCTR-1 responds to tyramine treatment in this system, although with a higher EC_{50} than recorded for octopamine; other neurotransmitters elicited no response

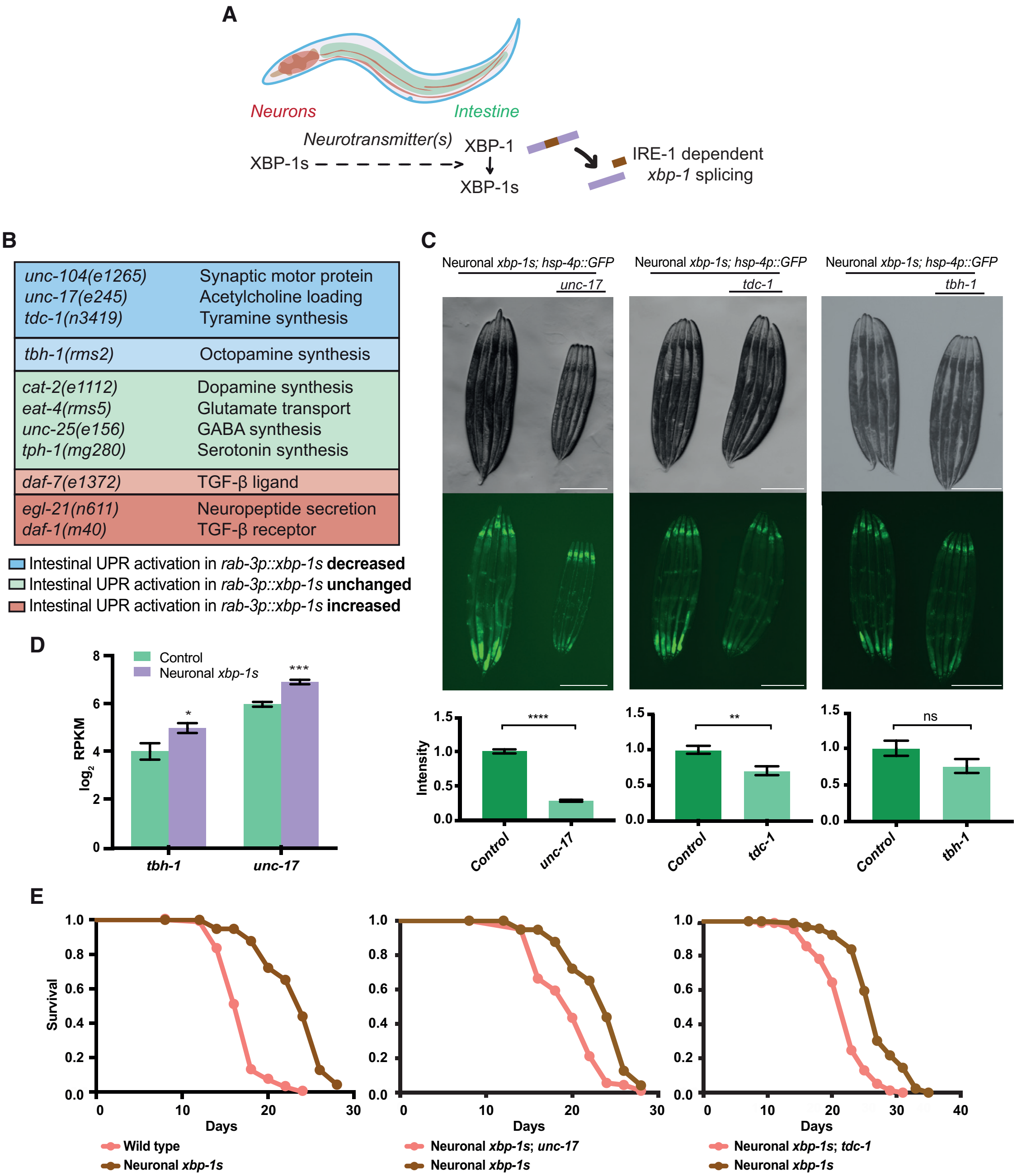

Figure 2. Identification of Neurotransmitter-Related Genes that Modulate Cell Non-autonomous UPR^{ER} Activation

(A) Schematic showing the proposed mechanism by which neuronal *xbp-1s* induces intestinal UPR^{ER} activation.

(B) Mutations assessed for their effect on intestinal UPR^{ER} activation in *rab-3p::xbp-1s; hsp-4p::GFP* animals.

(C) Fluorescence microscopy of *unc-17(e245)*, *tdc-1(n3419)*, and *tbh-1(rms2)* mutant alleles in *rab-3p::xbp-1s; hsp-4p::GFP* animals. GFP levels were quantified using ImageJ and normalized to control *rab-3p::xbp-1s; hsp-4p::GFP* animals; significance was assessed relative to control by unpaired Student's t test, $^{**}p \leq 0.01$, $^{****}p \leq 0.0001$, ns = not significant. N $\geq$ 3 biological replicates with $\geq$5 animals, error bars indicate SEM. Scalebars, 300 µm.

(D) Expression levels of *tbh-1* and *unc-17* in *unc-119p::GFP* control versus *unc-119p::GFP; rab-3p::xbp-1s* animals in neuron-specific RNA-seq analysis. RPKM, reads per kilobase per million mapped reads. Error bars represent SEM from 3 biological replicates. Significance assessed using DeSeq2 with FDR correction, $^{*}p \leq 0.05$, $^{***}p \leq 0.001$.

(E) Lifespan analysis of N2 wild type and *rab-3p::xbp-1s* neuronal *xbp-1s*-expressing (median lifespans 18 versus 24 days, $p < 0.0001$), *rab-3p::xbp-1s* and *unc-17(e245); rab-3p::xbp-1s* (median lifespans 24 versus 20 days, $p < 0.0001$), and *rab-3p::xbp-1s* and *tdc-1(n3419); rab-3p::xbp-1s* (median lifespans 27 versus 23 days, $p<0.0001$) animals. Graphs were plotted as Kaplan-Meier survival curves, and p values calculated by Mantel-Cox log-rank test. N = 80–120 animals per condition in $\geq$3 biological replicates. See also Figures S3 and S4; Table S1.

CellPress
OPEN ACCESS

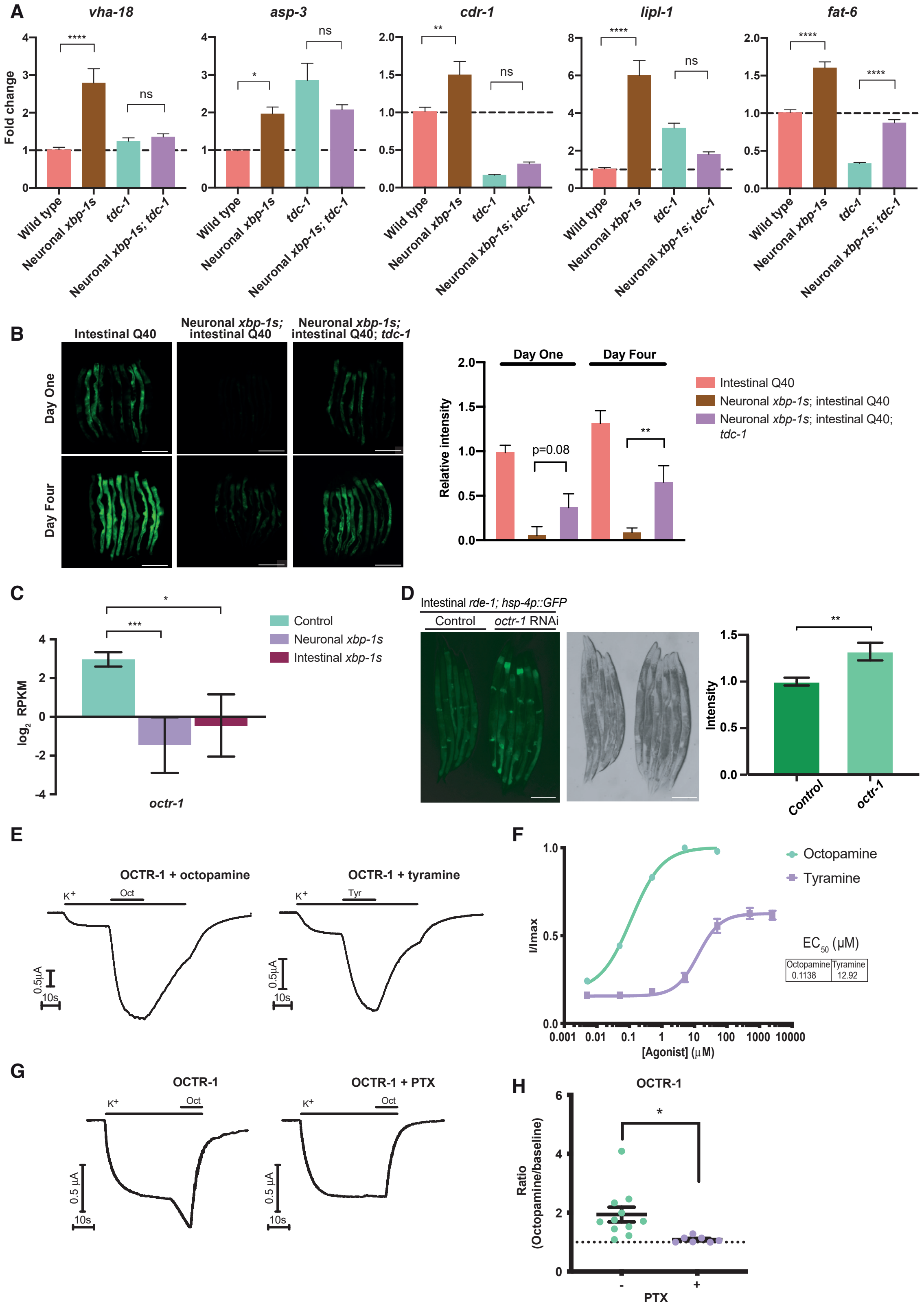

Figure 3. Intestinal Effects of Neuronal *xbp-1s* Activation Require Tyramine

(A) RT-qPCR analysis of *vha-18, asp-3, cdr-1*, *lipl-1*, and *fat-6* in whole worm samples from wild type, *rab-3p::xbp-1s, tdc-1(n3419)*, and *rab-3p::xbp-1s;tdc-1(n3419)* animals. Fold change was calculated relative to wild type; error bars indicate SEM (N = 3 biological replicates). Significance between wild type and

(legend continued on next page)

(Figures 3E–3F and S5B). This suggests that tyramine may also act as an *in vivo* ligand for OCTR-1. In addition, we found that treatment with pertussis toxin (PTX) inhibited OCTR-1 activity, to the same extent as the known $G_{i/o}$-coupled GPCR muscarinic M2 receptor (Figures 3G–3H, S5C, and S5D), suggesting that OCTR-1 is also a $G_{i/o}$-coupled GPCR. While the mechanism is yet to be fully determined, our data raise the intriguing possibility that OCTR-1 is able to respond to tyramine and/or octopamine in the intestine to regulate UPR^{ER} activation in this tissue, with its levels modulated by *xbp-1s* expression.

xbp-1s Expression in RIM and RIC Interneurons Induces Intestinal UPR^{ER} Activation

Expression of *tdc-1* and *tbh-1*, the enzymes responsible for synthesis of tyramine and octopamine, respectively, is limited to only two pairs of neurons; *tbh-1* is expressed only in the RIC interneurons, while *tdc-1* is expressed in the RIM and RIC interneurons (Figure 4A) (Alkema et al., 2005). We therefore asked whether expression of *xbp-1s* in these restricted sets of neurons can activate the UPR^{ER} in the intestine. We first drove *xbp-1s* expression in RIM and RIC, under the *tdc-1* promoter. Importantly, to circumvent inappropriate intestinal expression observed when this promoter was combined with the *unc-54* 3′UTR—also reported for other neuronal promoters (Silva-García et al., 2019) —we utilized the *let-858* 3′UTR. This combination drove highly specific expression in RIM/RIC interneurons, with additional expression seen only in the uterine vulval (UV1) cells, as previously described (Figure 4B) (Alkema et al., 2005). Remarkably, we found that expression of *xbp-1s* in just the RIM and RIC interneurons was sufficient to drive significant UPR^{ER} activation within the intestine (Figures 4C and S6A) and also reduced animal size. Mutation of *tdc-1* decreased UPR^{ER} activity in these animals and rescued their size, suggesting that tyramine is required downstream of *xbp-1* in RIM/RIC neurons to induce UPR^{ER} activation (Figure S6B). In contrast, expression of *xbp-1s* in only the RIC interneurons, driven by the *tbh-1* promoter, had no effect on intestinal UPR^{ER} activation, despite inducing detectable UPR^{ER} activation within the RIC neurons themselves, suggesting that the RIM neurons may play a critical role in driving cell non-autonomous UPR^{ER} activation (Figures 4D and S6C).

Expression of *tdc-1p::xbp-1s* was also sufficient to extend longevity (Figure 4E; Table S1). The degree of both intestinal UPR^{ER} activation and lifespan extension observed in *tdc-1p::xbp-1s* animals was less than that induced by pan-neuronal *xbp-1s* expression, suggesting that additional neuronal mechanisms may be involved. It was striking, however, to observe these effects upon *xbp-1s* expression in such a restricted subset of neurons, which strongly implicates the RIM and RIC interneurons as key mediators of the inter-tissue signaling effects of neuronal *xbp-1s* expression.

Xbp-1 Is Spliced in RIM and RIC Interneurons upon Tunicamycin Treatment or Starvation

To ask whether these neurons play a physiologically relevant role upon exposure to UPR^{ER}-inducing environmental stressors, we monitored splicing of *xbp-1* in the RIM/RIC interneurons when animals were exposed to stress. To achieve this, we created worms expressing a reporter for *xbp-1* splicing, based on a previously described strain (Shim et al., 2004), in combination with a marker for the RIM/RIC neurons (*tdc-1p::mKate*). In these animals, genomic *xbp-1* expressed under the *xbp-1p* promoter is fused with GFP such that GFP is produced in frame only when the regulated intron of *xbp-1* is spliced out by active IRE-1 (Figure 4F). These animals were treated with tunicamycin, which induced robust splicing of the *xbp-1* splicing reporter in a range of neurons, including RIM and RIC (Figures 4G and 4H). Importantly, we found that another stress, starvation, also induced *xbp-1* splicing in neurons, including RIM and RIC (Figures 4G and 4H). In addition, starvation caused significant UPR activation in the intestine (Figures 4I and 4J). This suggests that neuronal *xbp-1s* expression may be a physiologically relevant model for the effects of UPR^{ER}-inducing environment stresses experienced by *C. elegans*, such as the presence of xenobiotics, and low nutrient conditions, that lead to *xbp-1* splicing in neurons.

Neuronal *xbp-1s* Expression Alters Feeding and Exploration Behaviors

Correct interpretation of nutritional status is critical in determining multiple aspects of physiology. Changes in nutrient availability trigger a range of responses in *C. elegans*, including changes to metabolism, stress resistance, longevity, and

rab-3p::xbp-1s, and between *tdc-1(n3419)* and *rab-3p::xbp-1s;tdc-1(n3419)* animals was assessed by one-way ANOVA with Tukey's multiple comparisons test, $^*p \leq 0.05$, $^{**}p \leq 0.01$, $^{****}p \leq 0.0001$, ns = not significant.

(B) Fluorescence microscopy of *vha-6p::Q40::YFP* animals, with and without *rab-3p*::*xbp-1s* and *tdc-1(n3419)* mutation, at day 1 and day 4 of adulthood. YFP fluorescence was quantified using Fiji and normalized to day 1 *vha-6p::Q40::YFP* animals; significance was assessed using one-way ANOVA with Tukey's multiple comparisons test, $^{**}p \leq 0.01$. N = 3 biological replicates containing $\geq$10 animals, error bars indicate SEM. Scalebars, 250 μm.

(C) Expression levels of *octr-1* in intestine-specific RNA-seq analysis of control versus *rab-3p*::*xbp-1s* or *gly-19p::xbp-1s* animals. RPKM, reads per kilobase per million mapped reads. Error bars represent SEM from 3 biological replicates. Significance was assessed by intensity difference filter, $^*p \leq 0.05$, $^{***}p \leq 0.001$.

(D) Fluorescence microscopy of *gly-19p::rde-1; rde-1(rms10); zcls4(hsp-4p::GFP)* animals expressing intestine-specific *rde-1* in an *rde-1(rms10)* mutant for intestine-specific RNAi, grown on empty vector control or *octr-1* RNAi bacteria from hatch and imaged as day 1 adults. GFP levels were quantified using ImageJ and normalized to control *gly-19p::rde-1; rde-1(rms10); zcls4(hsp-4p::GFP)* animals; significance was assessed relative to control by unpaired Student's t test, $^{**}p \leq 0.01$. N = 3 biological replicates containing $\geq$5 animals, error bars indicate SEM. Scalebars, 250 μm.

(E) Continuous TEVC recordings from *Xenopus laevis* oocytes expressing OCTR-1, mGIRK1, and mGIRK2 and treated with 100 nM octopamine (Oct) or 10-μM tyramine (Tyr).

(F) Octopamine- and tyramine-induced dose response curves in *Xenopus laevis* oocytes expressing OCTR-1, mGIRK1, and mGIRK2. Error bars represent SEM of 8 oocytes. EC_{50} was calculated by fitting data to the Hill equation using a four-parameter variable slope.

(G) Continuous TEVC recordings from untreated or PTX-injected *Xenopus laevis* oocytes expressing OCTR-1, mGIRK1, and mGIRK2 and treated with 100 nM octopamine (Oct) or 10-μM tyramine (Tyr).

(H) Current ratios of octopamine-evoked versus basal high K^+ current for at least 7 oocytes expressing OCTR-1, mGIRK1, and mGIRK2 injected with or without PTX. Error bars represent mean ± SEM. Significance assessed by unpaired Student's t test, $^*p \leq 0.05$. See also Figure S5.

CellPress
OPEN ACCESS

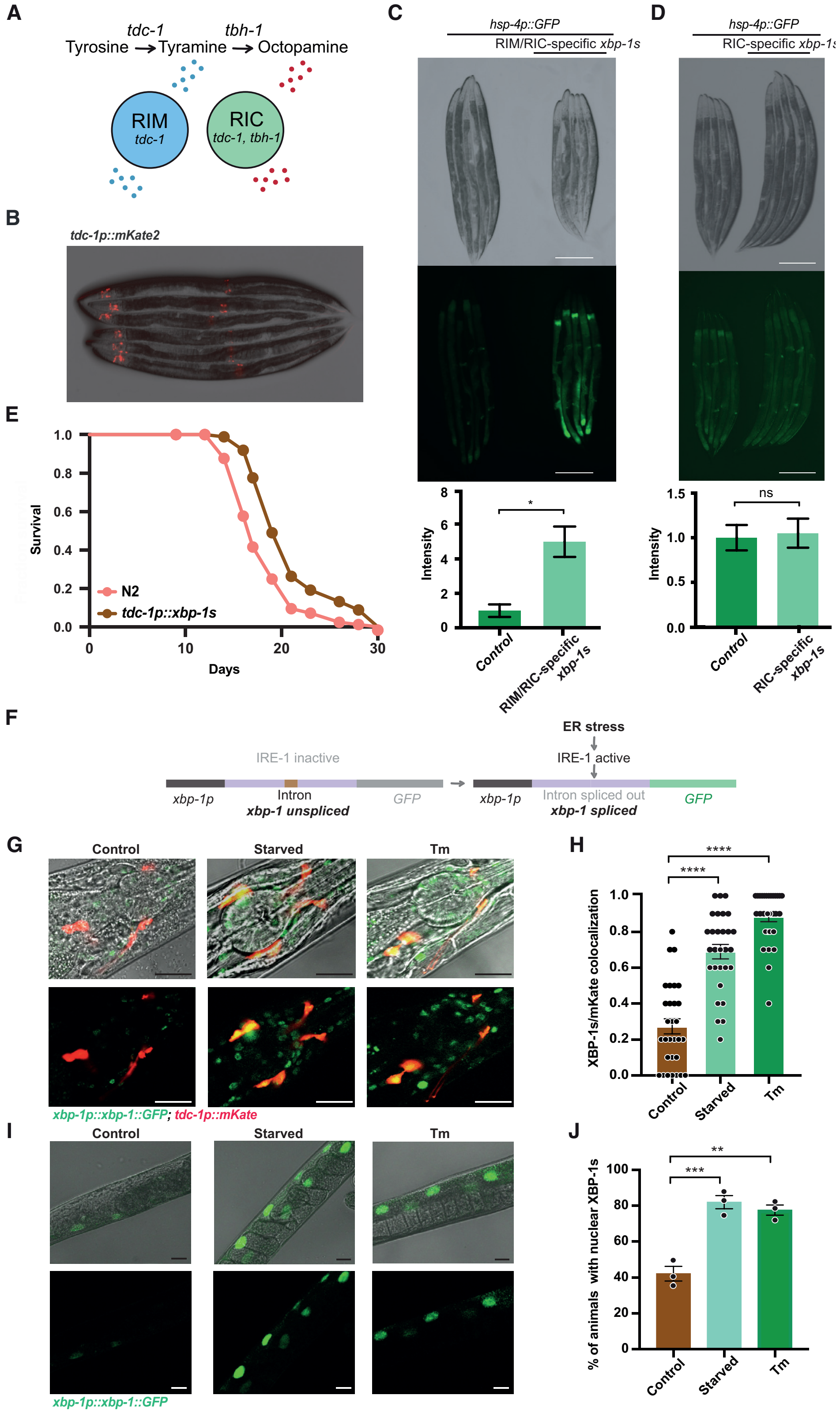

(legend on next page)

behavior. We hypothesized that *C. elegans* in which *xbp-1s* is constantly expressed within the nervous system might lack the ability to correctly perceive their nutritional status, with the presence of *xbp-1s* indicating a state of nutrient deprivation. As we know that, like nutrient deprivation, neuronal *xbp-1s* expression leads to enhanced lifespan, stress resistance, and metabolism (Daniele et al., 2020; Imanikia et al., 2019a, 2019b; Taylor and Dillin, 2013), we wondered whether it also leads to changes in behavior similar to those observed upon starvation, regardless of the actual nutritional environment. One important type of behavior that is responsive to changes in nutritional status is the response of animals to food, including foraging behavior as well the propensity to leave a depleting source of nutrition, which can be assessed using food leaving assays (Figure 5A) (Milward et al., 2011; Olofsson, 2014). Food leaving assays have shown that starved animals show a markedly reduced tendency to leave their food source (Olofsson, 2014). To determine the effects of neuronal *xbp-1s* on starvation-related behavior, we therefore began by measuring food leaving probability over a 9-h time course. This showed that animals expressing neuronal *xbp-1s* had a significantly decreased likelihood of leaving a bacterial lawn (Figure 5B). This was not caused by a defect in the speed of movement of these animals, which was not significantly different from wild type (Figure S7A). In addition, it was not caused by a decreased rate of pharyngeal pumping, which is not affected by pan-neuronal *xbp-1s* expression (Taylor and Dillin, 2013). Importantly, this food leaving phenotype could be suppressed by mutation of *tdc-1*, inhibiting tyramine synthesis, but not by abolishing synthesis of octopamine through mutation of *tbh-1*, suggesting a role for tyramine in mediating altered responses to food downstream of neuronal *xbp-1s* expression (Figures 5C and 5D).

We then asked whether mutations in tyramine receptors could also suppress the reduced food leaving phenotype of *rab-3p::xbp-1s* animals. While mutations in *tyra-3*, *tyra-2*, and *lgc-55* had no specific effects on food leaving probability in *rab-3p::xbp-1s* animals, *ser-2* mutation partially suppressed reduced food leaving (Figures 5E and S7**B**). Interestingly, we found that *ser-2* transcript levels were increased in neurons of *rab-3p::xbp-1s* animals, suggesting that this receptor is also regulated downstream of neuronal *xbp-1s* expression to modulate behavior (Figures 5F and 5G; Data S1). Although tyramine synthesis was required for suppressed food leaving in animals expressing pan-neuronal *xbp-1s*, expression of *xbp-1s* only in RIM and RIC neurons was not sufficient to alter food leaving probability, suggesting that *xbp-1s* expression in other neurons is also required—perhaps to induce the expression of tyraminergic receptors such as *ser-2* (Figure S7C).

To examine other aspects of feeding-related behavior, we also measured off-food exploration, which quantifies the degree and range of exploratory foraging movement of individual animals upon leaving a non-depleting (rather than a diminishing) food source (Figure 5H) (Pradhan et al., 2019). This again revealed altered feeding behavior, this time in conditions of nutritional abundance, with animals expressing neuronal *xbp-1s* exhibiting reduced exploration, which could be suppressed by mutation of *tdc-1* but not *tbh-1* (Figure 5I). Mutation of *ser-2* was unable to suppress this reduced food leaving phenotype, however, suggesting that another receptor may mediate the effect of neuronal *xbp-1s* on exploratory behavior in this context. In addition, we used an on-food exploration assay to measure exploratory behavior on a plate entirely coated with food, a measure of foraging that does not involve leaving a source of nutrition (Figure 5J) (Pradhan et al., 2019). Again, neuronal *xbp-1s*-expressing animals exhibited reduced foraging range, which was suppressed by mutation of *tdc-1* but not *tbh-1* or *ser-2* (Figure 5K). Animals expressing neuronal *xbp-1s* therefore exhibit reduced propensity for food leaving and exploratory behavior regardless of nutritional environment, dependent upon synthesis of tyramine, and, in at least one context, partially dependent upon expression of the tyraminergic receptor *ser-2*.

Neuronal *xbp-1s* Expression Reduces Brood Size

Reproduction is also altered in *C. elegans* as a response to changes in nutritional environment (Dong et al., 2000). Nutrient deprivation has been previously associated with reduced brood size, lower levels of embryogenesis, and increased rates of internal hatching (Chen and Caswell-Chen, 2004; Schafer, 2005; Seidel and Kimble, 2011). We therefore asked whether reproductive output was altered in neuronal *xbp-1s*-expressing animals. We first measured progeny production over time and found that brood size was reduced by neuronal *xbp-1s* and

Figure 4. Expression of *xbp-1s* in RIM and RIC Interneurons Induces Intestinal UPR^{ER} Activation, and *xbp-1* Is Spliced in These Neurons upon Stress

(A) Schematic representation of RIM and RIC interneurons highlighting cell-specific expression of *tdc-1* and *tbh-1*.

(B) Fluorescence microscopy of *tdc-1p::mKate* animals.

(C and D) Expression of (C) RIM/RIC-specific *tdc-1p::xbp-1s* or (D) RIC-specific *tbh-1p::xbp-1s* in *hsp-4p::GFP* animals alongside control *hsp-4p::GFP* worms. GFP levels were quantified using ImageJ and normalized to controls, and significance assessed by unpaired Student's t test, $^*p \leq 0.05$, ns = not significant. N $\geq$ 3 biological replicates containing $\geq$5 animals, error bars indicate SEM. Scalebars, 300 μm.

(E) Lifespan analysis of N2 and *tdc-1p::xbp-1s* animals (median lifespans 17 versus 19 days, $p < 0.0001$). Graphs were plotted as Kaplan-Meier survival curves, and p values calculated by Mantel-Cox log-rank test. N = 80–120 animals per condition in 3 biological replicates.

(F) Schematic representation of the *xbp-1* splicing reporter.

(G) Confocal microscopy of *xbp-1p::xbp-1::GFP; tdc-1p::mKate* animals untreated, subjected to overnight starvation, or treated with 25 mM tunicamycin for 4 h. Images show the region surrounding the pharynx. Scalebars, 20 μm.

(H) Quantification of confocal microscopy analysis in (G). The fraction of animals with GFP/mKate colocalization was quantified and significance relative to controls calculated by one-way ANOVA with Tukey's multiple comparisons test, $^{****}p \leq 0.0001$. N = 25–30 animals in 3 biological replicates.

(I) Confocal microscopy analysis of *xbp-1p::xbp-1::GFP* animals either untreated, subjected to overnight starvation, or treated with 25 mM tunicamycin for 4 h. Images show the mid-intestine. Scalebars, 20 μm.

(J) Quantification of confocal microscopy analysis in (I). The percentage of animals in which GFP was observed in intestinal nuclei was quantified and significance calculated by one-way ANOVA with Tukey's multiple comparisons test, $^{**}p \leq 0.01$, $^{***}p \leq 0.001$. N = 25–30 animals in 3 biological replicates. See also Figure S6; Table S1.

CellPress
OPEN ACCESS

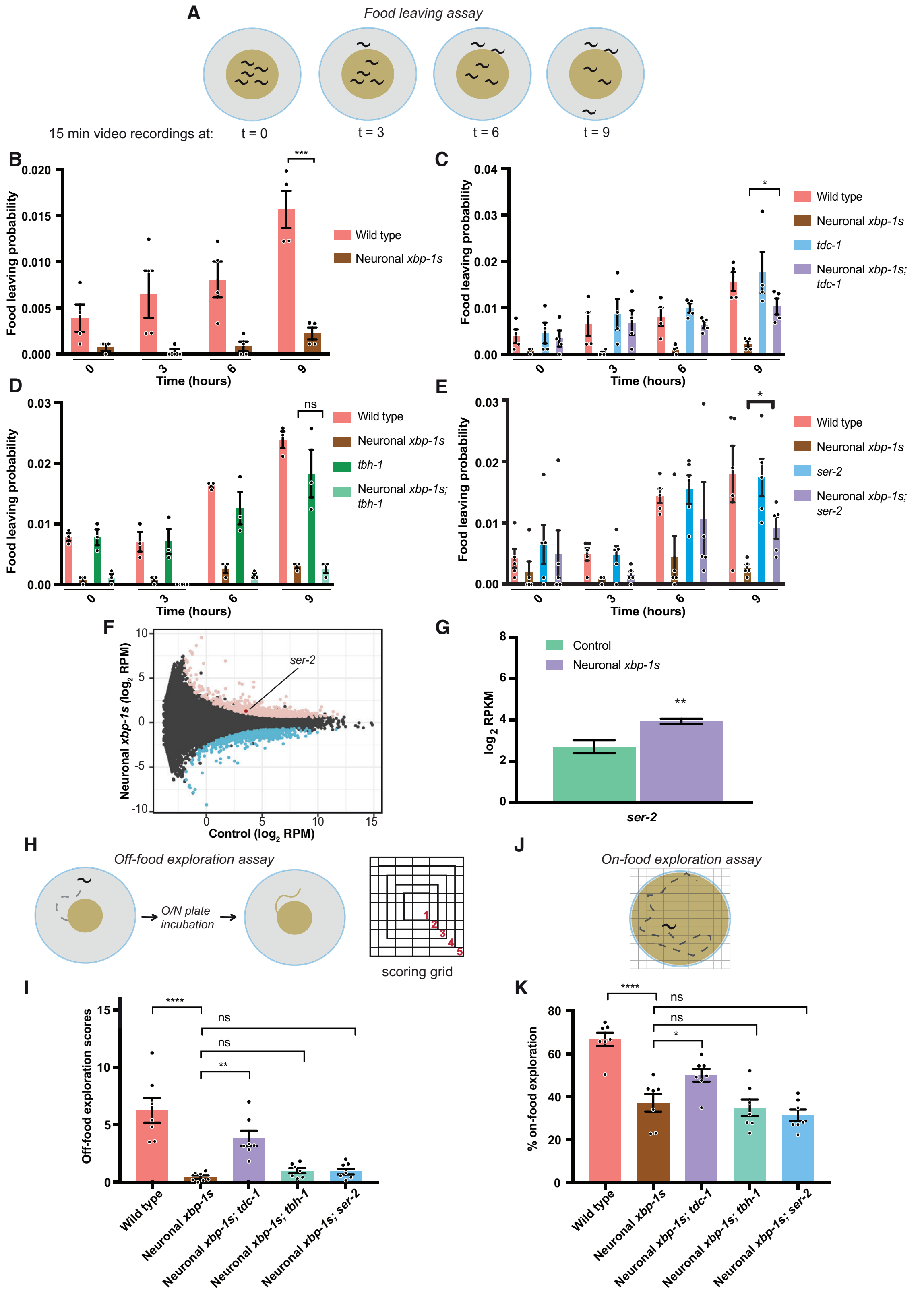

(legend on next page)

that this was suppressed by mutation of *tdc-1* but not *tbh-1* (Figures 6A and 6B). We then asked whether this might be the result of an increase in germline apoptosis in *rab-3p::xbp-1s* animals, as the UPR^{ER} has been previously implicated in regulation of germline cell death. However, quantification of apoptotic corpses in the germline showed no difference in these animals compared with wild type (Figures S7D–S7F) (Levi-Ferber et al., 2014). Next, we examined the number of eggs *in utero* in animals expressing neuronal *xbp-1s*. The average number of eggs in the uterus of each animal at 30 h post-L4 was reduced in *rab-3p::xbp-1s* worms, consistent with reduced progeny production (Figure 6C). Surprisingly, this reduced egg number was not suppressed in *tdc-1* mutant animals, despite their normal brood size. We then examined the stage of the eggs held within the uterus and found that while *rab-3p::xbp-1s* worms had reduced numbers of eggs at the 1–8 cell, 9–20 cell, and 21+ cell stages, *rab-3p::xbp-1s; tdc-1* animals had wild-type levels of 1–8 cell eggs but reduced numbers of 9–20 cell and especially 21+ cell embryos (Figures 6D and 6E). We hypothesize that, while mutation of *tdc-1* rescues egg production in *rab-3p::xbp-1s* animals, the effect on numbers of late-stage embryos and total embryos *in utero* might be masked by a previously described acceleration in the rate of egg laying in *tdc-1* mutants, resulting in their earlier expulsion from the uterus (Alkema et al., 2005).

We were interested to observe that some animals expressing neuronal *xbp-1s* had late, comma-stage embryos *in utero*, which were almost never seen in wild type and *rab-3p::xbp-1s; tdc-1* animals (Figures 6D and 6E). Suspecting that this retention of late-stage embryos might be linked to elevated levels of internal hatching in these animals, we measured the prevalence of internal hatching in *rab-3p::xbp-1s* populations. We found a striking elevation in this phenotype during late reproductive life in neuronal *xbp-1s* expressors relative to wild type (Figures 6F and S7**G**). This was again suppressed by mutation of *tdc-1*, suggesting that internal hatching may be a regulated phenotype in these worms.

Our results therefore suggest that reproductive strategy is altered in neuronal *xbp-1s*-expressing animals. While it is important to note that pan-neuronal *xbp-1s* expression may non-specifically affect the function of egg-laying neurons, the suppression of reduced brood size by mutation of *tdc-1* suggests an alternative interpretation: that altered tyramine-dependent signaling as a result of *xbp-1s* expression leads directly to reduced progeny production and increased internal hatching of embryos. Given that enhanced levels of *xbp-1s* expression in neurons might mimic a state of starvation in *C. elegans*, this may indicate that neuronal XBP-1s coordinates a global response to this stress that also includes changes to foraging behavior and induction of intestinal UPR^{ER} activation, with tyramine-dependent signaling playing a key role in each process. We therefore asked whether this XBP-1s-mediated global response might contribute to the extended lifespan seen upon dietary deprivation. We found that N2 animals live significantly longer when deprived of food in adulthood (23 days median lifespan) whereas longevity of animals expressing neuronal *xbp-1s* did not differ (25 days median lifespan), significantly narrowing the longevity gap between these strains (Figure 7A). *ire-1* and *xbp-1* mutants proved short lived relative to N2 under these conditions. This suggests that the extended lifespan of neuronal *xbp-1s*-expressing animals may operate through the same mechanisms that extend lifespan upon dietary deprivation and that UPR^{ER} components play a significant role in lifespan extension under these conditions. We therefore conclude that neuronal UPR^{ER} activation mediates a global response to stress that alters reproduction, behavior, metabolism, and longevity, dependent upon tyramine synthesis, to increase fitness in stressful environments (Figure 7B).

DISCUSSION

Our transcriptomic analysis reveals that, similar to the FOXO transcription factor DAF-16 (Kaletsky et al., 2016), XBP-1s has a distinct neuronal transcriptome that includes many genes specifically involved in nervous system function in *C. elegans*. These neuronal gene expression changes suggest that *xbp-1s* can regulate numerous aspects of neuronal cell biology and may broadly remodel neuronal signaling in this organism. A previous study identified effects of murine XBP1s on cognition and memory formation, dependent upon brain-derived neurotrophic factor (BDNF), suggesting that this neuronal signaling role may be conserved through evolution (Martínez et al., 2016).

Neuronal *xbp-1s* triggers the transmission of UPR^{ER} activation to the intestine of *C. elegans*, utilizing a hitherto uncharacterized signaling pathway (Taylor and Dillin, 2013). Previous findings

Figure 5. Neuronal *xbp-1s* Expression Alters Food Leaving and Foraging Behavior, Dependent upon Tyramine Synthesis

(A) Schematic representation of the food leaving assay.

(B–E) Food leaving probability, with individual probabilities plotted for each biological replicate (N $\geq$ 3), as well as the mean. Error bars represent SEM. Significance was assessed using two-way ANOVA with Tukey's multiple comparison test, *p $\leq$ 0.05, ***p $\leq$ 0.001, ns = not significant.

(F) MA plot showing transcripts in neuronal cells with and without neuronal *xbp-1s* expression. Upregulated transcripts are highlighted in pink, downregulated in blue, and the upregulated transcript *ser-2* is annotated. RPM, reads per million mapped reads

(G) Expression levels of *ser-2* in *unc-119p::GFP* versus *unc-119p::GFP; rab-3p::xbp-1s* animals in neuron-specific RNA-seq analysis. RPKM = reads per kilobase per million mapped reads. Error bars represent SEM from 3 biological replicates. Significance assessed using DeSeq2 with FDR correction, **p $\leq$ 0.01.

(H) Schematic representation of the off-food exploration assay.

(I) Off-food exploration scores for N2 wild type, *rab-3p::xbp-1s*, *tdc-1(n3419)*; *rab-3p::xbp-1s*, *tbh-1(rms2); rab-3p::xbp-1s*, and *ser-2(rms3); rab-3p::xbp-1s* animals. Mean score in $\geq$4 worms per replicate was calculated and individual biological replicates (N $\geq$ 5) plotted as well as the mean; error bars indicate SEM. Significance relative to *rab-3p::xbp-1s* animals was calculated by one-way ANOVA with Dunnett's multiple comparison test, **p $\leq$ 0.01, ****p $\leq$ 0.0001, ns = not significant.

(J) Schematic representation of the on-food exploration assay.

(K) On-food exploration scores for N2 wild type, *rab-3p::xbp-1s*, *tdc-1(n3419)*; *rab-3p::xbp-1s*, *tbh-1(rms2); rab-3p::xbp-1s*, and *ser-2(rms3); rab-3p::xbp-1s* animals. Mean score in $\geq$4 worms per replicate is shown as well as the overall mean (N $\geq$ 5); error bars indicate SEM. Significance relative to *rab-3p::xbp-1s* animals was calculated by one-way ANOVA with Dunnett's multiple comparison test, *p $\leq$ 0.05, ****p $\leq$ 0.0001, ns = not significant. See also Figure S7.

CellPress
OPEN ACCESS

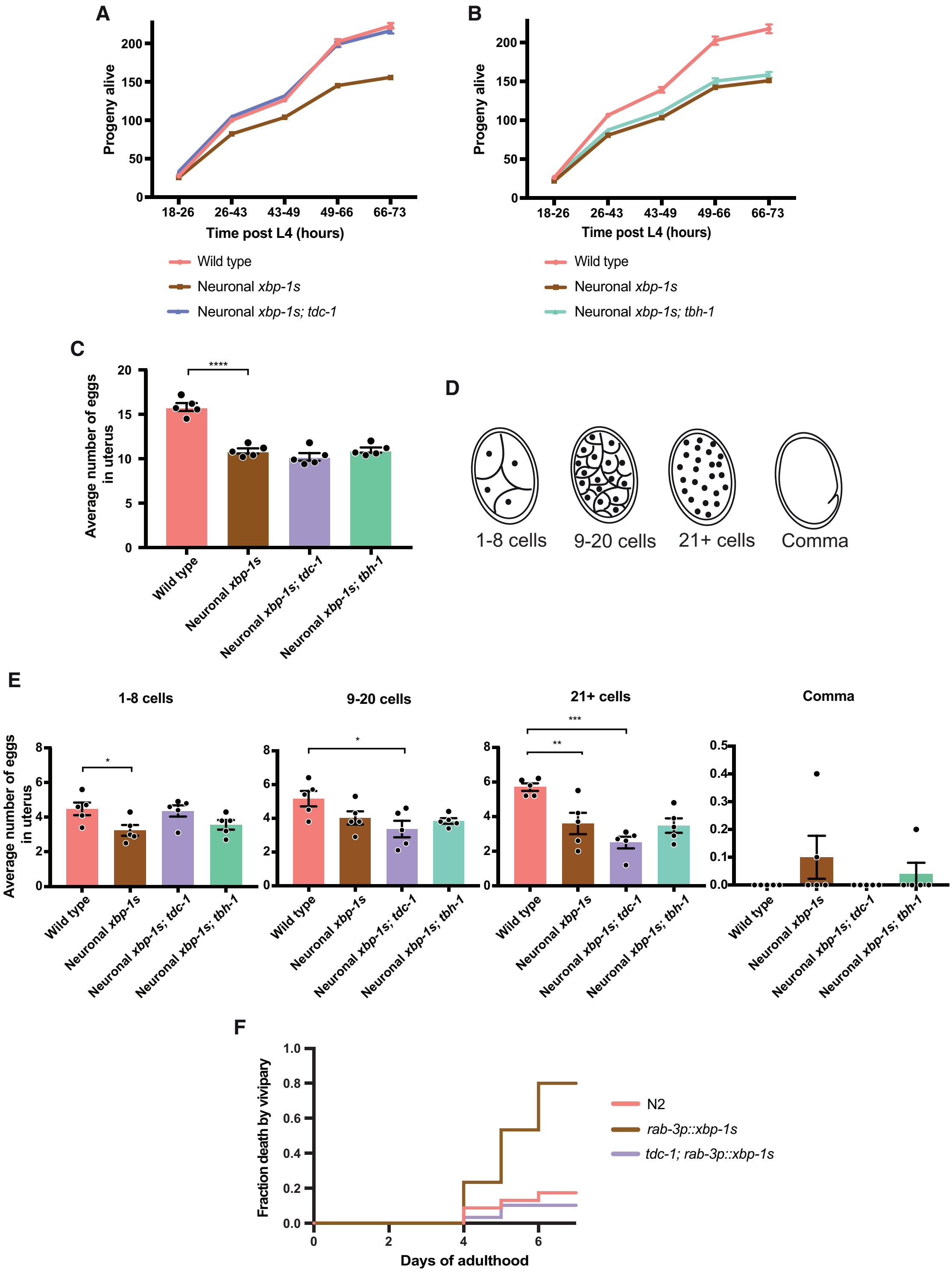

Figure 6. Neuronal *xbp-1s* Expression Alters Brood Size, Dependent upon Tyramine Synthesis

(A and B) Progeny hatched from eggs laid by N2, *rab-3p::xbp-1s*, and (A) *tdc-1(n3419); rab-3p::xbp-1s* or (B) *tbh-1(rms2); rab-3p::xbp-1s* worms during the first 3 days of adulthood. Mean number of offspring per worm from ~50 worms is plotted per genotype; error bars indicate SEM.

(C) Average number of eggs *in utero* of N2, *rab-3p::xbp-1s*, *tdc-1(n3419)*; *rab-3p::xbp-1s* and *tbh-1(rms2); rab-3p::xbp-1s* worms at 30 h post-L4 larval stage in 10 animals per replicate. The mean for each replicate is plotted as well as the overall mean (N = 5). Error bars represent SEM. Significance relative to *rab-3p::xbp-1s* was assessed by one-way ANOVA with Dunnett's multiple comparison test, ****$p \leq 0.0001$.

(D) Schematic representation of *C. elegans* embryos at the 1–8, 9–20, and 21+ cell and comma stages.

(legend continued on next page)

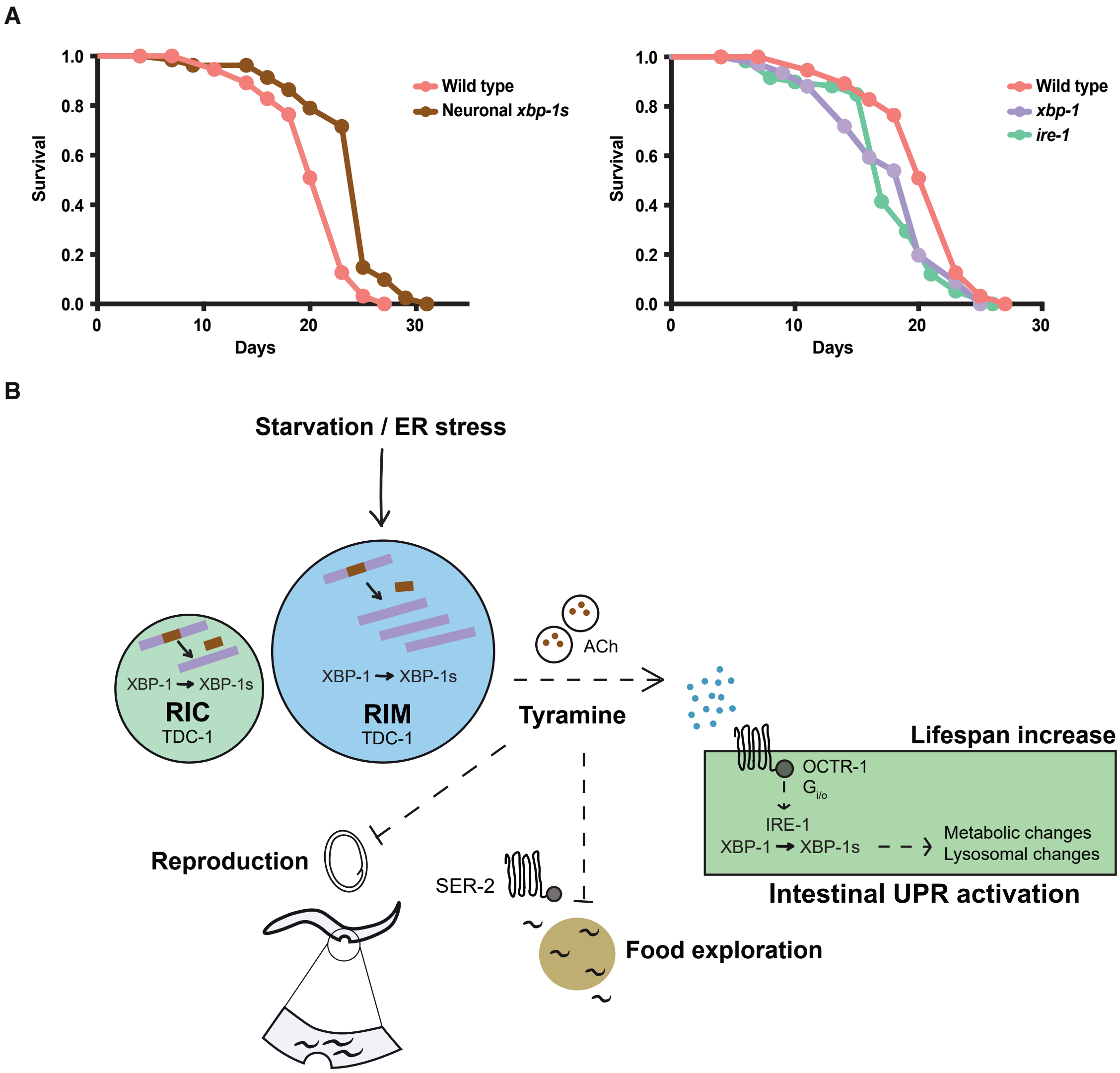

Figure 7. Neuronal XBP-1s Mediates a Global Survival Response to Environmental Stress

(A) Lifespan analysis upon dietary deprivation of N2 versus *rab-3p::xbp-1s* (median lifespans 23 versus 25 days), and N2 versus *ire-1(v33)* and *xbp-1(zc12)* animals (median lifespans 23 versus 17 days and 20 days). Graphs were plotted as Kaplan-Meier survival curves, and p values calculated by the Gehan-Breslow-Wilcoxon test. N = 80–120 animals per condition in 3 biological replicates. See also Table S1.

(B) Model showing downstream effects of *xbp-1* splicing in *C. elegans* neurons. ACh, acetylcholine.

have demonstrated that this signaling depends upon the secretory regulator UNC-13, but not UNC-31, which is required for the release of neuropeptides from dense core vesicles. This suggests that the mechanisms involved are likely to be different from those acting in a recently described pathway mediating transmission of UPR^{ER} activation from glia to the intestine, which relies upon neuropeptide release (Frakes et al., 2020). We show that this signaling mechanism may be complex, requiring both acetylcholine and the biogenic amine tyramine. Indeed, previous work demonstrated that treatment with single neurotransmitters, including tyramine and octopamine, is insufficient to induce intestinal UPR^{ER} activation in *hsp-4::GFP* animals, suggesting that multiple molecules may be required (Taylor and Dillin, 2013). This resembles the cell non-autonomous transmission

(E) Average number of embryos at the 1–8, 9–20, and 21+ cell and comma stages in the uterus of N2, *rab-3p::xbp-1s*, *tdc-1(n3419)*; *rab-3p::xbp-1s* and *tbh-1(rms2); rab-3p::xbp-1s* worms at 30 h post-L4 in 10 animals per replicate. The mean for each replicate is plotted as well as the overall mean (N = 5). Error bars represent SEM. Significance relative to *rab-3p::xbp-1s* was assessed by one-way ANOVA with Dunnett's multiple comparison test, *p $\leq$ 0.05, **p $\leq$ 0.01, ****p $\leq$ 0.0001.

(F) Numbers of N2, *rab-3p::xbp-1s*, *tdc-1(n3419)*; *rab-3p::xbp-1s* and *tbh-1(rms2); rab-3p::xbp-1s* animals in which internal hatching was observed was counted for the first 6 days of adulthood and the fraction of the population undergoing internal hatching plotted (N = 3). Significance was calculated by Mantel-Cox log-rank test, p < 0.0001 between *rab-3p::xbp-1s* and N2, and between *rab-3p::xbp-1s* and *rab-3p::xbp-1s*; *tdc-1(n3419)*. See also Figure S7.

of the UPR^{mt} in *C. elegans*, in which both serotonin and Wnt signaling are implicated (Berendzen et al., 2016; Zhang et al., 2018). Nevertheless, expression of *xbp-1s* in only the *tdc-1*-expressing RIM and RIC interneurons is sufficient to induce intestinal UPR^{ER} activation and extend longevity, showing that this very restricted set of neurons plays a key role in this signaling event and that tyramine synthesis is likely to be an important element of this process.

Biogenic amines have important functions in animal behavior, and tyramine itself acts as a signaling molecule in both invertebrates and vertebrates. In *C. elegans*, tyramine acts both synaptically and extra-synaptically, and its roles include the modulation of egg laying, reversal behavior, and head oscillation (Alkema et al., 2005; Bentley et al., 2016; Pirri et al., 2009). In addition, a recent study has also implicated tyramine in the regulation of stress response activation in the intestine, although these findings differed from ours in suggesting that tyramine can inhibit the induction of cytoprotective genes associated with resistance to oxidative and other stresses, and in suggesting that stress can downregulate RIM activity (De Rosa et al., 2019). This effect was mediated through alterations to the insulin-like signaling pathway, in favor of an acute "flight" response to stress. In contrast, we found that knockdown of *daf-16* did not affect UPR^{ER} induction in *rab-3p*::*xbp-1s* or *rab-3p::xbp-1s; tdc-1* animals (Figure S4B). Our results also contrast with others that suggest a suppression of RIM activity upon stress (Ghosh et al., 2016; Jin et al., 2016). It will be interesting in future to determine the specific role of the RIM interneurons in inter-tissue UPR^{ER} signaling, through the use of *xbp-1s* driven by RIM-specific promoters that do not cause expression in RIC neurons, and by monitoring the activation of the RIM neurons under different conditions of UPR^{ER} activation.

It is not clear whether, in the context of neuronally regulated intestinal UPR^{ER} activation, tyramine functions synaptically, through a network of cells, or whether it could act in a hormone-like manner. The possibility that the biogenic amine receptor OCTR-1 is involved in this pathway, and that it functions in the intestine, might indicate the latter mode of action. OCTR-1 has been previously described as an octopaminergic receptor that functions in the ASH and ASI neurons to inhibit ER stress responses in other tissues (Sun et al., 2011, 2012; Wragg et al., 2007). Our results suggest that OCTR-1 responds to tyramine as well as octopamine, and that it can function within the intestine to regulate UPR^{ER} activation, suggesting that this receptor could provide a link between tyramine release from neurons and *xbp-1s* activation in the intestine. This relationship is not straightforward, however, as *octr-1* levels are reduced upon *xbp-1s* expression, and it is knockdown of *octr-1* that promotes UPR^{ER} activation; how would binding of tyramine to OCTR-1 then lead to reduced receptor levels and consequent UPR^{ER} activation? One possibility might be that the binding of tyramine to OCTR-1 acts to trigger receptor downregulation, via the activation of the OCTR-1-related $G_{i/o}$ pathway, which then leads to UPR^{ER} activation. However, this suggestion is speculative; clearly an unconventional mechanism is at work here, and further investigation of this regulatory process is needed to better define its mechanistic basis.

It will also be interesting to explore the UPR^{ER}-related roles of other neurotransmitters identified through our candidate mutant screening (Figure 2B). Importantly, disruption of octopamine synthesis reduces cell non-autonomous UPR^{ER} activation, albeit to a lesser extent than loss of tyramine synthesis, and the possibility that octopaminergic signaling plays a role in inter-tissue UPR^{ER} signaling remains open. In addition, study of acetylcholine signaling in this context may help to clarify its proteostasis-related functions, which have been previously identified in a study exploring the effects of neuronal signaling on proteotoxicity in *C. elegans* muscles (Garcia et al., 2007). We also found that mutations in the *daf-7*/*daf-1* TGF-β pathway enhance intestinal UPR^{ER} activation upon neuronal *xbp-1s* expression. DAF-7 is produced in the ASI sensory neurons, which respond to nutrient-related cues and interact with the RIM and RIC interneurons; in addition, it regulates behaviors that include egg laying and food leaving, and is downregulated upon starvation (Gallagher et al., 2013; Ren et al., 1996). This suggests that *daf-7* might connect the activation of the UPR^{ER} in RIM and RIC neurons with environmental perception.

Nutrient deprivation is a highly physiologically relevant scenario in which the UPR^{ER} is activated in the neurons of *C. elegans*. The mechanism mediating this activation is intriguing. It is possible that UPR^{ER} activation occurs in multiple neurons as a direct result of a lack of nutrients such as glucose, which is required at high levels for neuronal metabolism and a shortage of which can result in failure of glycosylation and consequent induction of an ER stress response (Munro and Pelham, 1986). Alternatively, perception of nutrient conditions by sensory neurons might trigger UPR^{ER} activation in downstream neurons. This could result from the detection of external cues related to nutrient presence, or from internal, calorie-associated signals, analogous to the activation of AgRP neurons upon food removal in mice (Su et al., 2017). The resulting inter-tissue UPR^{ER} signaling then induces starvation-associated metabolic responses in the intestine, as we and others have observed, that promote survival in low nutrient conditions, including increased lysosomal catabolism and consumption of intestinal fat stores (Daniele et al., 2020; Imanikia et al., 2019a, 2019b). A role for the UPR^{ER} in the response to starvation in *C. elegans*, involving induction of lipases by IRE-1 and HSP-4, has previously been identified, demonstrating the importance of this pathway in survival upon nutrient deprivation (Jo et al., 2009). In addition, a requirement for IRE-1 in the resumption of development following larval starvation has also been demonstrated (Roux et al., 2016). Our results suggest that neuronal UPR^{ER} activation could act as an early detector of altered nutrient conditions, changing behavior and sending signals to the intestine to induce UPR^{ER} activation before homeostasis is disrupted in that tissue.

Increased octopamine biosynthesis has been observed in *C. elegans* neurons during starvation, through upregulation of *tbh-1* expression in RIC neurons (Tao et al., 2016). Octopamine then upregulates an intestinal lipase through activation of the SER-3 receptor. In vertebrates, elevated levels of the biogenic amine norepinephrine, for which octopamine and possibly tyramine are functional analogs in *C. elegans*, is a common response to starvation, suggesting that this signaling pathway may be a functionally conserved response to nutrient deprivation (De Pedro et al., 2003; Zauner et al., 2000). Indeed, a recent study found that food perception induces UPR^{ER} activation, mTOR signaling, and changes to lipid metabolism in the liver of mice through a

hypothalamic circuit involving the POMC neurons, with norepinephrine playing a role upstream of hepatic *xbp1* splicing (Brandt et al., 2018). Coupled with a previous observation that *xbp1s* expression in POMC neurons results in UPR^{ER} activation in the liver and improved systemic glucose homeostasis (Williams et al., 2014), this suggests that UPR^{ER}-mediated responses to nutrient conditions may coordinate systemic metabolic changes in both invertebrates and vertebrates.

Dietary restriction is associated with increased longevity, altered behavior, and decreased reproduction in *C. elegans*. Similarly, neuronal UPR^{ER} activation extends longevity and also changes feeding behavior and reduces reproductive output. It has been shown that animals fail to coordinate appropriate food leaving when they cannot correctly interpret their nutritional state (Olofsson, 2014), and it is possible that neuronal *xbp-1* splicing may act as an indicator of nutritional conditions. Monoamines including both tyramine and octopamine modulate movement, in addition to their ability to communicate with the intestine, so are well situated to coordinately regulate motile behavior and intestinal physiology (Brown et al., 2013; Yemini et al., 2013). Interestingly, a recent paper implicated the production of tyramine by bacteria in the intestine, and the OCTR-1 receptor, in mediating changes to aversive behavior, suggesting that dietary information conveyed through tyramine-mediated communication between the intestine and neurons may operate in both directions (O'Donnell et al., 2020). Our findings that OCTR-1 can respond to tyramine and function in the intestine suggests that this communication could operate through a mechanism more direct and local to the intestine than that proposed by the authors. It will be interesting in future to determine whether OCTR-1 is also involved in mediating the behavioral changes we observe upon neuronal *xbp-1s* expression. Monoamines also mediate changes to egg-laying behavior (Alkema et al., 2005; Horvitz et al., 1982). Egg laying is stimulated by food; inhibition of egg laying and reduced brood size upon starvation could promote the delay of progeny release until conditions improve and reduce competition for resources. In addition, we suggest that increased rates of internal hatching might confer protection and a source of food to young hatching in stressful conditions, providing a nutritional "springboard" to kickstart development in low nutrient conditions.

Overall, our results suggest that neuronal splicing and activation of XBP-1 may act as an indicator of environmental stress, including low nutrient conditions, and that the neuronal targets of this transcription factor allow it to coordinate behavioral and reproductive responses to stress with signaling to the intestine. Distal activation of XBP-1 in this tissue then regulates expression of intestinal target genes that promote proteostasis, longevity, and metabolic remodeling. Further understanding of the elements of this global response to stress may allow its manipulation to promote cellular health and longevity.

STAR★METHODS

Detailed methods are provided in the online version of this paper and include the following:

- KEY RESOURCE TABLE
- RESOURCE AVAILABILITY
  - Lead Contact
  - Materials Availability
  - Data and Code Availability
- EXPERIMENTAL MODEL AND SUBJECT DETAILS
  - C. elegans Maintenance
  - Xenopus Laevis Oocyte Maintenance
- METHOD DETAILS
  - C. elegans Cell Dissociation
  - C. elegans Cell Isolation by FACS
  - RNA Extraction and Amplification
  - Library Preparation and RNA Sequencing
  - Assessment of Binding Site Frequency
  - Quantitative RT-PCR
  - Generation of Transgenic C. elegans Strains
  - Protein-Based CRISPR for Mutagenesis
  - Fluorescence Microscopy
  - Confocal Microscopy
  - Lifespan Analysis
  - Dietary Deprivation Assays
  - Gene Expression in *Xenopus* Oocytes
  - RNA Synthesis and Microinjection
  - Two-Electrode Voltage Clamp Recording
  - TEVC Data Analysis and Plotting
  - Worm Speed Measurement
  - Food Leaving Assay
  - Off-Food Exploration Assay
  - On-Food Exploration Assay
  - Brood Size Assay
  - Eggs *In Utero* and Egg Staging Assays
  - Germline Apoptosis Assays
- QUANTIFICATION AND STATISTICAL ANALYSIS

SUPPLEMENTAL INFORMATION

Supplemental Information can be found online at https://doi.org/10.1016/j.devcel.2020.10.024.

ACKNOWLEDGMENTS

We thank all members of the Taylor lab for helpful discussion and comments on the manuscript. We would also like to thank Dr. Denise Walker, Dr. Kristin Webster, Dr. Yee Lian Chew, Dr. Yiquan Tang, Dr. Mario De Bono, Dr. Birgitta Olofsson, and Dr. Sean Flynn for *C. elegans* strains, reagents, and expertise. We are grateful to the MRC LMB flow cytometry, microscopy, and media facilities for expertise and assistance, the MRC LMB Visual Aids department for help with graphics, and the CRUK Genomics Core for sequencing services. Some strains were provided by the CGC, which is funded by NIH Office of Research Infrastructure Programs (P40 OD010440). This work was supported by the Medical Research Council.

AUTHOR CONTRIBUTIONS

N.P.O. and R.C.T. conceived, performed, and analyzed experiments and wrote the manuscript. N.P.O. also developed methodology and curated data. S.I. performed and analyzed experiments. C.K. analyzed data and provided expertise. I.H. and J.M.L. conceived, performed, and analyzed experiments and provided expertise. M.S. developed methodology and provided reagents. W.R.S. provided expertise and resources. M.O.C. provided expertise and resources and reviewed the manuscript.

DECLARATION OF INTERESTS

The authors declare no competing interests.

CellPress
OPEN ACCESS

Received: June 28, 2020
Revised: July 17, 2020
Accepted: October 30, 2020
Published: November 23, 2020

REFERENCES

Acosta-Alvear, D., Zhou, Y., Blais, A., Tsikitis, M., Lents, N.H., Arias, C., Lennon, C.J., Kluger, Y., and Dynlacht, B.D. (2007). XBP1 controls diverse cell type- and condition-specific transcriptional regulatory networks. Mol. Cell *27*, 53–66.

Alkema, M.J., Hunter-Ensor, M., Ringstad, N., and Horvitz, H.R. (2005). Tyramine Functions independently of octopamine in the *Caenorhabditis elegans* nervous system. Neuron *46*, 247–260.

Bentley, B., Branicky, R., Barnes, C.L., Chew, Y.L., Yemini, E., Bullmore, E.T., Vértes, P.E., and Schafer, W.R. (2016). The multilayer connectome of *Caenorhabditis elegans*. PLoS Comput. Biol. *12*, e1005283.

Berendzen, K.M., Durieux, J., Shao, L.W., Tian, Y., Kim, H.E., Wolff, S., Liu, Y., and Dillin, A. (2016). Neuroendocrine coordination of mitochondrial stress signaling and proteostasis. Cell *166*, 1553–1563.e10.

Brandt, C., Nolte, H., Henschke, S., Engström Ruud, L., Awazawa, M., Morgan, D.A., Gabel, P., Sprenger, H.G., Hess, M.E., Günther, S., et al. (2018). Food perception primes hepatic ER homeostasis via melanocortin-dependent control of mTOR activation. Cell *175*, 1321–1335.e20.

Brenner, S. (1974). The genetics of *Caenorhabditis elegans*. Genetics *77*, 71.

Brown, A.E., Yemini, E.I., Grundy, L.J., Jucikas, T., and Schafer, W.R. (2013). A dictionary of behavioral motifs reveals clusters of genes affecting *Caenorhabditis elegans* locomotion. Proc. Natl. Acad. Sci. USA *110*, 791–796.

Burkewitz, K., Morantte, I., Weir, H.J.M., Yeo, R., Zhang, Y., Huynh, F.K., Ilkayeva, O.R., Hirschey, M.D., Grant, A.R., and Mair, W.B. (2015). Neuronal CRTC-1 governs systemic mitochondrial metabolism and lifespan via a catecholamine signal. Cell *160*, 842–855.

Calfon, M., Zeng, H., Urano, F., Till, J.H., Hubbard, S.R., Harding, H.P., Clark, S.G., and Ron, D. (2002). IRE1 couples endoplasmic reticulum load to secretory capacity by processing the XBP-1 mRNA. Nature *415*, 92–96.

Chen, J., and Caswell-Chen, E.P. (2004). Facultative vivipary is a life-history trait in *Caenorhabditis elegans*. J. Nematol. *36*, 107–113.

Clauss, I.M., Chu, M., Zhao, J.L., and Glimcher, L.H. (1996). The basic domain/leucine zipper protein hXBP-1 preferentially binds to and transactivates CRE-like sequences containing an ACGT core. Nucleic Acids Res. *24*, 1855–1864.

Cox, J.S., and Walter, P. (1996). A novel mechanism for regulating activity of a transcription factor that controls the unfolded protein response. Cell *87*, 391–404.

Daniele, J.R., Higuchi-Sanabria, R., Durieux, J., Monshietehadi, S., Ramachandran, V., Tronnes, S.U., Kelet, N., Sanchez, M., Metcalf, M.G., Garcia, G., et al. (2020). UPR^{ER} promotes lipophagy independent of chaperones to extend life span. Sci. Adv. *6*, eaaz1441.

De Pedro, N., Delgado, M.J., Gancedo, B., and Alonso-Bedate, M. (2003). Changes in glucose, glycogen, thyroid activity and hypothalamic catecholamines in tench by starvation and refeeding. J. Comp. Physiol. B *173*, 475–481.

De Rosa, M.J., Veuthey, T., Florman, J., Grant, J., Blanco, M.G., Andersen, N., Donnelly, J., Rayes, D., and Alkema, M.J. (2019). The flight response impairs cytoprotective mechanisms by activating the insulin pathway. Nature *573*, 135–138.

Dong, M.Q., Chase, D., Patikoglou, G.A., and Koelle, M.R. (2000). Multiple RGS proteins alter neural G protein signaling to allow *C. elegans* to rapidly change behavior when fed. Genes Dev. *14*, 2003–2014.

Frakes, A.E., Metcalf, M.G., Tronnes, S.U., Bar-Ziv, R., Durieux, J., Gildea, H.K., Kandahari, N., Monshietehadi, S., and Dillin, A. (2020). Four glial cells regulate ER stress resistance and longevity via neuropeptide signaling in *C. elegans*. Science *367*, 436–440.

Gallagher, T., Kim, J., Oldenbroek, M., Kerr, R., and You, Y.J. (2013). ASI regulates satiety quiescence in *C. elegans*. J. Neurosci. *33*, 9716–9724.

Garcia, S.M., Casanueva, M.O., Silva, M.C., Amaral, M.D., and Morimoto, R.I. (2007). Neuronal signaling modulates protein homeostasis in *Caenorhabditis elegans* post-synaptic muscle cells. Genes Dev. *21*, 3006–3016.

Ghosh, D.D., Sanders, T., Hong, S., McCurdy, L.Y., Chase, D.L., Cohen, N., Koelle, M.R., and Nitabach, M.N. (2016). Neural architecture of hunger-dependent multisensory decision making in *C. elegans*. Neuron *92*, 1049–1062.

Grant, C.E., Bailey, T.L., and Noble, W.S. (2011). FIMO: scanning for occurrences of a given motif. Bioinformatics *27*, 1017–1018.

Gumienny, T.L., Lambie, E., Hartwieg, E., Horvitz, H.R., and Hengartner, M.O. (1999). Genetic control of programmed cell death in the *Caenorhabditis elegans* hermaphrodite germline. Development *126*, 1011–1022.

Hardege, I., Xu, S., Gordon, R.D., Thompson, A.J., Figg, N., Stowasser, M., Murrell-Lagnado, R., and O'Shaughnessy, K.M. (2015). Novel insertion mutation in KCNJ5 channel produces constitutive aldosterone release From H295R cells. Mol. Endocrinol. *29*, 1522–1530.

Henis-Korenblit, S., Zhang, P., Hansen, M., McCormick, M., Lee, S.J., Cary, M., and Kenyon, C. (2010). Insulin/IGF-1 signaling mutants reprogram ER stress response regulators to promote longevity. Proc. Natl. Acad. Sci. USA *107*, 9730–9735.

Horvitz, H.R., Chalfie, M., Trent, C., Sulston, J.E., and Evans, P.D. (1982). Serotonin and octopamine in the nematode *Caenorhabditis elegans*. Science *216*, 1012–1014.

Imanikia, S., Özbey, N.P., Krueger, C., Casanueva, M.O., and Taylor, R.C. (2019a). Neuronal XBP-1 activates intestinal lysosomes to improve proteostasis in *C. elegans*. Curr. Biol. *29*, 2322–2338.e7.

Imanikia, S., Sheng, M., Castro, C., Griffin, J.L., and Taylor, R.C. (2019b). XBP-1 remodels lipid metabolism to extend longevity. Cell Rep. *28*, 581–589.e4.

Jin, X., Pokala, N., and Bargmann, C.I. (2016). Distinct circuits for the formation and retrieval of an imprinted olfactory memory. Cell *164*, 632–643.

Jo, H., Shim, J., Lee, J.H., Lee, J., and Kim, J.B. (2009). IRE-1 and HSP-4 contribute to energy homeostasis via fasting-induced lipases in *C. elegans*. Cell Metab. *9*, 440–448.

Kaletsky, R., Lakhina, V., Arey, R., Williams, A., Landis, J., Ashraf, J., and Murphy, C.T. (2016). The *C. elegans* adult neuronal IIS/FOXO transcriptome reveals adult phenotype regulators. Nature *529*, 92–96.

Kaletsky, R., Yao, V., Williams, A., Runnels, A.M., Tadych, A., Zhou, S., Troyanskaya, O.G., and Murphy, C.T. (2018). Transcriptome analysis of adult *Caenorhabditis elegans* cells reveals tissue-specific gene and isoform expression. PLoS Genet. *14*, e1007559.

Kamath, R.S., Martinez-Campos, M., Zipperlen, P., Fraser, A.G., and Ahringer, J. (2001). Effectiveness of specific RNA-mediated interference through ingested double-stranded RNA in *Caenorhabditis elegans*. Genome Biol. *2*, research0002.

Levi-Ferber, M., Salzberg, Y., Safra, M., Haviv-Chesner, A., Bülow, H.E., and Henis-Korenblit, S. (2014). It's all in your mind: determining germ cell fate by neuronal IRE-1 in *C. elegans*. PLoS Genet. *10*, e1004747.

Martínez, G., Vidal, R.L., Mardones, P., Serrano, F.G., Ardiles, A.O., Wirth, C., Valdés, P., Thielen, P., Schneider, B.L., Kerr, B., et al. (2016). Regulation of memory formation by the transcription factor XBP1. Cell Rep. *14*, 1382–1394.

Milward, K., Busch, K.E., Murphy, R.J., de Bono, M., and Olofsson, B. (2011). Neuronal and molecular substrates for optimal foraging in *Caenorhabditis elegans*. Proc. Natl. Acad. Sci. USA *108*, 20672–20677.

Munro, S., and Pelham, H.R. (1986). An Hsp70-like protein in the ER: identity with the 78 KD glucose-regulated protein and immunoglobulin heavy chain binding protein. Cell *46*, 291–300.

O'Donnell, M.P., Fox, B.W., Chao, P.H., Schroeder, F.C., and Sengupta, P. (2020). A neurotransmitter produced by gut bacteria modulates host sensory behaviour. Nature *583*, 415–420.

Olofsson, B. (2014). The olfactory neuron AWC promotes avoidance of normally palatable food following chronic dietary restriction. J. Exp. Biol. *217*, 1790–1798.

Paix, A., Folkmann, A., Rasoloson, D., and Seydoux, G. (2015). High efficiency, homology-directed genome editing in *Caenorhabditis elegans* using CRISPR-Cas9 ribonucleoprotein complexes. Genetics *201*, 47–54.

Pirri, J.K., McPherson, A.D., Donnelly, J.L., Francis, M.M., and Alkema, M.J. (2009). A tyramine-gated chloride channel coordinates distinct motor programs of a *Caenorhabditis elegans* escape response. Neuron *62*, 526–538.

Pradhan, S., Quilez, S., Homer, K., and Hendricks, M. (2019). Environmental programming of adult foraging behavior in *C. elegans*. Curr. Biol. *29*, 2867–2879.e4.

Ren, P., Lim, C.S., Johnsen, R., Albert, P.S., Pilgrim, D., and Riddle, D.L. (1996). Control of *C. elegans* larval development by neuronal expression of a TGF-beta homolog. Science *274*, 1389–1391.

Ringstad, N., and Horvitz, H.R. (2008). Fmrfamide neuropeptides and acetylcholine synergistically inhibit egg-laying by *C. elegans*. Nat. Neurosci. *11*, 1168–1176.

Roux, A.E., Langhans, K., Huynh, W., and Kenyon, C. (2016). Reversible age-related phenotypes induced during larval quiescence in *C. elegans*. Cell Metab. *23*, 1113–1126.

Schafer, W.R. (2005). Egg-laying. WormBook, 1–7.

Seidel, H.S., and Kimble, J. (2011). The oogenic germline starvation response in *C. elegans*. PLoS ONE *6*, e28074.

Shen, X., Ellis, R.E., Sakaki, K., and Kaufman, R.J. (2005). Genetic interactions due to constitutive and inducible gene regulation mediated by the unfolded protein response in *C. elegans*. PLoS Genet. *1*, e37.

Shim, J., Umemura, T., Nothstein, E., and Rongo, C. (2004). The unfolded protein response regulates glutamate receptor export from the endoplasmic reticulum. Mol. Biol. Cell *15*, 4818–4828.

Silva-García, C.G., Lanjuin, A., Heintz, C., Dutta, S., Clark, N.M., and Mair, W.B. (2019). Single-copy knock-in loci for defined gene expression in *Caenorhabditis elegans*. G3 (Bethesda) *9*, 2195–2198.

Su, Z., Alhadeff, A.L., and Betley, J.N. (2017). Nutritive, post-ingestive signals are the primary regulators of AgRP neuron activity. Cell Rep. *21*, 2724–2736.

Sun, J., Liu, Y., and Aballay, A. (2012). Organismal regulation of XBP-1-mediated unfolded protein response during development and immune activation. EMBO Rep. *13*, 855–860.

Sun, J., Singh, V., Kajino-Sakamoto, R., and Aballay, A. (2011). Neuronal GPCR controls innate immunity by regulating noncanonical unfolded protein response genes. Science *332*, 729–732.

Tao, J., Ma, Y.C., Yang, Z.S., Zou, C.G., and Zhang, K.Q. (2016). Octopamine connects nutrient cues to lipid metabolism upon nutrient deprivation. Sci. Adv. *2*, e1501372.

Taylor, R.C., and Dillin, A. (2013). XBP-1 is a cell-nonautonomous regulator of stress resistance and longevity. Cell *153*, 1435–1447.

Walter, P., and Ron, D. (2011). The unfolded protein response: from stress pathway to homeostatic regulation. Science *334*, 1081–1086.

Williams, K.W., Liu, T., Kong, X., Fukuda, M., Deng, Y., Berglund, E.D., Deng, Z., Gao, Y., Liu, T., Sohn, J.W., et al. (2014). Xbp1s in pomc neurons connects ER stress with energy balance and glucose homeostasis. Cell Metab. *20*, 471–482.

Wragg, R.T., Hapiak, V., Miller, S.B., Harris, G.P., Gray, J., Komuniecki, P.R., and Komuniecki, R.W. (2007). Tyramine and octopamine independently inhibit serotonin-stimulated aversive behaviors in *Caenorhabditis elegans* through two novel amine receptors. J. Neurosci. *27*, 13402–13412.

Yemini, E., Jucikas, T., Grundy, L.J., Brown, A.E., and Schafer, W.R. (2013). A database of *Caenorhabditis elegans* behavioral phenotypes. Nat. Methods *10*, 877–879.

Zauner, C., Schneeweiss, B., Kranz, A., Madl, C., Ratheiser, K., Kramer, L., Roth, E., Schneider, B., and Lenz, K. (2000). Resting energy expenditure in short-term starvation is increased as a result of an increase in serum norepinephrine. Am. J. Clin. Nutr. *71*, 1511–1515.

Zhang, Q., Wu, X., Chen, P., Liu, L., Xin, N., Tian, Y., and Dillin, A. (2018). The mitochondrial unfolded protein response is mediated cell-non-autonomously by Retromer-dependent Wnt signaling. Cell *174*, 870–883.e17.

STAR★METHODS

KEY RESOURCE TABLE

REAGENT or RESOURCE	SOURCE	IDENTIFIER
Chemicals, Peptides, and Recombinant Proteins		
Fetal bovine serum	GIBCO, Life Technologies	Cat#10270106
Trizol LS	Ambion, Life Technologies	Cat#10296010
SYBRGreen Master Mix	Applied Biosystems	Cat#4472897
TrueCut Cas9 Protein v2	Invitrogen	Cat#36499
AltR tracrRNA	IDT	Cat#1073189
LR Clonase II Plus enzyme	Invitrogen	Cat#12538120
Gateway BP Clonase II Enzyme mix	Invitrogen	Cat#11789100
Sodium azide	Sigma Aldrich	Cat#S2002
Papain	AppliChem GmbH	Cat#A3824
Phusion High-Fidelity DNA Polymerase	New England BioLabs	Cat#M0530L
PureLink HQ Mini Plasmid DNA Purification kit	Invitrogen	Cat#K210001
Fetal bovine serum	GIBCO, Life Technologies	Cat#10270106
Trizol LS	Ambion, Life Technologies	Cat#10296010
Pertussis toxin	Sigma Aldrich	Cat# P7208
SYTO12	ThermoFisher	Cat#S7574
Critical Commercial Assays		
Direct-zol kit	Zymo Research	Cat#R2060
NuGEN Ovation RNaseq v2 kit	NuGEN	Cat#7102
Agilent High Sensitivity DNA kit	Agilent	Cat#5067-4626
Ovation Ultralow System v2 kit	NuGEN	Cat#0344
KAPA Library Quantification Kit	Illumina Platforms	Cat#07960140001
Agilent RNA 6000 Pico Kit	Agilent	Cat#5067-1513
QuantiTect reverse transcription kit	QIAGEN	Cat#205310
T3 mMessage mMachine transcription kit	Thermo Fischer Scientific	Cat#AM1348
GeneJET RNA purification kit	Thermo Fischer Scientific	Cat#K0731
Deposited Data		
NCBI	BioProject	PRJNA595861
Bacterial Strains		
E. coli OP50	CGC	WB OP50; RRID:WB-STRAIN:OP50
E. coli HT115	CGC	WB HT115; RRID:WB-STRAIN:HT115
L4440 RNAi	Addgene	Cat#1654
octr-1 RNAi	Ahringer library, Source Bioscience	Cat#3318_Cel_RNAi_complete
Caenorhabditis elegans strains		
See Table S2		
Xenopus laevis oocytes		
Defolliculated *Xenopus laevis* oocytes	EcoCyte Bioscience	https://ecocyte-us.com/products/xenopus-oocyte-delivery-service/
Oligonucleotides		
See Table S3		
Software and Algorithms		
SeqMonk	Babraham Institute	https://www.bioinformatics.babraham.ac.uk/projects/seqmonk/
g:Profiler	ELIXIR	https://biit.cs.ut.ee/gprofiler/index.cgi

(*Continued on next page*)

Continued

REAGENT or RESOURCE	SOURCE	IDENTIFIER
C. elegans tissues-specific gene database	Princeton University	http://worm-tissue.princeton.edu/search/download
Guide RNA design tool	MIT	crispr.mit.edu
Prism	Graphpad	https://www.graphpad.com/scientific-software/prism/
RoboCyte2 control software	Multichannel systems	Roboocyte2 control program
RoboCyte2+ analysis software	Multichannel systems	Roboocyte2+ analysis program
TEVC analysis scripts	Github	https://github.com/hiris25/TEVC-analysis-scripts

RESOURCE AVAILABILITY

Lead Contact

Further information and requests for resources and reagents should be directed to and will be fulfilled by the Lead Contact, Rebecca Taylor (rtaylor@mrc-lmb.cam.ac.uk).

Materials Availability

C. elegans strains generated in this study are available from the Lead Contact without restriction.

Data and Code Availability

RNA-seq data generated during this study have been deposited to NCBI: BioProject number PRJNA595861..

EXPERIMENTAL MODEL AND SUBJECT DETAILS

C. elegans Maintenance

C. elegans strains were maintained at 20°C on nematode growth medium (NGM) plates seeded with OP50 bacteria (Brenner, 1974). For feeding RNAi experiments either L4440 empty vector or the designated RNAi bacteria was used (Kamath et al., 2001). Plates for RNAi analysis were prepared by supplementation of agar with 100 μg/mL carbenicillin and 1mM IPTG after autoclaving. 24 h prior to each assay plates were spotted with 100 μL of overnight bacterial culture.

Xenopus Laevis Oocyte Maintenance

Defolliculated *Xenopus laevis* oocytes were purchased from EcoCyte Bioscience (Dortmund, Germany) and upon arrival stored in ND96 buffer at 16° C (in mM: 96 NaCl, 1 $MgCl_2$, 5 HEPES, 1.8 $CaCl_2$, 2 KCl).

METHOD DETAILS

C. elegans Cell Dissociation

Using animals with a neuronal (RCT37 and RCT38) or an intestinal (RCT51, RCT52 and RCT53) GFP marker, neuronal or intestinal cells were isolated as described (Imanikia et al., 2019a). Briefly, eggs isolated with hypochlorite treatment were allowed to grow to day 1 of adulthood, at which stage age-matched adult worms were washed several times to remove bacteria. Cuticles were then permeabilized using a TritonX100-SDS-DTT solution, followed by enzymatic digestion with papain (10 mg/mL) and mechanical disruption using an electronic hand homogenizer (IKA T10 basic, ULTRA-TURRAX). Cells were then recovered in fetal bovine serum (FBS) solution. Samples to be used as whole-worm comparisons were not processed any further, while cells destined for sorting were kept on ice prior to preparation for FACS.

C. elegans Cell Isolation by FACS

Neuronal cell suspensions were passed through a 5μm syringe filter (Millipore), while intestinal cell suspensions (in PBS/2% FBS) were passed through a 35 μm syringe filter and a 40 μm nylon mesh filter (Falcon). Filtered cells were then diluted in the same media as above and sorted using a Sony iCyt Synergy Dual Channel high speed cell sorter (488 nm excitation). Gates were used to eliminate cells with tdTomato and autofluorescence. GFP positive fluorescent events were collected in 1.5 mL Eppendorf tubes containing 10-20 μL PBS/2% FBS, and cells were kept on ice for RNA extraction. Collected positive fluorescent events varied between 20,000 and 60,000 events for intestinal samples, and between 100,000 and 300,000 events for neuronal samples.

RNA Extraction and Amplification

FACS-sorted cells were centrifuged and cell volumes normalized to 100 μL. Immediately 400 μL of Trizol LS was added to the tubes which were then snap frozen in liquid nitrogen. RNA was extracted, DNase-digested and cleaned using a microprep Direct-zol kit (Zymo Research). Initial RNA quantities for downstream analysis were normalized to 1.5 ng. Agilent Bioanalyser RNA Pico chips were used to assess the quantity and quality of RNA. Amplified cDNA was generated using the NuGEN Ovation RNAseq v2 kit, and DNA quality and quantity evaluated using an Agilent High Sensitivity DNA kit and Nanodrop (Nanodrop 2000c, Thermo Scientifics). cDNA was then sheared using a Covaris M220 sonicator (Covaris Inc.) to an average size of 150-200bp.

Library Preparation and RNA Sequencing

Library preparation was performed using the NuGEN Ovation® Ultralow System v2 kit, and library profiles checked using Agilent Bioanalyzer High Sensitivity DNA Chips. Neuronal and intestinal samples were pooled separately and diluted to 15 nM for sequencing single-end for 50 bp on an Illumina HiSeq4000 (CRUK CI, Cambridge). A single lane on the flowcell was used for the neuronal RNA-seq samples, with three replicates per control and experimental strain. Two lanes were used for the intestinal RNA-seq samples, with 3 replicates each for the control and two experimental strains. Reads were trimmed using Trim Galore (v0.4.5, cutadapt 1.15) and mapped to *C. elegans* WBcel235 using Hisat2 (v2.1.0). rRNA reads were removed and analysis performed using SeqMonk (https://www.bioinformatics.babraham.ac.uk/projects/seqmonk/). Differentially expressed genes were identified using DeSeq2 analysis. Gene ontology analysis was performed using g:Profiler (https://biit.cs.ut.ee/gprofiler/index.cgi), with a custom-created background gene list based on genes expressed in neuronal cells of *rab-3p::xbp-1s* worms, with $\log_2$ RPKM values between 0-100. Upregulated and downregulated gene categories were separated for GO analyses, and a ranked gene list, in which the first gene had the highest fold-change, was used. In order to understand the expression levels of tissue-specific genes (neurons, intestine, muscle), we identified tissue-specific genes from the Princeton database of tissue-specific expression predictions for *C. elegans* (http://worm-tissue.princeton.edu/search/download), where only the genes with a score of 1 for the relevant tissue and a score of 0 for all the remaining tissues were chosen for each tissue category.

Assessment of Binding Site Frequency

Genomic locations of XBP-1s binding motifs (ACGT core, CCACG box, UPRE A, UPRE B) were determined using FIMO sequence scanning (default settings, database 'Ensembl Genomes and Proteins, *Caenorhabditis elegans*, version 96') (Grant et al., 2011). Motif occurrences were counted for each promoter region (+/- 100 bp around the transcription start site). The mean count for each motif was calculated for the sets of genes upregulated and downregulated in *xbp-1s*-expressing relative to control neurons, and compared to the mean count of 2000 random sets of promoters. To exclude bias towards the many very short *C. elegans* genes, only genes longer than 500 bp were considered (761 upregulated, 734 downregulated, 750 random genes).

Quantitative RT-PCR

Total RNA was extracted as described above and 2 ng of purified RNA was used for cDNA synthesis using a QuantiTect reverse transcription kit. SYBRGreen quantitative RT-PCR was performed using the Corbett Rotor-Gene 6000. Fold changes were calculated using the $2\Delta\Delta Ct$ method, with 3 biological repeats for each sample in each comparison. Significance was assessed by one-way ANOVA with Dunnett's or Tukey's multiple comparisons test, or unpaired student's t-test when only single comparisons were made.

Generation of Transgenic C. elegans Strains

The Multisite Gateway Three-Fragment Cloning System (Invitrogen) was used to generate plasmids containing a transgenic element for ectopic expression. Plasmids containing promoters for *tbh-1* and *tdc-1* in the position 1 pENTRY vector were a kind gift from Dr Denise Walker. mKate2 in position 2 pENTRY vector was a kind gift from Dr Yiquan Tang. *Xbp-1s* cDNA was cloned into a position 2 pDONR vector upon PCR amplification from the pRT2 plasmid (encoding *rab-3p::xbp-1s*). The *xbp-1* promoter and genomic DNA were amplified from N2 worms and cloned into the position 1 and position 2 pENTRY vectors, respectively. The *unc-54* 3'UTR and *let-858* 3'UTR in the position 3 pENTRY vector were kindly provided by Dr. Mario De Bono. These pENTRY vectors were then used in LR reactions with the pDEST vector. At each stage plasmids were purified using the PureLink HQ Mini Plasmid DNA Purification kit (Invitrogen) after electroporation and liquid culture. The resulting expression clones were microinjected into the *C. elegans* gonad to form extrachromosomal array lines. Where genomic integration was performed, UV irradiation was used and the resulting integrated lines outcrossed at least 6 times.

Protein-Based CRISPR for Mutagenesis

We generated CRISPR deletion lines using a protocol based on a previously developed methodology (Paix et al., 2015). crispr.mit.edu was used as a guide design tool for selecting crRNAs complementing the site immediately 5' upstream of the PAM sequence adjacent to the site targeted by Cas9. Guide RNA generation was completed by mixing 0.5 μL of tracrRNA (IDT) at 4 μg/μL with 2.8 μL of crRNA at 100 μM, with Duplex buffer (IDT) added to the 5 μL mark. 20 μL microinjection mixes were prepared containing 1 μL of Cas9 protein (IDT) at 5 mg/mL, both of the tracrRNA/crRNA complexes at 1 μL, and homology directed repair template (single-stranded oligodeoxynucleotide molecules guiding repair upon Cas9-mediaed cleavage) at 1 μL (100μM). *Dpy-10* crRNA and HDR template were also used as a co-CRISPR marker to assess successful transformation after microinjection. This mutation was

crossed out once CRISPR-based deletion was confirmed. All CRISPR deletions used cuts generated at two sites via two crRNAs to generate alleles similar to reference alleles, apart from *eat-4*, for which only one crRNA was designed to introduce a single cut.

Fluorescence Microscopy

Worms were analyzed for GFP intensity at day 1 adult of adulthood, unless otherwise noted, following age synchronization by hypochlorite treatment of gravid parental worms, or by timed egg-lay. Worms were anaesthetized using 100 mM sodium azide (Sigma) and micrographs were acquired using a Leica M205 FA microscope and LAS X software. Each micrograph contained at least 5 individual worms, and was independently replicated at least 3 times. Fluorescence intensity was quantified using ImageJ or Fiji; background fluorescence was subtracted before normalizing to area, and fluorescence was then normalized to the control strain. A Student's t-test was used to assess pairwise significance between strains; one-way ANOVA with Tukey's multiple comparisons test was used to make multiple comparisons.

Confocal Microscopy

For live microscopy worms were mounted on a 2% agarose pad andanaesthetized using sodium azide (50 mM). Images were acquired on a Zeiss LSM 710 confocal microscope using the 20x air, or 40x and 63x oilimmersion objectives. A single section was acquired for all imaging and the pinhole used was 1 AU for optimal section thickness using the smartsetup function. Images were analyzed using Fiji.

All confocal microscopy analysis was independently replicated at least 3 times.

Lifespan Analysis

Lifespan analyses were performed at 20°C and were repeated at least three times. Typically, a minimum of 100 animals was used per condition, and worms were scored for viability every second day, from day 1 of adulthood (treating the pre-fertile day preceding adulthood as t = 0). Lifespans were performed on *E. coli* OP50, and animals were treated with 100 μg/mL FUDR at t = 0 and again at day 5 of adulthood, to avoid issues associated with early death through vivipary ("bagging") in *rab-3p::xbp-1s* animals. Prism 8 software was used for statistical analysis, and significance calculated using the log-rank (Mantel–Cox) method.

Dietary Deprivation Assays

Dietary deprivation assays were conducted and analysed as lifespan assays (above) with the difference that, after growth to early day 1 of adulthood on *E. coli* OP50, animals were transferred to plates containing 100 μg/mL carbenicillin and not seeded with bacteria, and significance was calculated using the Gehan-Breslow-Wilcoxon method.

Gene Expression in *Xenopus* Oocytes

cDNA sequences for *Xenopus* oocyte expression were cloned into the KSM vector backbone containing *Xenopus* β-globin UTR regions and a T3 promoter.

RNA Synthesis and Microinjection

Plasmids were linearized using NotI before *in vitro* synthesis of 5' capped cRNA using the T3 mMessage mMachine transcription kit according to manufacturer's protocol (Thermo Fischer Scientific). The cRNA was purified using the GeneJET RNA purification kit (Thermo Fischer Scientific). *Xenopus* oocytes were placed individually into 96-well plates and injected with 50 nL of a total 500 ng/uL RNA using the Roboinject system (Multi Channel Systems GmbH). Injected oocytes were incubated at 16° C in ND96 until the day of recording, typically between 1-2 days post injection. The GPCR-containing plasmids were co-injected with plasmids containing different mouse G-protein inward rectifying potassium channels (GIRKs) in order to record changes in current upon GPCR activation. This was done in a 1:2 ratio (GIRK:GPCR). For evaluation of G-protein identity, 50 pg of pertussis toxin (PTX) was injected into previously injected oocytes, 6h prior to recording.

Two-Electrode Voltage Clamp Recording

Two-electrode voltage clamp (TEVC) recordings were performed using the Robocyte2 system (Multi Channel Systems). Glass electrodes were backfilled with 1.5 M KCl and 1 M acetic acid. Oocytes were clamped at -80 mV unless stated otherwise. Continuous recordings were taken during application of a high K^+ solution (96 mM KaCl, 1 mM MgCl2, 5 mM HEPES, 1.8 mM CaCl2, 2 mM NaCl) and agonists at 500 Hz. Data were recorded using RoboCyte2 control software.

Recordings were performed as previously described for GIRK channels (Hardege et al., 2015). Briefly, oocytes were perfused with ND96 to estimate resting potential of the oocytes followed by 40s perfusion of high K^+ buffer. Agonists were applied in high K^+, for 10s and followed by a 30s ND96 wash out. Dose response data were collected in the same continuous recording set up, with increasing agonist concentrations applied step wise for 10s each in high K^+, without a wash period in between. Data were gathered over at least two occasions, using different batches of oocytes.

TEVC Data Analysis and Plotting

Peak current was analyzed with Robocyte2+ software, taking the peak current during the window of interest. Dose response curves were generated using a custom-built python script which combined data from multiple recordings and normalised by calculating

CellPress
OPEN ACCESS

I/Imax for each oocyte (https://github.com/hiris25/TEVC-analysis-scripts). Normalised mean, SD and n numbers where then imported into GraphPad Prism where data were plotted and EC_{50} values were calculated by fitting to the Hill equation using a four-parameter slope. Ratios of agonist induced currents in PTX vs. non PTX injected oocytes was calculated and plotted in GraphPad Prism, and significance was calculated using an unpaired Student's t-test.

Worm Speed Measurement

Twenty 16-hour post-L4 worms were individually plated on low-peptone plates seeded with 20 μL of OP50 bacteria grown overnight in LB, and each plate was individually tracked using the Schafer Lab WormTracker 2.0 video-tracking system (Yemini et al., 2013). Tracking started after a 30-minute adaptation period for each plate with a single worm, and lasted for 15 min. Videos were analyzed using custom MatLab software (https://www.mrc-lmb.cam.ac.uk/wormtracker/), taking into account stage movement, segmentation and feature extraction. Prism 8 software was used for statistical analysis, and significance of differences between N2 and *rab-3p::xbp-1s* worms were calculated using unpaired Student's t-test.

Food Leaving Assay

The assay used was adapted from one previously published (Milward et al., 2011). 20 adult worms were placed on 2 day old 35 mm low-peptone NGM plates seeded with 25 μL of overnight OP50 culture in 2XTY. Worms were assayed 18 h post-L4, using 3 technical replicates for each condition. Animals were video-recorded for 15 min at 0, 3, 6 and 9 h, and scored manually as leaving the food patch only if their whole body was excluded from the food patch with no immediate retraction back to the bacterial lawn. Food leaving probability was then calculated as # of worms leaving per minute/ # of total worms at the start of that minute, averaged over 15 min. Each assay was independently replicated at least 2-3 times using 20 animals per replicate, and significance between conditions determined using two-way ANOVA with Tukey's multiple comparison test.

Off-Food Exploration Assay

This assay was adapted from one previously published (Pradhan et al., 2019). 10 μl of OP50 grown overnight in LB was seeded on 55 mm NGM plates. A single worm (16 h post-L4) was placed for an hour on the bacterial lawn, after which it was removed and the plate incubated at 37 °C to seed the growth of bacteria on the regions explored by the worm outside the food patch. Bacterial growth was then scored across a grid, with bacterial growth in the 9 0.25 cm^2 squares nearest to the food patch assigned a score of 1, the next 25 squares outside the first area assigned a score of 2, the next 49 squares outside the second area assigned a score of 3, the next 81 squares outside the third area assigned a score of 4, and finally the next 121 squares outside the fourth area assigned a score of 5. Each assay was independently replicated 6 times with at least 4 technical replicates each time, using a single animal per replicate, and significance between conditions determined using one-way ANOVA with Dunnett's multiple comparison test.

On-Food Exploration Assay

This assay was also adapted from one previously published (Pradhan et al., 2019). 400 μl of OP50 grown overnight in LB was seeded, to cover the entire surface area of a 55 mm NGM plate. A single worm (16 h post-L4) was placed on the OP50 and allowed to explore for 6 h, after which the worm was removed and the area travelled (detected by trails on the bacterial lawn) calculated against a grid of 0.25 cm^2 squares. The number of squares travelled in this grid was counted and the exploration noted as a percentage of the full plate area. Each assay was independently replicated 7 times with at least 3 technical replicates each time, using a single animal per replicate, and significance between conditions determined using one-way ANOVA with Dunnett's multiple comparison test.

Brood Size Assay

50 worms were placed onto individual NGM plates 18 h post-L4 to lay eggs. Worms were transferred twice a day onto new plates for the first 3 days of adulthood, and the number of progeny hatched on each plate was manually counted to define brood size.

Eggs *In Utero* and Egg Staging Assays

Worms 30 h post-L4 were individually soaked in hypochlorite solution (20%) until the cuticle disappeared and eggs were accessible. The number and/or stage of each egg was assessed as previously described (Ringstad and Horvitz, 2008) using a dissecting microscope. Each assay was independently replicated 5 times, with each biological replicate containing 10 gravid worms, and significance was determined by one-way ANOVA with Dunnett's multiple comparison test.

Germline Apoptosis Assays

Time course analysis of the number of apoptotic germ cells using SYTO12 dye was performed as previously described (Gumienny et al., 1999; Levi-Ferber et al., 2014). Briefly, worms were grown to L4, D1 and D4 adult, and incubated in 33 μM aqueous solution of SYTO12 for 4.5 h at room temperature on an orbital rotator. Thereafter, worms were transferred to seeded plates in order to clear excessive dye from the intestine, for 30-60 min. Animals were then mounted on 2% agarose pads and anesthetised using a drop of sodium azide (50 mM). Micrographs were acquired using a Zeiss LSM 710 confocal microscope with 63x oil immersion objectives. A single section was acquired for all imaging and the pinhole used was 1 AU for optimal section thickness using the smart setup function. Image analysis was conducted using Fiji, and assays repeated 3 times, using 6-10 worms per strain in each replicate. Significance was calculated by one-way ANOVA with Tukey's multiple comparisons test.

QUANTIFICATION AND STATISTICAL ANALYSIS

Tests used to determine statistical significance were two-way ANOVA with Tukey's or Sidak's multiple comparisons test (food leaving assays), one-way ANOVA with Tukey's multiple comparisons test (RT-qPCR, Q40 fluorescence analysis, colocalization assays, germline apoptosis assays), one-way ANOVA with Dunnett's multiple comparisons test (RT-qPCR, tissue/neuron-specific gene expression, exploration assays, and eggs *in utero* assays), the Mantel-Cox log-rank test (lifespan, internal hatching assays), the Gehan-Breslow-Wilcoxon test (dietary deprivation assays), and Student's t-test (all other assays). DeSeq2 analysis was used to define significance in RNA-seq experiments. Statistical information for each experiment can be found in the corresponding figure legend.

Developmental Cell, Volume *55*

Supplemental Information

Tyramine Acts Downstream of Neuronal XBP-1s to Coordinate Inter-tissue UPR^{ER} Activation and Behavior in *C. elegans*

Neşem P. Özbey, Soudabeh Imanikia, Christel Krueger, Iris Hardege, Julia Morud, Ming Sheng, William R. Schafer, M. Olivia Casanueva, and Rebecca C. Taylor

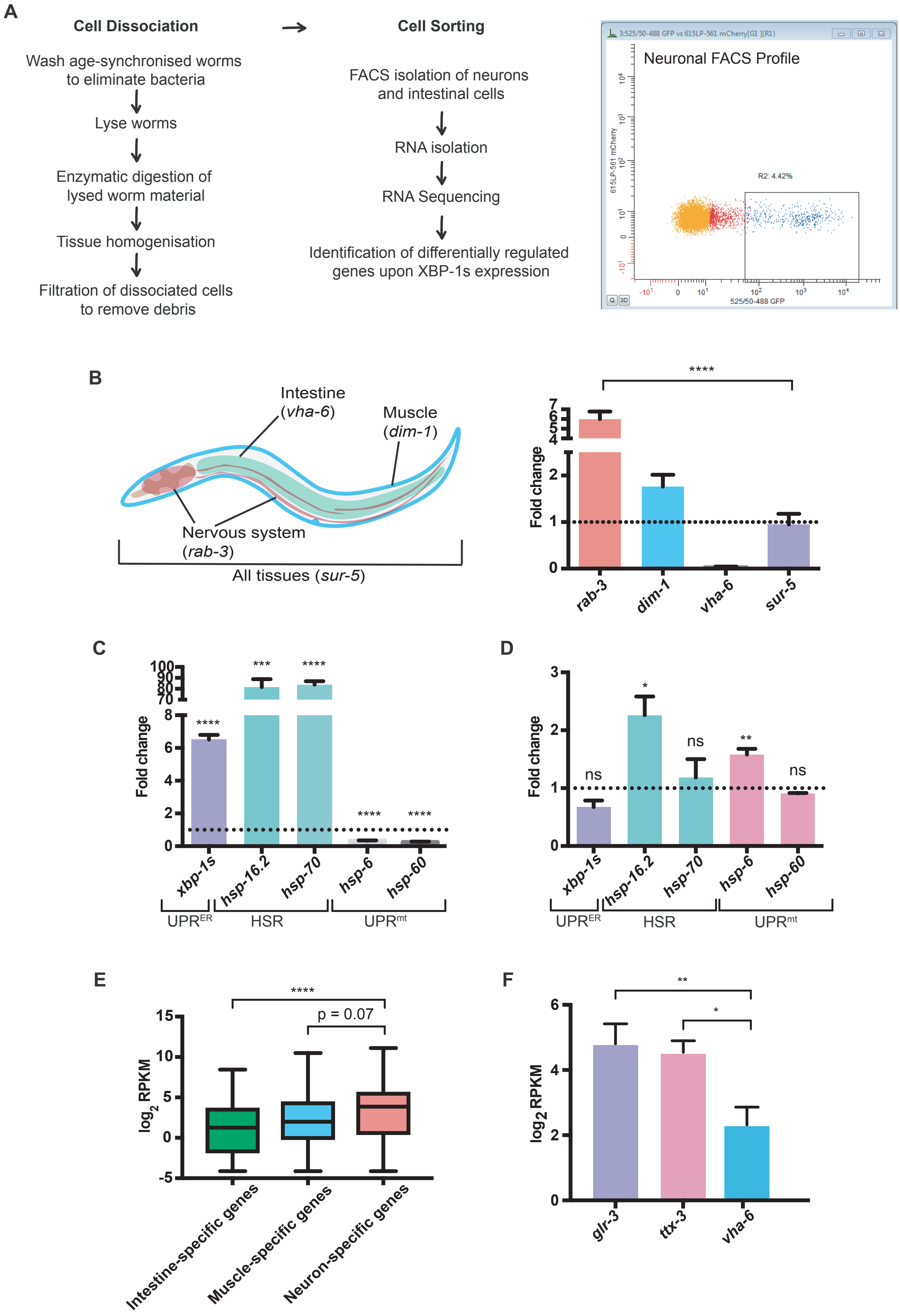
A
Cell Dissociation
Wash age-synchronised worms to eliminate bacteria
Lyse worms
Enzymatic digestion of lysed worm material
Tissue homogenisation
Filtration of dissociated cells to remove debris
Cell Sorting
FACS isolation of neurons and intestinal cells
RNA isolation
RNA Sequencing
Identification of differentially regulated genes upon XBP-1s expression
Neuronal FACS Profile
R2: 4.42%
615LP-561 mCherry
525/50-488 GFP
B
Intestine (vha-6)
Muscle (dim-1)
Nervous system (rab-3)
All tissues (sur-5)
Fold change
rab-3
dim-1
vha-6
sur-5
****
C
Fold change
xbp-1s
hsp-16.2
hsp-70
hsp-6
hsp-60
UPR^ER
HSR
UPR^mt
D
Fold change
ns
*
ns
**
ns
E
log2 RPKM
****
p = 0.07
Intestine-specific genes
Muscle-specific genes
Neuron-specific genes
F
log2 RPKM
**
*
glr-3
ttx-3
vha-6

Figure S1. Validation of neuron-specific RNA-seq analysis. Related to Figure 1.

(A) Experimental outline of adult cell dissociation for tissue-specific RNA-seq. An example FACS profile for cells prepared for neuronal RNA-seq highlights the GFP population isolated from dissociated *unc-119p::GFP* worms.

(B) Tissue-specific transcript levels determined by RT-qPCR in samples of isolated neuronal cells from *unc-119p::GFP* worms compared to dissociated but unsorted cells of the same strain. Fold change was calculated relative to the all-tissue transcript *sur-5*, and error bars indicate SEM (N=3 biological replicates). Significance was assessed by one-way ANOVA with Dunnett's multiple comparisons test, ****$p \leq 0.0001$.

(C) RT-qPCR analysis of *xbp-1s, hsp-16.2, hsp-70, hsp-6* and *hsp-60* transcripts in dissociated and FACS-sorted GFP-positive neuronal cells from *unc-119p::GFP* worms, versus dissociated and FACS-sorted GFP-negative non-neuronal cells. Fold change was calculated for each transcript relative to wild type, and error bars indicate SEM (N=3 biological replicates). Significance of the difference between levels of each transcript in wild type and neuronal *xbp-1s*-expressing animals was assessed by unpaired student's t-test, ***$p \leq 0.001$, ****$p \leq 0.0001$.

(D) RT-qPCR analysis of *xbp-1s, hsp-16.2, hsp-70, hsp-6* and *hsp-60* transcripts in dissociated and FACS-sorted, recombined cell samples from *unc-119p::GFP* worms, versus cells that were dissociated but not FACS-sorted. Fold change was calculated for each transcript relative to wild type, and error bars indicate SEM (N=3 biological replicates). Significance of the difference between levels of each transcript in wild type and neuronal *xbp-1s*-expressing animals was assessed by unpaired student's t-test, *$p \leq 0.05$, **$p \leq 0.01$, ns=not significant.

(E) Expression levels of tissue-specific genes (intestine, muscle and nervous system) in the neuronal RNA-seq dataset of *unc-119p::GFP; rab-3p::xbp-1s* animals. Tissue-

specific genes were identified using the Princeton tissue-specific database. RPKM = reads per kilobase per million mapped reads, N=3 biological replicates, and error bars indicate SEM. Significance was calculated relative to neuron-specific genes by one-way ANOVA with Dunnett's multiple comparisons test, ****$p \leq 0.0001$.

(F) Levels of 2 transcripts expressed in no more than two pairs of neurons in the neuronal RNA-seq dataset of *unc-119p::GFP; rab-3p::xbp-1s* animals. N=3 biological replicates, error bars indicate SEM. Significance was calculated relative to levels of the intestinal (non-neuronal) transcript *vha-6* by one-way ANOVA with Dunnett's multiple comparisons test, *$p \leq 0.05$, **$p \leq 0.01$.

XBP-1-dependent INDUCIBLE UPR genes

Gene Function	Up in neuronal *xbp-1s*	Down in neuronal *xbp-1s*	Gene names (differentially regulated by neuronal *xbp-1s*)
**COP1 coated vesicle**	1/1	0/1	Y41C4A.11 (Coatomer β subunit)
**Protein disulphide isomerases**	3/4	0/4	C14B9.2, B0403.4, PDI-2
**Chaperones**	7/10	0/7	DNJ-7, DNJ-28, HSP-3, HSP-4, T14G8.3 (HSP70), T24H7.2 (HSP70), Y57A10A.23 (ERp19)
**Ubiquitination**	2/3	0/3	UBC-13, H40L08.1
**Vesicle docking**	3/6	0/6	F55A4.1 (Sec22b), F18H3.4 (integrin), D1014.3 (Snap-1)
**ARF + interacting proteins**	3/7	0/7	F54C8.7 (Arfaptin), Y54G2A.2 (ARL-6 interacting protein 2), K02B12.7 (ARF1-directed GAP)
**Oxidative stress**	2/5	0/5	K01D12.11 (Glutathione S-transferases), Y76B12C.3 (Selenoprotein)
**Cell growth & cell death**	2/5	0/5	Y54G2A.18 (BAP31), C04F12.1 (Piwi-domain containing protein)
**Quality control and ERAD**	2/9	0/9	NSF-1, ERD-2
**Gene expression**	2/9	0/9	F57B10.1 (CREBh), RRF-2
**Lipid & phospholipid metabolism**	2/12	0/12	B0285.9 (choline kinase), ACL-12
**Signal transduction**	1/6	0/6	DLK-1 (MAPK12)
**Ion channels and transporters**	1/9	0/9	T28F3.3 (zinc transporter)
**Sugar modification**	0/7	0/7	
**Sugar synthesis**	0/7	0/7	
**COPII coated vesicle**	0/6	0/6	
**Signal peptidases**	0/5	0/5	
**Calcium homeostasis**	0/5	0/5	
**Mitochondrial homeostasis**	0/4	0/4	
**Protein interaction**	0/4	0/4	
**Clathrin vesicle coat**	0/3	0/3	
**Cytoskeleton**	0/3	0/3	
**Cuticle synthesis**	0/3	0/3	
**Protein folding – other**	0/2	0/2	
**Host defence**	0/2	0/2	
**Phosphate mobilization**	0/1	0/1	
**Purine metabolism**	0/1	0/1	

XBP-1-dependent CONSTITUTIVE UPR genes

Gene Function	Up in neuronal *xbp-1s*	Down in neuronal *xbp-1s*	Gene names (differentially regulated by neuronal *xbp-1s*)
**Sugar transport**	2/2	0/2	H17B01.1, NLP-14 (Neuropeptide-Like Protein)
**Cell fate – other**	1/1	0/1	ZK1073.1 (Differentiation-related gene 1)
**Intracellular trafficking**	2/4	0/4	T12A2.15b (Ca2+-dependent membrane-targeting module), NCS-2 (neuronal calcium sensor protein)
**DNA topoisomerase**	0/2	1/2	ZK1127.7 (DNA topoisomerase II)
**RNA helicase**	0/2	1/2	CGH-1
**RNA synthesis**	0/3	1/3	GLD-2 (poly(A) polymerase)
**Exocytosis**	1/3	0/3	C39F7.2
**Tumor Suppressor**	1/3		MPZ-1 (Multiple PDZ domain protein)
**G protein related**	2/9	1/9	Up: GPB-1 (G beta subunit), GOA-1 (G protein alpha subunit); Down: T19E10.1b (transforming protein etc2)
**Cytoskeleton**	1/9	1/9	Up: PTL-1 (microtubule-associated protein TAU-1a); Down: C53C9.2 (thin filament-associated protein calponin)
**Carbohydrate metabolism**	1/5	0/5	CAH-3 (carbonic anhydrase)
**Lipid metabolism**	1/5	0/5	AAK-2 (metabolic stress-sensing kinase AMPK α2 chain)
**Hormone signalling**	1/5	0/5	INS-30 (Insulin related)
**Cell adhesion & junction**	1/8	0/8	UNC-9 (innexin)
**Ser/Thr kinase**	1/10	0/10	EGL-4 (cyclic-GMP-dependent protein kinase)
**Protein synthesis**	0/5	0/5	
**Ub ligase complex**	0/4	0/4	
**Ub conjugating complex**	0/3	0/3	
**Proteasome regulatory particle**	0/2	0/2	
**Proteases**	0/2	0/2	
**ER & Golgi translocation**	0/8	0/8	
**Vacuole targeting**	0/2	0/2	
**Endocytosis**	0/2	0/2	
**Mitochondrial import**	0/1	0/1	
**Nucleo-cytoplasmic transport**	0/7	0/7	
**Energy production**	0/4	0/4	
**Purine biosynthesis**	0/1	0/1	
**UDP-glucuronosyl transferase**	0/2	0/2	
**Homo-cystinuria**	0/2	0/2	
**Transcription factor**	0/13	0/13	
**Translation initiation**	0/3	0/3	
**mRNA splicing**	0/4	0/4	
**RNA processing and modification**	0/4	0/4	
**mRNA catabolism**	0/4	0/4	
**Gene expression - other**	0/2	0/2	
**Nucleogenesis**	0/1	0/1	
**Histone**	0/1	0/1	
**Amino acid transport**	0/1	0/1	
**Lipid + fatty acid transport**	0/4	0/4	
**Transporters – other**	0/2	0/2	
**Calcium transport**	0/3	0/3	
**Ion transport**	0/5	0/5	
**Tyr kinase**	0/1	0/1	
**Ser/Thr phosphatase**	0/7	0/7	
**Signal transduction – other**	0/2	0/2	
**Axon guidance**	0/1	0/1	
**Cell division & cycle**	0/6	0/6	
**Meiosis**	0/1	0/1	
**Apoptosis**	0/1	0/1	
**Mitochondrial function**	0/2	0/2	

Figure S2. Comparison between genes differentially regulated upon neuronal *xbp-1s* expression and XBP-1 targets previously identified by microarray. Related to Figure 1.

A comparison of genes found to be differentially regulated in an XBP-1-dependent manner upon UPR^{ER} induction by tunicamycin (INDUCIBLE) or constitutively (CONSTITUTIVE) using microarray analysis (Shen et al., 2005), with genes differentially regulated in neurons upon neuronal *xbp-1s* expression in our RNA-seq analysis.

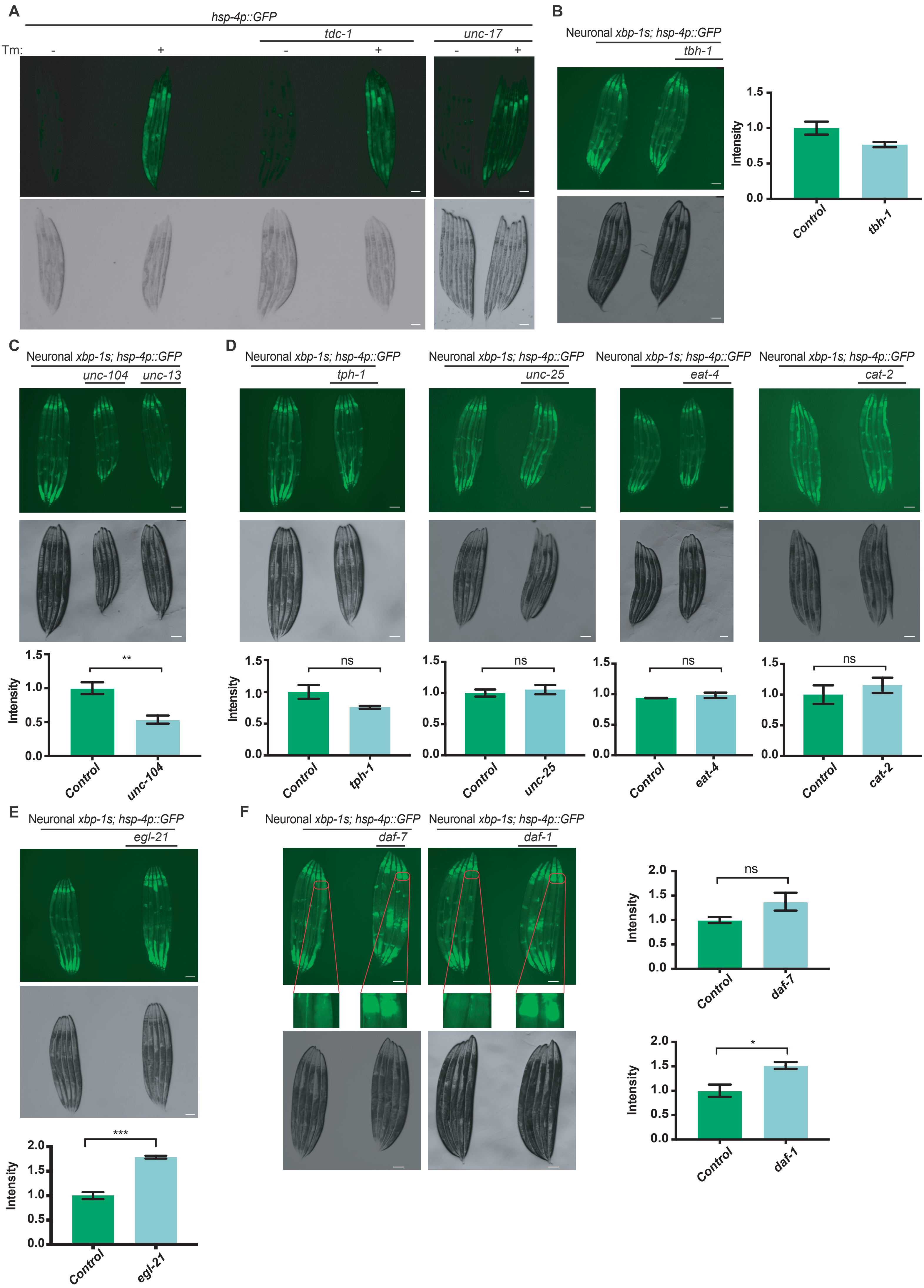

A
hsp-4p::GFP
tdc-1
unc-17
Tm:
-
+
B
Neuronal xbp-1s; hsp-4p::GFP
tbh-1
Intensity
Control
tbh-1
C
Neuronal xbp-1s; hsp-4p::GFP
unc-104
unc-13
**
D
tph-1
unc-25
eat-4
cat-2
ns
E
egl-21
***
F
daf-7
daf-1
*

Figure S3. The effects of mutations in neurotransmitter-associated genes on UPR^{ER} activation. Related to Figure 2.

(A) Fluorescence microscopy of *rab-3p::xbp-1s; hsp-4p::GFP*, *unc-17(e245); rab-3p::xbp-1s; hsp-4p::GFP*, and *tdc-1(n3419); rab-3p::xbp-1s; hsp-4p::GFP* animals treated with 2% DMSO or 25 mM tunicamycin for 4 hours. N=3 biological replicates. Scalebars = 100 μm.

(B) Fluorescence microscopy of an additional CRISPR-derived *tbh-1* mutant allele, *tbh-1(rms8)*, in the *rab-3p::xbp-1s; hsp-4p::GFP* genetic background. GFP levels were quantified using ImageJ and normalized to control *rab-3p::xbp-1s; hsp-4p::GFP* animals. N=3 biological replicates each containing ≥5 animals, error bars indicate SEM. Scalebars = 100 μm.

(C) Fluorescence microscopy of *unc-104(e1265)* and *unc-13(e450)* mutant alleles in the *rab-3p::xbp-1s; hsp-4p::GFP* genetic background. GFP levels were quantified using ImageJ and normalized to control *rab-3p::xbp-1s; hsp-4p::GFP* animals, and significance assessed relative to control worms by unpaired student's t-test, **p≤0.01. N≥3 biological replicates each containing ≥5 animals, error bars indicate SEM. Scalebars = 100 μm.

(D) Fluorescence microscopy of *tph-1(mg280), unc-25(e156), eat-4(rms5)* and *cat-2(e1112)* mutant alleles in the *rab-3p::xbp-1s; hsp-4p::GFP* genetic background. GFP levels were quantified using ImageJ and normalized to control *rab-3p::xbp-1s; hsp-4p::GFP* animals, and significance assessed relative to control worms by unpaired student's t-test, ns=not significant. N≥3 biological replicates each containing ≥5 animals, error bars indicate SEM. Scalebars = 100 μm.

(E) Fluorescence microscopy of *egl-21(n611)* mutant allele in the *rab-3p::xbp-1s; hsp-4p::GFP* genetic background. GFP levels were quantified using ImageJ and

normalized to control *rab-3p::xbp-1s; hsp-4p::GFP* animals, and significance assessed relative to control worms by unpaired student's t-test, ***p≤0.001. N≥3 biological replicates each containing ≥5 animals, error bars indicate SEM. Scalebars = 100 μm.

(F) Fluorescence microscopy of *daf-7(e1372)* and *daf-1(m40)* mutant alleles in the *rab-3p::xbp-1s; hsp-4p::GFP* genetic background. GFP levels were quantified using ImageJ and normalized to control *rab-3p::xbp-1s; hsp-4p::GFP* animals, and significance assessed relative to control worms by unpaired student's t-test, *p≤0.05, ns=not significant. N≥3 biological replicates each containing ≥5 animals, error bars indicate SEM. Scalebars = 100 μm.

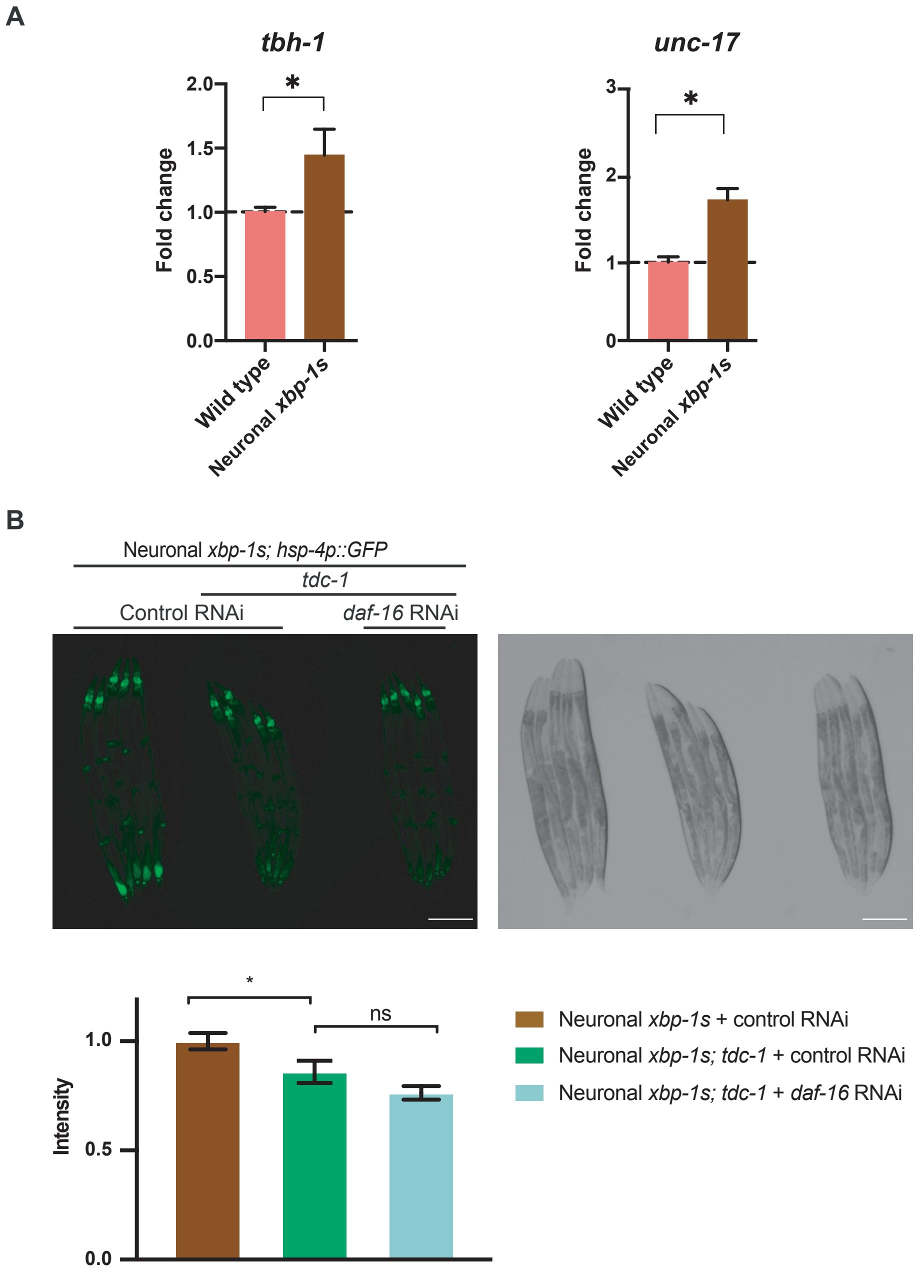
A
tbh-1
unc-17
Fold change
Wild type
Neuronal xbp-1s
B
Neuronal xbp-1s; hsp-4p::GFP
tdc-1
Control RNAi
daf-16 RNAi
Intensity
ns
Neuronal xbp-1s + control RNAi
Neuronal xbp-1s; tdc-1 + control RNAi
Neuronal xbp-1s; tdc-1 + daf-16 RNAi

Figure S4. Neuronal *xbp-1s* upregulates *tbh-1* and *unc-17*, and its effects on intestinal UPR^{ER} activation do not depend on *daf-16*. Related to Figure 2.

(A) RT-qPCR analysis of *tbh-1* and *unc-17* expression in whole worm samples from wild type or *rab-3p::xbp-1s* animals. Fold change was calculated for each transcript relative to wild type, and error bars indicate SEM (N=4 biological replicates). Significance of the difference between levels of each transcript in wild type and neuronal *xbp-1s*-expressing animals was assessed by one-way ANOVA with Tukey's multiple comparisons test, $^{*}p \leq 0.05$.

(B) Fluorescence microscopy of *rab-3p::xbp-1s; hsp-4p::GFP* and *rab-3p::xbp-1s; hsp-4p::GFP; tdc-1(n3419)* animals treated with control or *daf-16* RNAi from hatch. GFP levels were quantified using ImageJ and normalized to control *rab-3p::xbp-1s; hsp-4p::GFP* animals, and significance assessed relative to control worms by unpaired student's t-test, $^{*}p \leq 0.05$, ns=not significant. N=2 biological replicates each containing ≥5 animals, error bars indicate SEM. Scalebars = 250 μm.

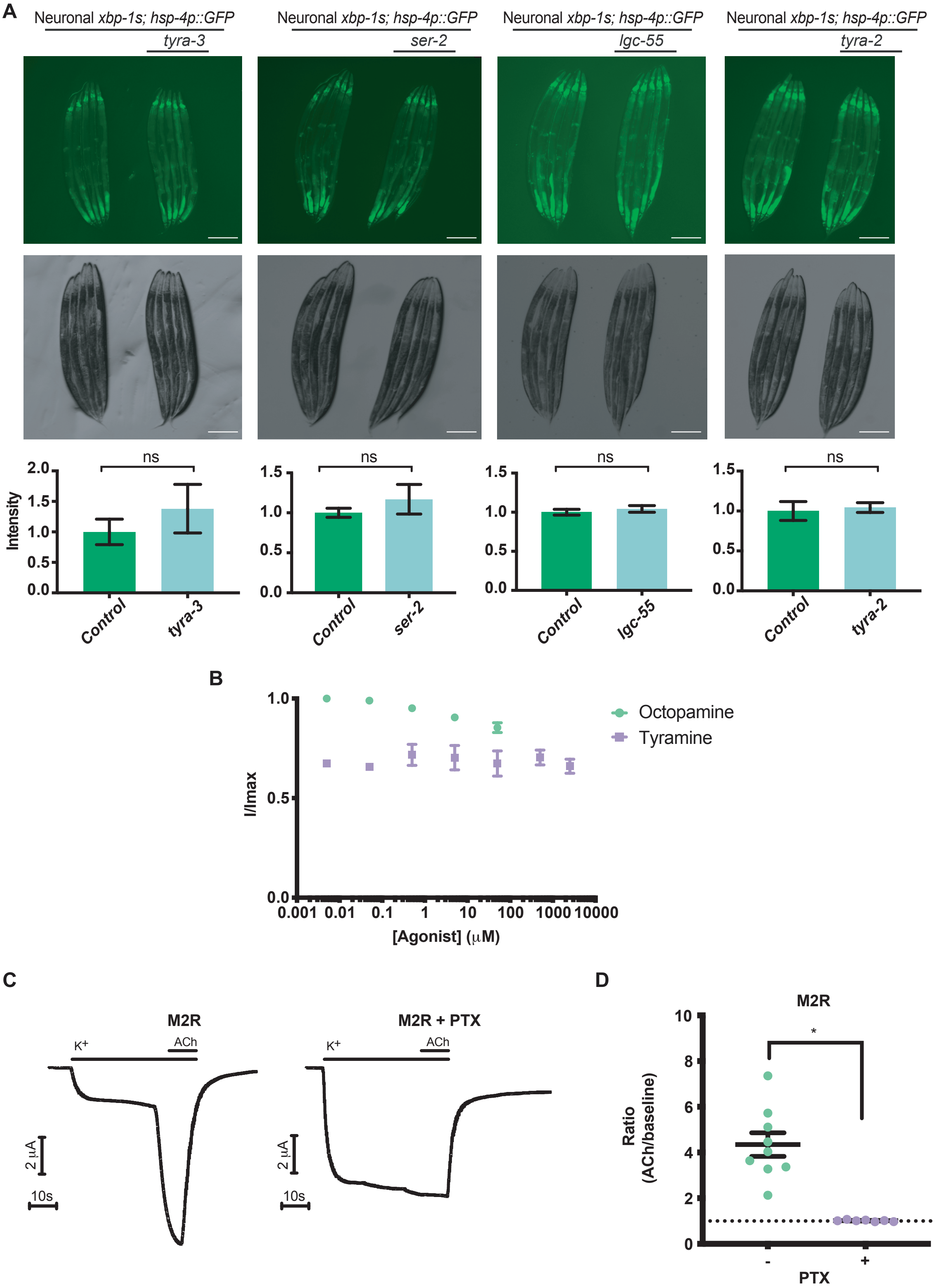

A
Neuronal xbp-1s; hsp-4p::GFP
tyra-3
ser-2
lgc-55
tyra-2
Intensity
ns
Control
B
Octopamine
Tyramine
I/Imax
[Agonist] (μM)
C
M2R
M2R + PTX
K+
ACh
2 μA
10s
D
M2R
Ratio (ACh/baseline)
PTX

Figure S5. Analysis of candidate tyramine and octopamine receptors in *rab-3p::xbp-1s; hsp-4p::GFP* animals. Related to Figure 3.

(A) Fluorescence microscopy of *tyra-3(rms1), ser-2(rms3), lgc-55(rms7)* and *tyra-2(rms6)* mutant alleles in the *rab-3p::xbp-1s; hsp-4p::GFP* genetic background. GFP levels were quantified using ImageJ and normalized to control *rab-3p::xbp-1s; hsp-4p::GFP* animals, and significance assessed relative to control worms by unpaired student's t-test, ns=not significant. N≥3 biological replicates each containing ≥5 animals, error bars indicate SEM. Scalebars = 250 μm.

(B) Octopamine and tyramine failed to induce a response in *Xenopus laevis* oocytes expressing only mGIRK1 and mGIRK2. Error bars represent SEM of 3 oocytes. EC_{50} was calculated by fitting data to the Hill equation using a four-parameter variable slope.

(C) Continuous two-electrode voltage clamp (TEVC) recordings from untreated or PTX-injected *Xenopus laevis* oocytes expressing M2R, mGIRK1 and mGIRK2 and treated with 100nM acetylcholine (Ach).

(D) Current ratios of acetylcholine-evoked vs basal high K^+ current for at least 7 oocytes expressing M2R, mGIRK1 and mGIRK2 injected with or without PTX. Error bars represent mean ± SEM. Significance was assessed by unpaired student's t-test, *p≤0.05.

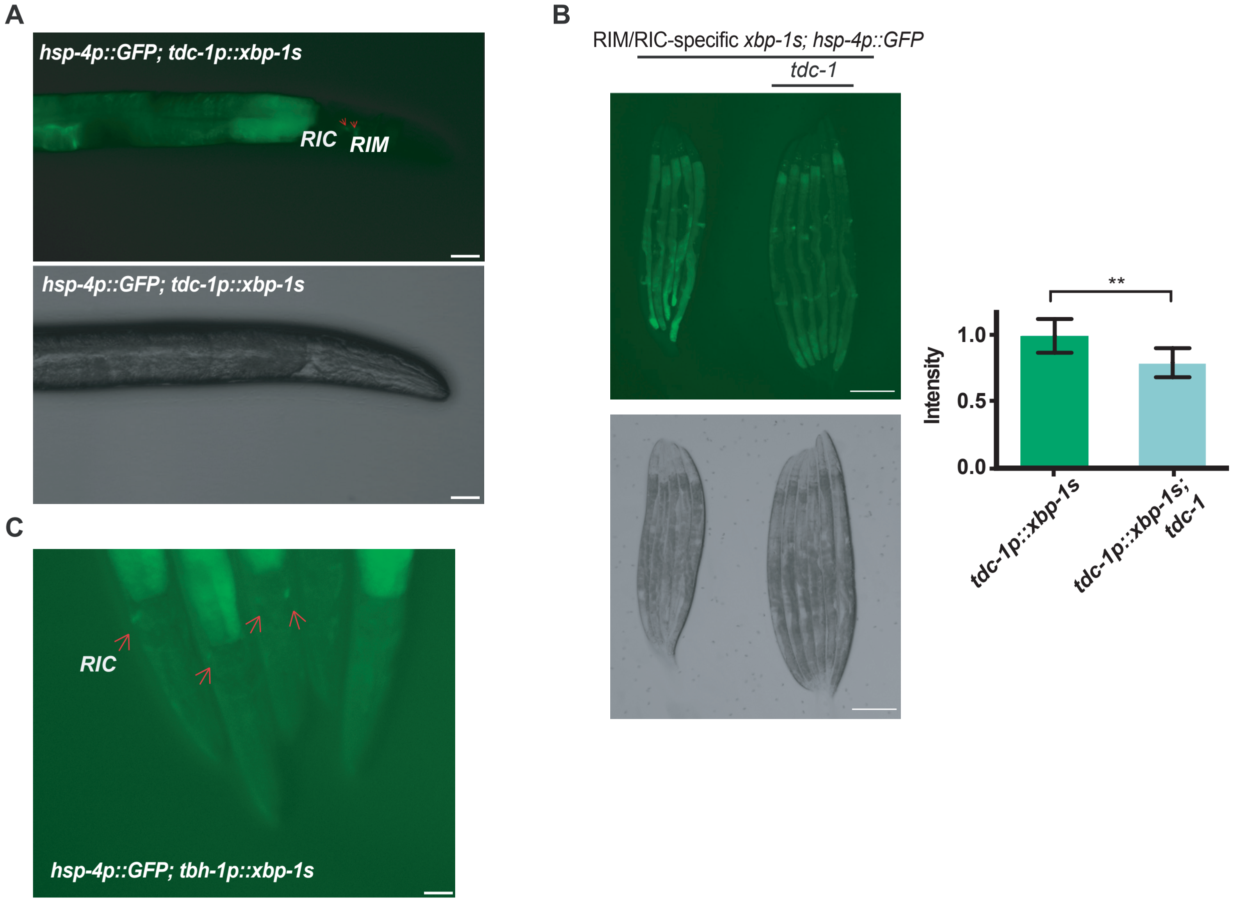
A
hsp-4p::GFP; tdc-1p::xbp-1s
RIC
RIM
hsp-4p::GFP; tdc-1p::xbp-1s
B
RIM/RIC-specific xbp-1s; hsp-4p::GFP
tdc-1
**
Intensity
1.0
0.5
0.0
tdc-1p::xbp-1s
tdc-1p::xbp-1s; tdc-1
C
RIC
hsp-4p::GFP; tbh-1p::xbp-1s

Figure S6. Validation of the functional expression of *xbp-1s* in RIC and RIM interneurons. Related to Figure 4.

(A) Fluorescence microscopy of a *tdc-1p::xbp-1s; hsp-4p::GFP* animal showing GFP fluorescence in the RIC and RIM interneurons. Arrows indicate the RIC and RIM neurons. Scalebars = 20 μm.

(B) Fluorescence microscopy of *tdc-1p::xbp-1s; hsp-4p::GFP* animals with and without a *tdc-1(n3419)* mutation. GFP levels were quantified using ImageJ and normalized to *tdc-1p::xbp-1s; hsp-4p::GFP* animals, and significance assessed relative to control worms by unpaired student's t-test, $^{**}p \leq 0.01$. N=3 biological replicates each containing ≥5 animals, error bars indicate SEM. Scalebars = 250 μm.

(C) Fluorescence microscopy of a *tbh-1p::xbp-1s; hsp-4p::GFP* animal showing GFP fluorescence in the RIC interneurons. Arrows indicate the RIC neurons. Scalebars = 20 μm.

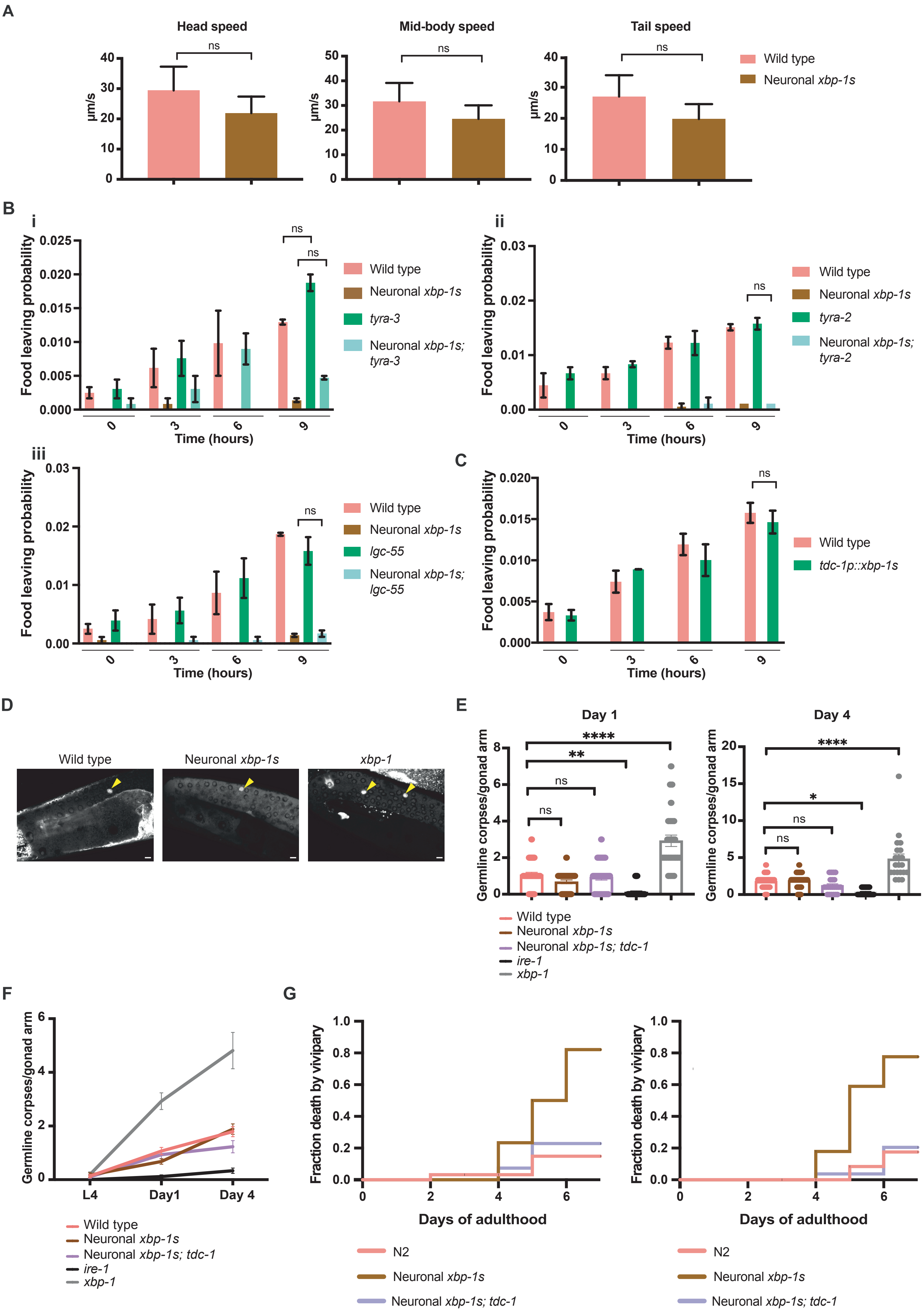
A
Head speed
Mid-body speed
Tail speed
ns
μm/s
Wild type
Neuronal xbp-1s
B
i
ii
iii
Food leaving probability
Time (hours)
tyra-3
Neuronal xbp-1s; tyra-3
tyra-2
Neuronal xbp-1s; tyra-2
lgc-55
Neuronal xbp-1s; lgc-55
C
tdc-1p::xbp-1s
D
Wild type
Neuronal xbp-1s
xbp-1
E
Day 1
Day 4
Germline corpses/gonad arm
****
**
*
Neuronal xbp-1s; tdc-1
ire-1
F
L4
Day1
Day 4
G
Fraction death by vivipary
Days of adulthood
N2

Figure S7. *rab-3p::xbp-1s* worms are normally motile, mutations in candidate tyramine and octopamine receptors do not affect their food leaving propensity, and these animals do not display increased levels of germline apoptosis. Related to Figures 5 and 6.

(A) Head speed, mid-body speed and tail speed of *rab-3p::xbp-1s* compared to N2 worms was measured using a WormTracker. Graphs represent average speed in ~20 worms, and error bars indicate SEM. Significance was assessed by unpaired student's t-test, ns=not significant.

(B) Food leaving probability was calculated in (i) *tyra-3(rms1),* (ii) *tyra-2(rms6)* and (iii) *lgc-55(rms7)* animals with and without *rab-3p::xbp-1s* expression, alongside N2 and *rab-3p::xbp-1s* animals. 20 adult worms per strain were placed on 35 mm plates seeded with a drop of OP50 and video-recorded for 15 minutes at t=0, 3, 6, and 9 hours to assess probability of food leaving. Mean probability was then plotted, with error bars representing SEM (N≥3). Significance was assessed as indicated at the final timepoint by two-way ANOVA with Tukey's multiple comparison test, ns=not significant.

(C) Food leaving probability was calculated and plotted as described above in *tdc-1p::xbp-1s* and N2 animals. Error bars represent SEM (N≥3), and significance was assessed between the two strains at the final timepoint by two-way ANOVA with Sidak's multiple comparison test, ns=not significant.

(D) Apoptotic cell corpses in the germlines of N2, *rab-3p::xbp-1s* and *xbp-1(tm2457)* animals were stained with SYTO12 and imaged by confocal microscopy. Images show animals at day 1 of adulthood, arrows indicate apoptotic corpses. Scalebars = 5 μm.

(E) Apoptotic corpses in the germline of N2, *rab-3p::xbp-1s*, *rab-3p::xbp-1s;tdc-1(n3419)*, *ire-1(v33)* and *xbp-1(tm2457)* animals stained with SYTO12 and imaged as above were quantified and the average number of corpses per gonadal arm at day 1 and day 4 of adulthood plotted (N=3, 6-10 worms per strain in each replicate). Significance was calculated by one-way ANOVA with Tukey's multiple comparison test, *p<0.05, **p<0.01, ****p<0.0001, ns=not significant.

(F) Apoptotic corpses in the germline of N2, *rab-3p::xbp-1s*, *rab-3p::xbp-1s;tdc-1(n3419)*, *ire-1(v33)* and *xbp-1(tm2457)* animals stained with SYTO12 and imaged as above were quantified and the average number of corpses per gonadal arm plotted against age, from the L4 larval stage to day 4 of adulthood. Error bars indicate SEM (N=3, 6-10 worms per strain in each replicate).

(G) The number of N2, *rab-3p::xbp-1s*, *tdc-1(n3419)*; *rab-3p::xbp-1s* and *tbh-1(rms2); rab-3p::xbp-1s* animals in which internal hatching was observed was counted for the first 6 days of adulthood and the fraction undergoing internal hatching plotted, in 2 additional biological replicates. Significance was calculated by Mantel-Cox log-rank test; p<0.0001 between *rab-3p::xbp-1s* and N2, and between *rab-3p::xbp-1s* and *rab-3p::xbp-1s*, *tdc-1(n3419)* in each case.
